# Revelation of Influencing Factors in Overall Codon Usage Bias of Equine Influenza Viruses

**DOI:** 10.1371/journal.pone.0154376

**Published:** 2016-04-27

**Authors:** Naveen Kumar, Bidhan Chandra Bera, Benjamin D. Greenbaum, Sandeep Bhatia, Richa Sood, Pavulraj Selvaraj, Taruna Anand, Bhupendra Nath Tripathi, Nitin Virmani

**Affiliations:** 1 Immunology Lab, National Institute of High Security Animal Diseases (NIHSAD), Bhopal, Madhya Pradesh, India; 2 Biotechnology Lab, Veterinary Type Culture Collection, National Research Center on Equines (NRCE), Hisar, Haryana, India; 3 Tisch Cancer Institute, Departments of Medicine, Hematology and Medical Pathology, and Pathology, Icahn School of Medicine at Mount Sinai, New York, New York, United States of America; 4 Equine Pathology Lab, National Research Center on Equines (NRCE), Hisar, Haryana, India; Centers for Disease Control and Prevention, UNITED STATES

## Abstract

Equine influenza viruses (EIVs) of H3N8 subtype are culprits of severe acute respiratory infections in horses, and are still responsible for significant outbreaks worldwide. Adaptability of influenza viruses to a particular host is significantly influenced by their codon usage preference, due to an absolute dependence on the host cellular machinery for their replication. In the present study, we analyzed genome-wide codon usage patterns in 92 EIV strains, including both H3N8 and H7N7 subtypes by computing several codon usage indices and applying multivariate statistical methods. Relative synonymous codon usage (RSCU) analysis disclosed bias of preferred synonymous codons towards A/U-ended codons. The overall codon usage bias in EIVs was slightly lower, and mainly affected by the nucleotide compositional constraints as inferred from the RSCU and effective number of codon (ENc) analysis. Our data suggested that codon usage pattern in EIVs is governed by the interplay of mutation pressure, natural selection from its hosts and undefined factors. The H7N7 subtype was found less fit to its host (horse) in comparison to H3N8, by possessing higher codon bias, lower mutation pressure and much less adaptation to tRNA pool of equine cells. To the best of our knowledge, this is the first report describing the codon usage analysis of the complete genomes of EIVs. The outcome of our study is likely to enhance our understanding of factors involved in viral adaptation, evolution, and fitness towards their hosts.

## Introduction

Equine influenza viruses (EIVs) are negative-sense, single-stranded, segmented RNA viruses within the family *Orthomyxoviridae*, and are a common cause of respiratory infections in horses. The octameric segmented genome encodes 11 or 12 proteins [1–2]. The standard genotypic classification relies on composition of the surface glycoproteins—haemagglutinin (HA) and neuraminidase (NA), which are also targets for development of vaccines and antiviral drugs. EIVs are thought to be of avian origin with aquatic birds as a key natural reservoir. Like the avian influenza viruses, they have an affinity for sialic acid α-2,3-galactose containing receptors on the host cell surfaces. Several combinations of HA and NA subtypes occur in birds, however, only two subtypes have so far been detected in horses, i.e. H3N8 and H7N7. The H7N7 viruses have not been isolated for over three decades and are considered to have disappeared [3]. Since 1979, almost all the outbreaks reported in equines across the world have been attributed to H3N8 viruses. The most striking feature of influenza viruses is their ability to evade the host immune response either by antigenic shift or reassortment [4–5]. Furthermore, they undergo rapid evolution when subjected to host immune selection pressure, especially while crossing the host species barrier [6]. Since influenza viruses are completely dependent on the host cellular machinery for their replication, their adaptability in a particular host depends on their codon usage preferences. A detailed understanding of factors responsible for the EIVs codon usage preferences will help in delineating their evolution, and the relationship with the host immune response.

Redundancy of the genetic code allows the use of multiple codons for encoding a particular amino acid. The synonymous codons differ only at the third position [7]. Although synonymous codons do not change the amino acid sequences, their usage is not uniform, both within and between the organisms. This results in species—specific codon usage bias [8–9]. Thus, certain codons are preferred due to bias in the synonymous codon usage [10]. The selection of preferred codons has been linked to functional integrity, maintenance of the genetic code [11] as well as an important evolutionary force which determines the overall fitness by influencing various processes like RNA processing, level of gene expression, protein translation and protein folding [12–14]. As revealed from the population studies, the evolution of biased codon usage is dictated primarily by two major factors; first one is AU/GC biased mutational pressure while the second one is a weak selection acting on a specific subset of codons (preferred codons) [10,15–16]. Other factors like interaction between codons and anticodons [17], site specific codon biases [18], efficacy of replication [19], usage of codon pairs [20] and evolutionary time scale [21] also contribute in the codon usage bias.

Previous studies have extensively analyzed the factors responsible in shaping the codon usage bias in different RNA viruses, including influenza viruses. For examples, in H5N1 viruses, compositional constraint was the key determinant responsible for most of the variations in the synonymous codon usage with little contribution from gene function and no role of translational selection and gene length [22]. Wong et al. [23] identified mammalian-like viral codon usage patterns in 1918 pandemic H1N1 virus genes and indicated the role of host selection pressure on the directional changes in codon usage over the time of virus isolation. Additional similar study on 1918 pandemic influenza A viruses highlighted their possible origin from classical swine H1N1 viruses based on Relative Synonymous Codon Usage (RSCU) and the Effective Number of Codons (ENc) values of PB1 gene [24]. Overall, mutation pressure played a key role in shaping the codon usage bias in influenza A virus strains (H1N1) from the pandemic of 2009 [25], while synonymous codon usage patterns of Neuraminidase (NA) gene of H1N1 strains isolated from Canada was dictated by natural selection [26]. Different nucleotide compositions of avian and human influenza A viruses were shown to be dictated by host-dependent mutation bias [27]. In addition, dinucleotide usage has been shown to have host dependencies and influence mutational bias, even when codon usage bias and protein coding are accounted for [28–29]. One study described that codon usage of PB1-F2 gene of EIV was less biased, and governed by three main factors, i.e. mutational bias, selection pressure, and gene length [30]. However, a comprehensive analysis of factors governing the codon usage patterns in EIVs genomes has not been elucidated so far.

Therefore, we systematically analyzed the synonymous codon usage patterns in 92 EIV strains isolated from 1963 to 2013 for which complete genomic sequences are available. The purpose of the study was to gain insight into the influencing factors accountable for shaping the codon usage patterns in EIV genomes, trends in their codons usage over the time and possible elucidation of H7N7 disappearance.

## Materials and Methods

### Sequence data

Complete genomic sequences of equine influenza viruses (EIVs), including all subtypes isolated from 1963 to 2013 across the world were obtained from the Influenza Virus Resource at the National Center for Biotechnological Information (http://www.ncbi.nlm.nih.gov/genomes/FLU/FLU.html). The data set comprised the complete coding genome sequences of 92 EIV strains. For each strain, open reading frames (ORFs) were concatenated in the following order (PB2 + PB1 + PA + HA + NP + NA + MP + NS). Out of total 92 strains, 83 belonged to H3N8 and 9 to H7N7 subtype. The data set comprised a total of 4,19,774 codons. For details of strains used in this study, see S1 Table.

### Codon usage bias measurement

#### Nucleotide composition analysis

The frequencies of occurrence of each nucleotide (A %, U %, C %, and G %); each nucleotide at the third position of the synonymous codons (A3%, U3%, C3%, and G3%); G+C at the first (GC1), second (GC2), and third codon positions (GC3); G+C at the first and the second positions (GC1,2); and the overall AU% and GC% content were calculated for each strain of EIV.

#### Relative synonymous codon usage (RSCU)

The RSCU value of a codon is the ratio of its observed frequency to its expected frequency given that all codons for a particular amino acid are used equally [31]. RSCU values are not affected by sequence length and amino acid frequency since these factors are eliminated during the computation. The RSCU values were calculated using previously described method [31] as given below in the equation:

$$RSCU=\frac{g_{ij}}{\sum_{j}^{ni}g_{ij}}ni$$

Where g_ij_ is the observed number of the i^th^ codon for the j^th^ amino acid which has ni kinds of synonymous codons. Codons with RSCU value of <1.0, 1.0, >1.0 represent negative codon usage bias, no bias and positive codon usage bias, respectively.

#### Relative dinucleotides frequencies

The dinucleotide frequencies for each strain of EIV, which is another way of establishing the relation with codon usage bias, were calculated as described previously [32]. Expected dinucleotide values were also calculated assuming random association of bases from the observed frequencies of each base for every sequence. A ratio of actual to expected dinucleotide frequencies was used for designation of over-representation (>1.23) or under-representation (<0.78) in terms of relative abundance compared with a random association of mononucleotides.

#### Codon free dinucleotide frequencies and selective force on dinucleotides

We calculated the difference between the expected number of dinucleotides and the observed number when both the sequence codon usage bias and the primary amino acid sequence are accounted for as constraints. In this way we could tell whether the forces exerted on dinucleotides in a sequence were due to amino acid changes or codon usage bias. We did so using the methods described in the previous studies [28,33]. The selective force on a dinucleotide is the entropic cost of changing the number of occurrences of a dinucleotide given a set of constraints, in this case it is the cost given the amino acid sequence and codon usage bias of a sequence [28,33]. The force on a dinucleotide is calculated using a transfer-matrix method adapted from statistical physics [33].

#### Effective number of codons (ENc)

The ENc is frequently used to measure the magnitude of codon usage bias of a gene. We calculated ENc to evaluate the degree of codon usage bias in the coding sequences of EIVs. The ENc was calculated using the formula given below:

$$ENc=2+\frac{9}{{\bar{F}}_{2}}+\frac{1}{{\bar{F}}_{{}_{3}}}+\frac{5}{{\bar{F}}_{4}}+\frac{3}{{\bar{F}}_{6}}$$

Where F_(i = 2,3,4,6)_ is the mean of F_i_ values for the i-fold degenerate amino acids. The F_i_ values were calculated using the formula given below:

$${\bar{F}}_{i}=\frac{n\sum_{j=1}^{i}{\left(\frac{n_{j}}{n}\right)}^{2}-1}{n-1}$$

Where n is the total number of occurrences of the codons for that amino acid and n_j_ is the total number of occurrences of the j^th^ codon for that amino acid. The ENc values range from 20 to 61 [34]. The ENc value of 20 indicates a gene with an extreme codon usage bias (only one of the possible synonymous codons is used for the corresponding amino acid), while 61 for a gene showing no bias (all possible synonymous codons are used equally for the corresponding amino acid). Therefore, the smaller the ENc value, the greater will be the extent of codon usage bias. In general, a gene is thought to possess strong codon bias if its ENc value is equal or less than 35 [34,35].

### Mutational pressure mediated codon usage bias

#### ENc-plot

An ENc-plot is generally used to find out the determining factors (especially mutation bias/mutation pressure) that influence the codon usage bias. The ENc values are plotted against the GC3s values (frequency of either a guanine or cytosine at the third codon position of the synonymous codons, excluding Met, Trp, and stop codons) [34]. In genes, where codon usage is constrained only by G+C mutation bias, predicted ENc values will lie on or around the standard curve (functional relation between expected ENc and GC3s). Else, if predicted ENc values lie far lower than the standard curve, then other factors such as natural selection play a major role in shaping the codon usage bias. Expected ENc values were calculated as given in the equation below:

$$ENc_{expected}=2+s+\frac{29}{s^{2}+(1-s^{2})}$$

Where ‘s’ is the frequency of G + C at the third codon position of synonymous codons (i.e. GC3s).

### Natural selection mediated codon usage bias

#### Neutrality plot

A neutrality plot (GC12 Vs GC3) was used to investigate the mutation-selection equilibrium in shaping the codon usage bias [36]. Neutrality plot was drawn with GC12 as ordinate and GC3 as abscissa, and each dot represents an independent EIV strain. A plot regression with a slope of 0 (the points positioned on the parallel lines of the abscissa) indicates no effect of directional mutation pressure (complete selective constraints), while a slope of 1 (the points positioned on the diagonal line) is indicative of complete neutrality [36].

#### Codon adaptation index (CAI)

To study the codon usage preferences of EIVs in relation to the codon usage of different host species, the codon adaptation index (CAI) was employed [37]. Kruskal–Wallis and Dunn's multiple comparison tests were used to address any statistically significant differences among CAI values obtained in different comparisons.

#### General average hydropathicity (GRAVY) and aromaticity (AROMO)

The GRAVY index is the arithmetic mean of the sum of the hydropathic indices of each amino acid [38]. This index ranges from −2 to 2; where positive and negative values are indicative of hydrophobic and hydrophilic protein, respectively. AROMO value signifies the frequency of aromatic amino acids (Phe, Tyr, Trp). Both GRAVY and AROMO values are indices of amino acid usage, and the variation in amino acid compositions can influence the results of codon usage analysis.

### Phylogenetic analysis

A dendogram was constructed using 83 complete genomic sequences of H3N8 and 9 of H7N7 subtype isolated from 1963 to 2013 across the world. Evolutionary relationships were inferred by using neighbor-joining statistical method [39] with kimura-2-parameter substitution model implemented in MEGA software version 6 [40]. The bootstrap values with 1000 replicates are indicated at the nodes of the branches. The scale bar indicates nucleotide substitutions per site. For each strain, the following data are given: EIV type/species of origin/country of origin/strain name/year of isolation/subtype.

### Statistical analysis

#### Correspondence analysis (COA)

Correspondence analysis (COA) is a multivariate statistical analysis, and usually employed to study the codon usage patterns. Since there is a total of 59 synonymous codons (61 sense codons minus the unique Met and Trp codons), the degrees of freedom were reduced to 40 by removing variations caused by the unequal usage of amino-acids while generating a correspondence analysis of RSCU [41]. The major trends within the dataset were determined using measures of relative inertia, and strains ordered according to their positions along the axis of major inertia. COA was performed on the RSCU values in this study.

Spearman’s rank correlation analysis, linear regression analysis, Kruskal–Wallis and Dunn's multiple comparison tests were performed by XLSTAT Version 2015 and GraphPad Prism 6 (GraphPad Software, San Diego, California, USA).

#### Software and databases

Nucleotide composition, dinucleotide composition, G + C at synonymous variable third position of codons (GC3s), relative synonymous codon usage (RSCU), and correspondence analysis were calculated using the program CodonW 1.4.2 (by John Peden and available at http://sourceforge.net/projects/codonw/) [42]. However, we computed the Effective Number of Codons (ENc) for each EIV strain using INCA2.1 [43]. Codon usage data of EIV clinical hosts; horse (*Equus caballus*) and donkey (*Equus asinus*) were obtained from the codon usage database (available at: http://www.kazusa.or.jp/codon/) [44]. Codon usage data for human (*Homo sapiens*), domestic pig (*Sus scrofa*), mallard (*Anas platyrhynchos*), goose (*Anser anser*), red jungle fowl (*Gallus gallus*), dog (*Canis familiaris*), mice (*Mus musculus*) and macaque (*Macaca fascicularis*) were also retrieved from the same database for comparative study of the relative codon usage preferences of EIVs. Codon Adaptation Index (CAI) was calculated using the approach of Puigbo et al. [45] (available at: http://genomes.urv.es/CAIcal/). The frequencies of tRNAs in equine cells were retrieved from the GtRNAdb database [46].

## Results and Discussion

### Nucleotide Composition Analysis

In the present study, we have analyzed 92 complete coding genomic sequences of EIVs comprising of total 12,59,322 nucleotides. Nucleotide composition analysis revealed that the mean A% (34.02) was the highest, and C% (19.39) being the lowest (S2 Table). The mean AU composition was 56.91%. This appears to suggest that there might be more usage of A nucleotides among the codon of EIVs. However, more insight into the nucleotide composition analysis, especially at the third codon position (A3, U3, G3, C3, GC3 and AU3), revealed that the mean A3% (33.55) was the highest (S2 Table). The AU3 values ranged from 53.6% to 65.7%, with a mean of 57.1% and a standard deviation (SD) of 2.21. Therefore, it is evident from the initial nucleotide composition analysis that A/U-ended codons might be preferred over G/C-ended codons in the EIV genomes.

### Relative Synonymous Codon Usage (RSCU) Analysis

To decrypt the extent by which A/U-ended codons might be preferred, and to determine the patterns of synonymous codon usage, RSCU values of each codon was calculated for 92 EIV strains and compared with different potential host species (Table 1).

**Table 1:** The Relative Synonymous Codon Usage (RSCU) patterns of EIVs and their potential hosts*.

		EIVs		Potential Hosts*							
AA	Codons	H3N8	H7N7	Donkey	Horse	Human	Pig	Duck	Goose	Chicken	Dog
Phe	UUU	0.88	0.97	0.88	0.83	0.93	0.79	0.81	0.99	0.91	0.82
	UUC	**1.12**	**1.03**	**1.12**	**1.17**	**1.07**	**1.21**	**1.19**	**1.01**	**1.09**	**1.16**
Leu	UUA	0.80	1.09	*0*.*24*	*0*.*33*	*0*.*46*	*0*.*32*	*0*.*35*	*0*.*39*	*0*.*45*	*0*.*35*
	UUG	**1.20**	0.91	0.82	0.72	0.77	0.67	0.71	0.71	0.81	0.68
	CUU	1.07	**1.15**	0.80	0.73	0.79	0.65	0.72	1.03	0.80	0.67
	CUC	0.96	0.88	***1*.*63***	1.32	1.17	1.35	1.27	1.06	1.08	1.25
	CUA	0.89	1.14	*0*.*13*	*0*.*34*	*0*.*43*	*0*.*33*	*0*.*34*	*0*.*40*	*0*.*38*	*0*.*37*
	CUG	1.08	0.84	***2*.*33***	***2*.*56***	***2*.*37***	***2*.*68***	***2*.*60***	***2*.*41***	***2*.*48***	***2*.*45***
Ile	AUU	**1.13**	1.10	*0*.*58*	0.92	1.08	0.91	0.97	0.97	1.06	0.96
	AUC	0.79	0.64	***1*.*95***	***1*.*66***	**1.41**	***1*.*67***	**1.55**	**1.55**	**1.39**	**1.61**
	AUA	1.08	**1.26**	*0*.*47*	*0*.*42*	*0*.*51*	*0*.*42*	*0*.*48*	*0*.*48*	*0*.*55*	*0*.*45*
Val	GUU	0.90	1.12	0.64	0.60	0.73	*0*.*57*	0.68	0.99	0.84	*0*.*58*
	GUC	0.78	0.65	1.42	1.08	0.95	1.07	1.05	0.71	0.87	1.10
	GUA	1.09	**1.14**	*0*.*29*	*0*.*35*	*0*.*47*	*0*.*34*	*0*.*43*	0.60	*0*.*50*	*0*.*42*
	GUG	**1.23**	1.09	***1*.*65***	***1*.*97***	***1*.*85***	***2*.*03***	***1*.*83***	***1*.*70***	***1*.*80***	***1*.*98***
Pro	CCU	1.04	1.21	0.83	1.19	1.15	1.05	0.95	**1.51**	1.10	1.08
	CCC	0.95	0.87	***1*.*60***	**1.38**	**1.29**	**1.46**	**1.50**	1.07	**1.22**	**1.47**
	CCA	**1.56**	***1*.*63***	1.06	0.97	1.11	0.94	1.05	1.14	1.13	1.05
	CCG	*0*.*45*	*0*.*30*	*0*.*51*	*0*.*45*	*0*.*45*	*0*.*56*	*0*.*51*	*0*.*29*	*0*.*56*	*0*.*51*
Thr	ACU	0.97	1.17	0.82	0.94	0.99	0.83	0.93	1.01	0.99	0.89
	ACC	0.93	0.88	***1*.*77***	**1.58**	**1.42**	***1*.*68***	**1.50**	***1*.*81***	**1.23**	**1.58**
	ACA	***1*.*84***	***1*.*79***	0.79	0.96	1.14	0.92	1.06	0.93	1.20	1.05
	ACG	*0*.*26*	*0*.*16*	0.61	*0*.*52*	*0*.*46*	*0*.*57*	*0*.*51*	*0*.*26*	*0*.*57*	*0*.*53*
Ala	GCU	0.96	0.92	1.20	1.05	1.06	0.96	1.20	***1*.*62***	1.16	1.0
	GCC	0.91	0.91	***1*.*74***	***1*.*72***	**1.60**	***1*.*80***	**1.34**	1.42	**1.27**	***1*.*78***
	GCA	***1*.*86***	***1*.*96***	0.77	0.77	0.91	0.74	1.02	0.75	1.06	0.81
	GCG	*0*.*27*	*0*.*21*	*0*.*30*	*0*.*45*	*0*.*42*	*0*.*50*	*0*.*44*	*0*.*21*	*0*.*51*	*0*.*47*
Tyr	UAU	**1.04**	**1.10**	0.63	0.75	0.89	0.73	0.67	0.77	0.80	0.79
	UAC	0.96	0.90	**1.37**	**1.25**	**1.11**	**1.27**	**1.33**	**1.23**	**1.20**	**1.15**
Ser	UCU	1.02	1.21	1.10	1.09	1.13	0.99	1.04	1.30	1.09	1.09
	UCC	0.92	0.94	**1.46**	1.43	1.31	1.50	1.24	1.44	1.21	1.52
	UCA	***1*.*68***	***1*.*65***	0.83	0.80	0.90	0.73	0.76	0.79	0.89	0.81
	UCG	*0*.*38*	*0*.*21*	*0*.*24*	*0*.*34*	*0*.*33*	*0*.*39*	*0*.*30*	*0*.*24*	*0*.*40*	*0*.*38*
	AGU	0.97	0.94	1.10	0.86	0.90	0.77	0.80	0.75	0.86	0.89
	AGC	1.03	1.06	1.27	**1.48**	**1.44**	***1*.*62***	***1*.*86***	**1.47**	**1.55**	**1.56**
Arg	AGA	1.36	1.48	1.49	1.30	**1.29**	1.12	1.29	***1*.*77***	**1.34**	1.20
	AGG	0.64	*0*.*52*	***1*.*86***	**1.32**	1.27	1.23	**1.41**	0.98	1.29	**1.32**
	CGU	*0*.*39*	*0*.*49*	*0*.*49*	*0*.*55*	*0*.*48*	*0*.*44*	0.63	0.63	*0*.*59*	*0*.*46*
	CGC	0.83	0.99	0.79	1.15	1.10	**1.31**	1.22	1.55	1.14	1.26
	CGA	***1*.*61***	**1.59**	0.74	0.61	0.65	0.60	*0*.*50*	*0*.*31*	*0*.*58*	0.67
	CGG	1.16	0.93	0.62	1.08	1.21	1.29	0.94	0.77	1.07	1.31
Cys	UGU	0.83	**1.02**	0.62	0.89	0.91	0.79	0.71	0.66	0.80	0.85
	UGC	**1.17**	0.98	**1.38**	**1.11**	**1.09**	**1.21**	**1.29**	**1.34**	**1.20**	**1.10**
His	CAU	**1.21**	**1.14**	0.89	0.81	0.84	0.70	0.73	0.80	0.80	0.78
	CAC	0.79	0.86	**1.11**	**1.19**	**1.16**	**1.30**	**1.27**	**1.20**	**1.20**	**1.22**
Gln	CAA	**1.11**	**1.29**	0.84	*0*.*52*	*0*.*53*	*0*.*44*	*0*.*57*	0.62	*0*.*54*	*0*.*50*
	CAG	0.89	0.71	**1.16**	**1.48**	**1.47**	**1.56**	**1.43**	**1.38**	**1.46**	**1.46**
Asn	AAU	**1.10**	**1.20**	0.66	0.84	0.94	0.79	0.79	**1.09**	0.86	0.87
	AAC	0.91	0.80	**1.34**	**1.16**	**1.06**	**1.21**	**1.21**	0.91	**1.14**	**1.12**
Lys	AAA	**1.32**	**1.40**	0.79	0.79	0.87	0.76	0.86	0.84	0.89	0.79
	AAG	0.68	*0*.*60*	**1.21**	**1.21**	**1.13**	**1.24**	**1.14**	**1.16**	**1.11**	**1.13**
Asp	GAU	**1.15**	**1.14**	0.84	0.83	0.93	0.80	0.90	0.90	**1.01**	0.86
	GAC	0.85	0.87	**1.16**	**1.17**	**1.07**	**1.20**	**1.10**	**1.10**	0.99	**1.09**
Glu	GAA	**1.26**	**1.35**	0.84	0.76	0.84	0.72	0.83	**1.02**	0.86	0.79
	GAG	0.74	0.65	**1.16**	**1.24**	**1.16**	**1.28**	**1.17**	0.98	**1.14**	**1.23**
Gly	GGU	0.66	0.70	0.87	0.65	0.65	*0*.*57*	0.64	0.73	0.70	0.65
	GGC	*0*.*53*	*0*.*44*	**1.42**	**1.43**	**1.35**	**1.46**	**1.25**	**1.31**	**1.22**	**1.45**
	GGA	***1*.*88***	***1*.*91***	0.85	0.95	1.00	0.91	0.96	1.21	1.09	1.02
	GGG	0.94	0.96	0.86	0.97	1.00	1.05	1.15	0.75	0.99	1.05
Trp	UGG	1.00	1.00	1.00	1.00	1.00	1.00	1.00	1.00	1.00	1.00
Met	UTG	1.00	1.00	1.00	1.00	1.00	1.00	1.00	1.00	1.00	1.00

Note: Preferentially used codons are displayed in bold; Over-represented (RSCU ≥ 1.6) and under-represented (RSCU ≤ 0.6) codons are marked as bold with italics and italics, respectively.

*Potential hosts include clinical hosts (horse, donkey), reservoir hosts (duck, goose, chicken), accidental hosts (dog) and others who might get infected by crossing the species barrier (human and pigs).

Among the 18 most abundantly used codons in EIVs, fourteen codons (AUU, CCA, ACA, GCA, UAU, UCA, CGA, CAU, CAA, AAU, AAA, GAU, GAA, GGA) were A/U-ended (A-ended: 9; U-ended: 5) and the remaining four (UUC, UUG, GUG, UGC) were G/C-ended codons in H3N8 subtype. It is interesting to note that H7N7 subtype utilized seventeen A/U-ended codons (CUU, AUA, GUA, CCA, ACA, GCA, UAU, UCA, CGA, UGU, CAU, CAA, AAU, AAA, GAU, GAA, GGA); one C-ended codon (UUC) and none of the preferred codon was G-ended (Table 1). However, there was no significant difference between both subtypes of EIVs with respect to the preference towards A/U ended codons (p<0.05). It is evident from RSCU analysis that EIV genomes exhibit higher codon usage bias towards A/U- compared to G/C-ended codons. Previously, such kind of analysis in different influenza viruses, notably H5N1 [22], H1N1 and H3N2 [25] also highlighted the preference towards A- or U-ended codons, as is consistent with the mutational biases found in previous studies [27–28].

**Fig 1:**
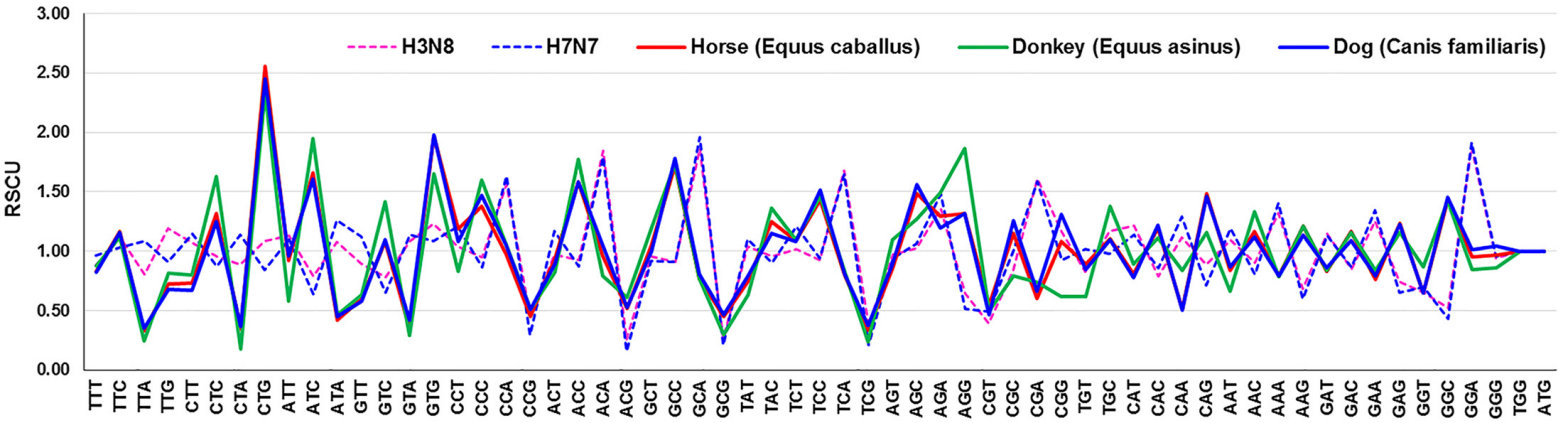
Comparative analysis of Relative Synonymous Codon Usage (RSCU) patterns of EIV subtypes (H3N8 and H7N7) with their clinical and accidental hosts.

Despite belonging to same *Equidae* (horse and donkey) family, we noticed close homology in RSCU patterns of horse and dog compared to donkey (Fig 1). The overall patterns of 59 synonymous codons usage were relatively consistent among these two subtypes of EIVs, indicating that the evolutionary processes of both H3N8 and H7N7 subtypes of EIVs, to some extent might be restricted by the synonymous codon usage pattern.

Furthermore, RSCU values were divided into three categories: (A) codons with RSCU values ≤ 0.6 (under-represented), (B) codons with RSCU values between 0.6 and 1.6 (unbiased- represented), and (C) codons with RSCU values ≥ 1.6 (over-represented) [23]. Analysis of over-and under- represented codons showed that RCSU values of the majority preferred and non-preferred codons fell between 0.6 and 1.6. It is quite interesting to note that over-represented codons are A-ended and mostly under-represented codons are C/G-ended (Table 1). We could not find a common single codon, which is over-represented in both EIVs and potential host species; instead for CGA (Arg), EIVs were over-represented and host species (duck, goose and chicken) were under-represented. The previous study emphasized that preferred Arg codons in H1N1, H1N1pdm and H3N2 were AGA and AGG, while CGN were under-represented [25]. In contrast, EIVs preferred codon for Arg is CGA, which is less frequently used in other potential host species and under-represented codons are AGG and CGU. Based on the nucleotide compositional and RSCU analysis, it is inferred that the usage of preferred codons might be influenced mostly by compositional constraints, which also accounts for the presence of mutational pressure.

### Significant Influence of Dinucleotide Frequencies in Determining the Codon Usage Bias

**Fig 2:**
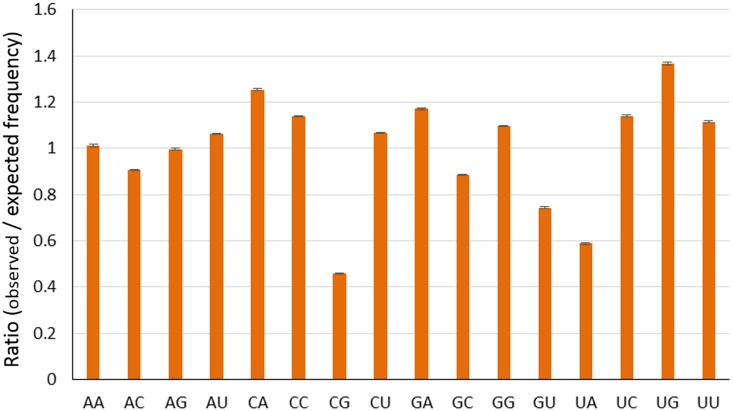
Relative dinucleotides frequencies among EIV strains.

Compositional constraints, reinforced by mutational biases, have been found to reflect forces on dinucleotides in the evolution of influenza viruses. The relative abundance of dinucleotides could also affect the pattern of codon usage in RNA viruses [28,47–48]. Therefore, we calculated the relative abundances of 16 dinucleotides from the complete coding genomic sequences of EIVs. The relative abundance of CpG (mean ± SD = 0.457 ± 0.002) and UpA (mean ± SD = 0.587 ± 0.004) showed a severe deviation from the normal and were under-represented (Fig 2). The RSCU values of the eight codons containing CpG (CCG, UCG, GCG, ACG, CGG, CGC, CGU, and CGA) and six codons containing UpA (UUA, CUA, AUA, GUA, UAU, and UAC) were analyzed to determine the possible effects of CpG and UpA representations on codon usage bias. In the case of CpG-containing codons, all the codons were under-represented (RSCU ≤ 1.6) and were not preferred codons for their respective amino acid, except for CGA (Arg). On the other hand, in case of UpA containing codons, all codons were not preferred codons for their respective amino acid, except for UAU (Tyr). Low relative abundance of CpG (CpG deficiency) was proposed to be related to the immune stimulatory properties of unmethylated CpG, which are recognized by the innate immune system of the host as a pathogen signature, and CpG sequences avoided in the evolution of human H1N1 have been found to stimulate the innate immune response [28,49–50]. Recognition of unmethylated CpG by Toll like receptor 9 (TLR9), a type of intracellular pattern recognition receptor, leads to activation of different immune response pathways [51]. The influenza A viruses, which have been replicating in the avian species over many generations are supposed to contain high CpG contents. Once they cross the species barrier (from birds to humans), they evolve by mimicking the host (human) genome [28]. For example, H5N1 avian and 1918 H1N1 viruses had much higher CpG content compared to human adapted influenza viruses, which suggest that they have been derived after adaptation in avian replicative machinery. In our study, we noticed a steady decrease in CpG frequency (0.0227 in 1963 to 0.0205 in 2013) in genome of EIVs suggestive of continuous evolution in the mammalian host (equines), which is accompanied by an increase in UpA dinucleotides frequencies. The decline in frequency of CpG containing oligomers in H3N8 had previously been observed [50]. Previous studies on RNA viruses, including avian influenza viruses, have shown marked CpG deficiency [28,47–48]. This CpG deficiency in RNA viral genomes is another selective pressure contributing in codon usage bias [52–54]. This pressure helps them in escaping the host antiviral immune response. In addition, usage of CpG in +ssRNA viruses is greatly influenced by hosts’ CpG usage [28, 47–48].

Low relative abundance of UpA has also been observed in RNA viruses [28]. As part of the vertebrate antiviral pathway, ribonuclease L degrades RNA molecule and activates apoptotic pathways [55]. RNase L preferentially targets UpA or UpU sites in West Nile Virus [56]. Recently, the role of RNase L in cleaving the influenza virus RNA has been decoded [57]. It is also assumed that UpA being the integral part in two out of three stop codons as well as in transcriptional regulatory motifs, is responsible for its deficiency [32,58]. Consequently, EIVs might get benefitted by UpA deficiency in three possible ways—(i) it might reduce the risk of nonsense mutations; (ii) it might minimize improper transcription; and finally (iii) it might minimize the chances of cleavage by RNase L.

The relative abundance of UpG (mean ± SD = 1.365 ± 0.007) and CpA (mean ± SD = 1.254 ± 0.003) dinucleotides also indicated a severe deviation from the normal and were over-represented compared with the rest of the 14 dinucleotides (Fig 2). Interestingly, all the five codons containing UpG (UUG, CUG, GUG, UGU and UGC) were under-represented (RSCU ≤ 1.6) and only three of them were the preferential codons (UUG, GUG, UGC) for their respective amino acids. Among the eight codons containing CpA (UCA, CCA, ACA, GCA, CAA, CAG, CAU and CAC), half of them (CCA, ACA, GCA and TCA) were over-represented (RSCU ≥ 1.6) except for CCA in H3N8 (RCSU = 1.56). A majority of them were preferential codons for their respective amino acids, based on RSCU analysis. The relative abundance of UpG and CpA in different organisms has been postulated as a consequence of the under-representation of CpG dinucleotides [59–60]. This analysis suggested that dinucleotide compositions play significant role in determining the codon usage patterns in EIVs. Given that the primary pressure is on CpG dinucleotides, which are avoided in the evolution of H3N8 in a manner which cannot be accounted for due to codon bias or amino acid sequence and which have been previously validated as immunogenic, selection pressure imposed by host antiviral immunity might also have a role in shaping the overall synonymous codon usage in EIVs.

### Codon Free Dinucleotide Frequency Difference and Selective Forces

To further reinforce that the observed dinucleotide changes are driving codon usage, rather than the other way around, we employed a set of methods described earlier [28,33]. First we calculated the expected number of dinucleotides, given a sequence amino acid usage and codon bias and compared it to the observed value. In Table 2, we show this difference for all 16 dinucleotides, and observed substantial differences in CpG and UpA dinucleotides in particular, along with the expected accompanying changes in CpA and UpG dinucleotides. These are the only dinucleotides with changes more than two standard deviations from their expected values.

**Table 2:** Average observed values for all 16 dinucleotides, along with the expected values when codon usage and amino acid sequence are accounted for. Here the Z-Score describes the difference between the expected and observed values divided by the standard deviation.

Dinucleotides	Expected Numbers	Observed Numbers	Differences	Z-Scores
AA	1601.3	1602.3	1.0285	0.043371
AC	840.74	819.21	-21.527	-1.2384
AG	1099.2	1100.1	0.96111	0.042927
AU	1114.1	1132.4	18.324	1.019
CA	1008	1132.6	124.58	6.2554
CC	557.87	585.52	27.646	1.4453
CG	437.4	287.98	-149.43	-9.9334
CU	650.69	648.51	-2.1816	-0.12113
GA	1257.1	1293.2	36.114	2.0762
GC	569.81	557.38	-12.429	-0.853
GG	852.12	843.3	-8.8229	-0.47842
GU	566.3	552.08	-14.22	-1.0243
UA	788.64	626.44	-162.2	-8.8864
UC	685.59	692.28	6.6927	0.34599
UG	856.88	1014.2	157.27	8.7255
UU	801.04	798.8	-2.2447	-0.10406

**Fig 3:**
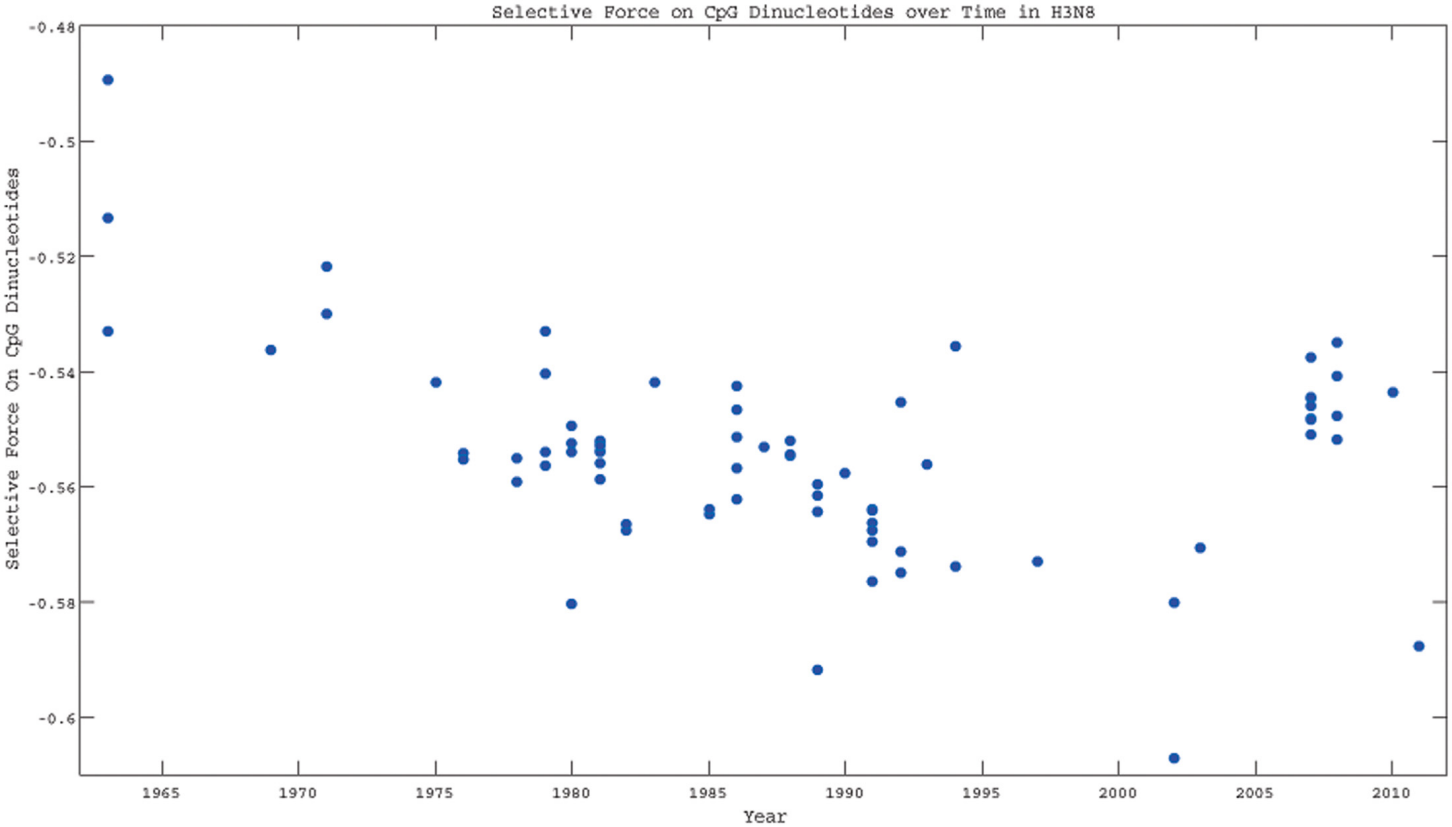
The selective force on the CpG dinucleotide as a function of time for H3N8 viruses. The force moves becomes larger in magnitude over time.

As a result, the changes in codon frequency are likely driven by these changes, rather than the other way around. A set of evolutionary methods for quantifying such changes have been described which calculates the selective force on a dinucleotide [33]. In this case, such a force in the cost in sequence entropy of changing the occurrence of a motif, once amino acid sequence and codon usage are accounted for. In Fig 3, we see that the force on CpG is driving H3N8 sequences further and further away from the value one would expect from amino acid sequence and codon usage, while the force on UpA is getting closer to its maximum entropy value over time (S1 Fig). As a result, we can conclude that the primary selective pressure is on the CpG dinucleotides, and that this pressure can drive codon usage patterns through mutational biases over time.

### Codon Usage Bias among EIVs

**Fig 4:**
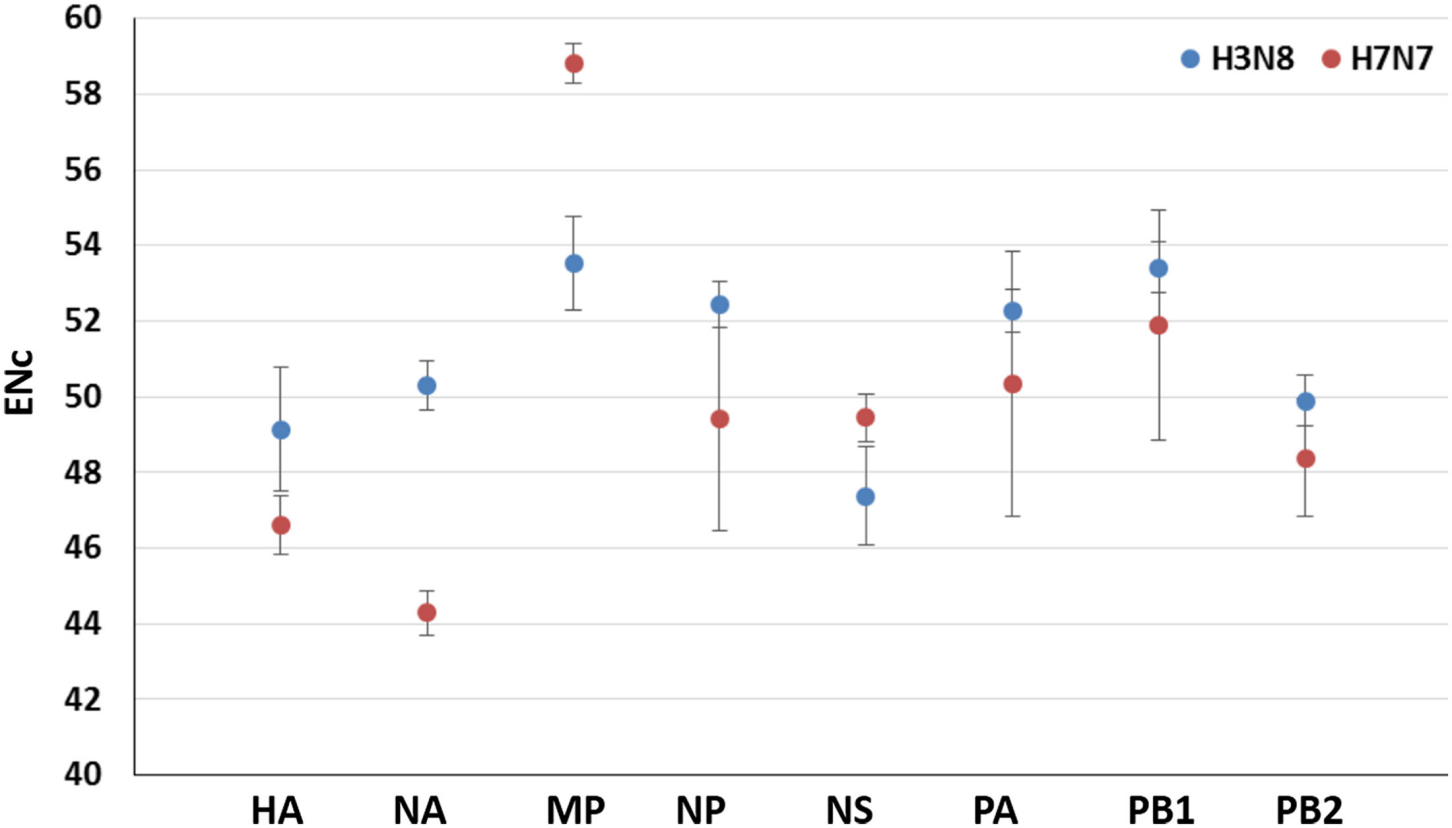
Comparative analysis of Effective Number of Codon (ENc) values in H3N8 and H7N7 subtypes.

To quantify the extent of variation in codon usage between two subtypes of EIVs, the ENc values were calculated [34]. The ENc values among EIVs genomes ranged from 47.7 to 53.72, with a mean of 52.09 and SD of 1.08. While analyzing the subtype-wise ENc, we detected statistically significant high ENc value of H3N8 subtype compared to H7N7 (t-test, p <0.0001). The overall lower ENc value in H7N7 subtype was predominantly due to very low ENc values (47.7–47.9) noticed in four H7N7 isolates (3 isolates from Prague and one from Lexington collected in the year 1956 and 1966, respectively). These four isolates also formed a monophylactic group when subjected to phylogenetic analysis (S2 Fig). Further, we noticed statistically significant differences in codon usage bias in all the gene segments of H3N8 compared to H7N7 subtype (p <0.0001) (Fig 4 and S3 Table). Overall, the mean ENc value (52.09) of 92 EIV strains indicates a relatively stable and conserved genomic composition. Our analysis signposts that codon usage bias in EIVs is slightly lower and might be mainly affected by nucleotide compositions. Different RNA viruses in previous studies on codon usage have also depicted lower codon usage bias, such as H5N1 Influenza virus (ENc = 50.91) [22]; H1N1pdm IAV (ENc = 52.5) [25]; Chikungunya Virus (ENc = 55.56) [60]; Foot-and-Mouth Disease Virus (ENc = 51.42) [61]; Equine Infectious Anemia Virus (ENc = 43.61) [62]; Bovine Viral Diarrhea Virus (ENc = 50.91) [63]; Classical Swine Fever Virus (ENc = 51.7) [64] and West Nile Virus (ENc = 53.81) [65]. However, we could locate the high codon usage bias in different plant viruses [66]; vertebrate DNA viruses such as Bovine Herpes Virus 1 (ENc = 36.83), Bovine Herpes Virus 5 (ENc = 32.55), Suid Herpes Virus (ENc = 29.96), some primate Herpes Viruses (ENc = 32.59 to 41.87) [67], Human Papillomavirus [68]; and RNA virus such as Hepatitis A Virus (ENc = 39.34) [69].

Furthermore, we compared the codon usage preferences of EIVs with those of different potential host species using Codon Adaptation Index (CAI). EIVs segment-wise and subtype-wise CAI values with respect to potential host species are provided in S4 Table. The Kruskal–Wallis test revealed that difference between the CAI values of H3N8 and H7N7 subtypes estimated in relation to equine codon usage reference set were statistically non-significant (p < 0.05).

**Fig 5:**
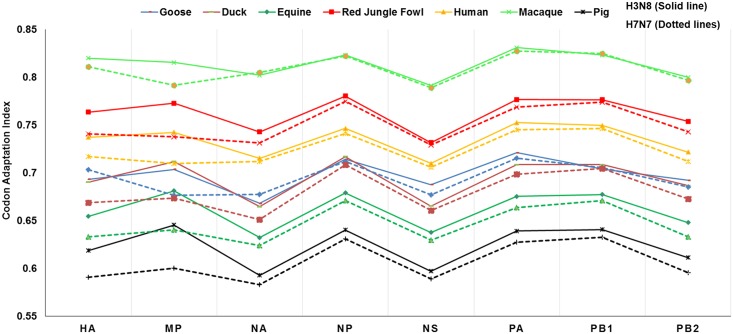
Codon usage preferences of EIV subtypes in relation to the codon usage of potential host species as estimated by Codon Adaptation Index (CAI).

We deliberately included macaque in our study to examine their codon usage pattern with respect to EIVs because of two reasons; (i) The macaque is a well-established model to study the host immune response against influenza viruses [70–72], (ii) Recent detection of anti-influenza nucleocapsid protein antibodies in non-human primate populations from different countries [73]. As evident from the Fig 5, we noticed a peculiar similarity in the pattern of codon usage in two subtypes of EIVs with respect to potential host species. It is surprising that Macaque showed the highest CAI value (0.813±0.003) followed by red jungle fowl (0.762±0.004), human (0.734±0.004), goose (0.697±0.004), duck (0.694±0.005), equine (0.660±0.005) and the lowest in pig (0.623±0.005) (S4 Table). However, Dunn's multiple comparison test revealed that codon usage preference of EIVs with equine was significantly different from that of red jungle fowl (p <0.01) and macaque (p <0.0001), while non-significant with goose, duck, pig, and human (p <0.05) (S5 Table). The aquatic birds are considered as natural reservoir for influenza viruses, thus, it is expected that codon usage preference of EIVs with respect to red jungle fowl is high. However, highest CAI values in the macaque is quite thought-provoking.

Additionally, we calculated CAI of H3N8 subtype with respect to other established study model for influenza A viruses- mice (0.716±0.003), and newly targeted species—canine (0.660±0.017). Remarkably, CAI of H3N8 viruses isolated from equines and canines were identical. Not only CAI, we found great similarity in the nucleotide composition, RSCU as well as ENc values of canine H3N8 with the currently circulating equine H3N8 viruses (Fig 1 and S6 Table). This clearly suggests that the equine H3N8 viruses might have crossed the species barrier and started infecting the canines. Earlier, phylogenetic analysis had revealed the similar findings, where influenza viruses isolated from canines in the early 2000s formed monophylactic group with equine H3N8 viruses [74]. Recently, it has been experimentally proved that H3N8 viruses infecting equines and canines do possess almost similar biological properties in terms of growth in different cell cultures, preference for α-2,3 linked sialic acid receptors for infections, levels of infection in tracheal explant cultures and HA cleavage efficiency [75]. However, the factors responsible for crossing the species barrier by influenza viruses still remain mysterious.

### Mutation pressure plays a key role in the codon usage bias of EIVs

**Fig 6:**
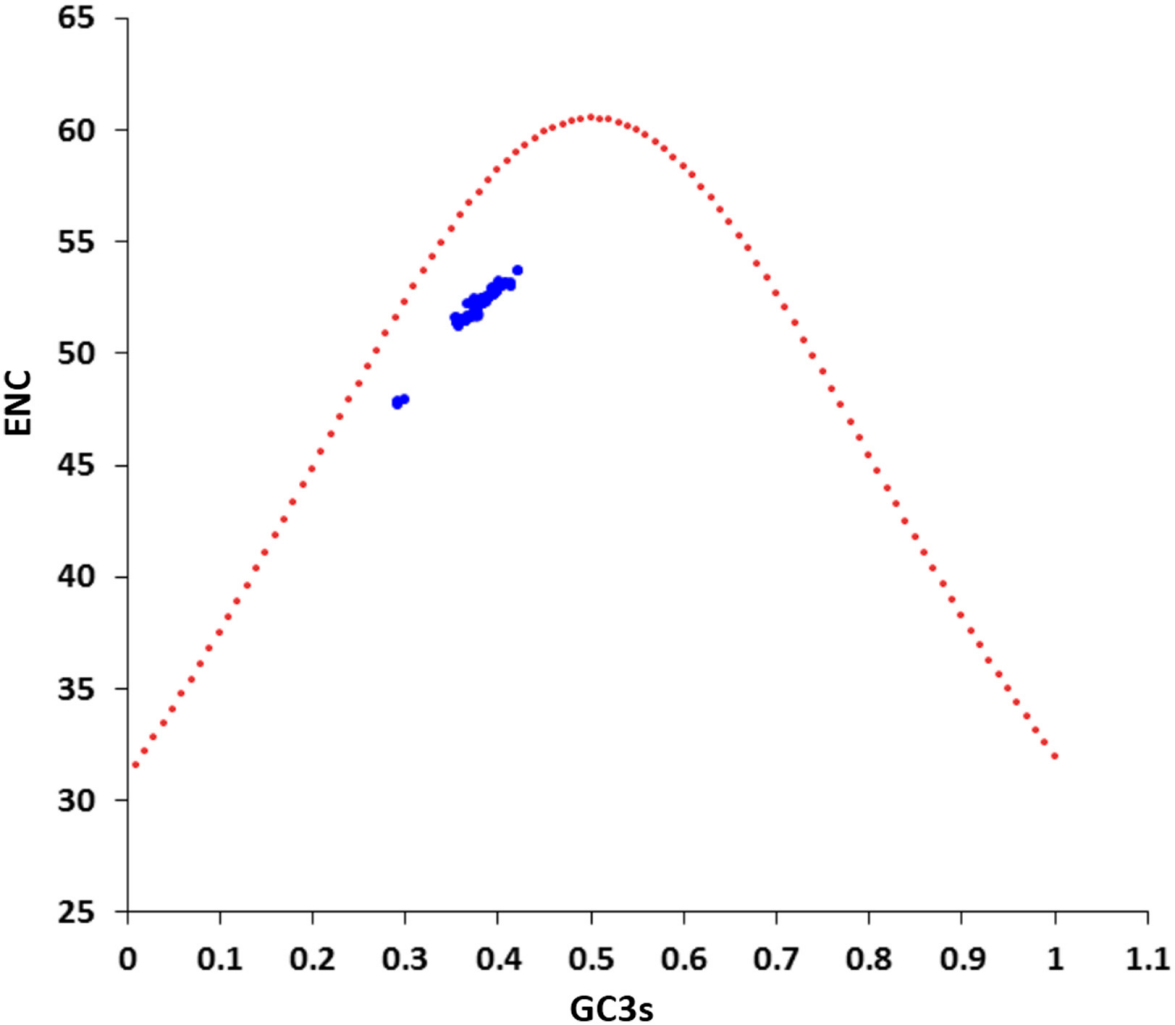
ENc-plot analysis (ENc plotted against GC3s). ENc denotes the effective number of codons, and GC3s denotes the GC content on the third synonymous codon position. The red dotted line represents the expected curve derived from the positions of strains when the codon usage was only determined by the GC3s composition.

To determine the role of nucleotide compositional constraint or mutational pressure on structuring the synonymous codon usage bias in EIV genomes, we performed ENc-plot (ENc values were plotted against the GC3s values). It has been suggested that if synonymous codon usage bias is constrained by only GC content on the third synonymous codon position (GC3s), then all points will lie exactly on the standard curve [34]. In Fig 6, all the points lie under the standard curve. It implies that the mutational pressure is not the sole factor in shaping the codon usage bias, but other factors, such as natural selection are likely to be involved in determining the selective constraints on the codon usage bias in EIV genomes. We also estimated the ratio of difference between the observed and the expected ENc values to expected ENc values for each strain of EIV. Frequency distribution of all the strains was within the short range of 0.07–0.09 suggesting that ENc values of all the strains are less than the expected ENc values. This is in accordance with the ENc-GC3s plot analysis, which further provided evidence that GC3s is behind the conditional mutation bias in EIVs.

**Fig 7:**
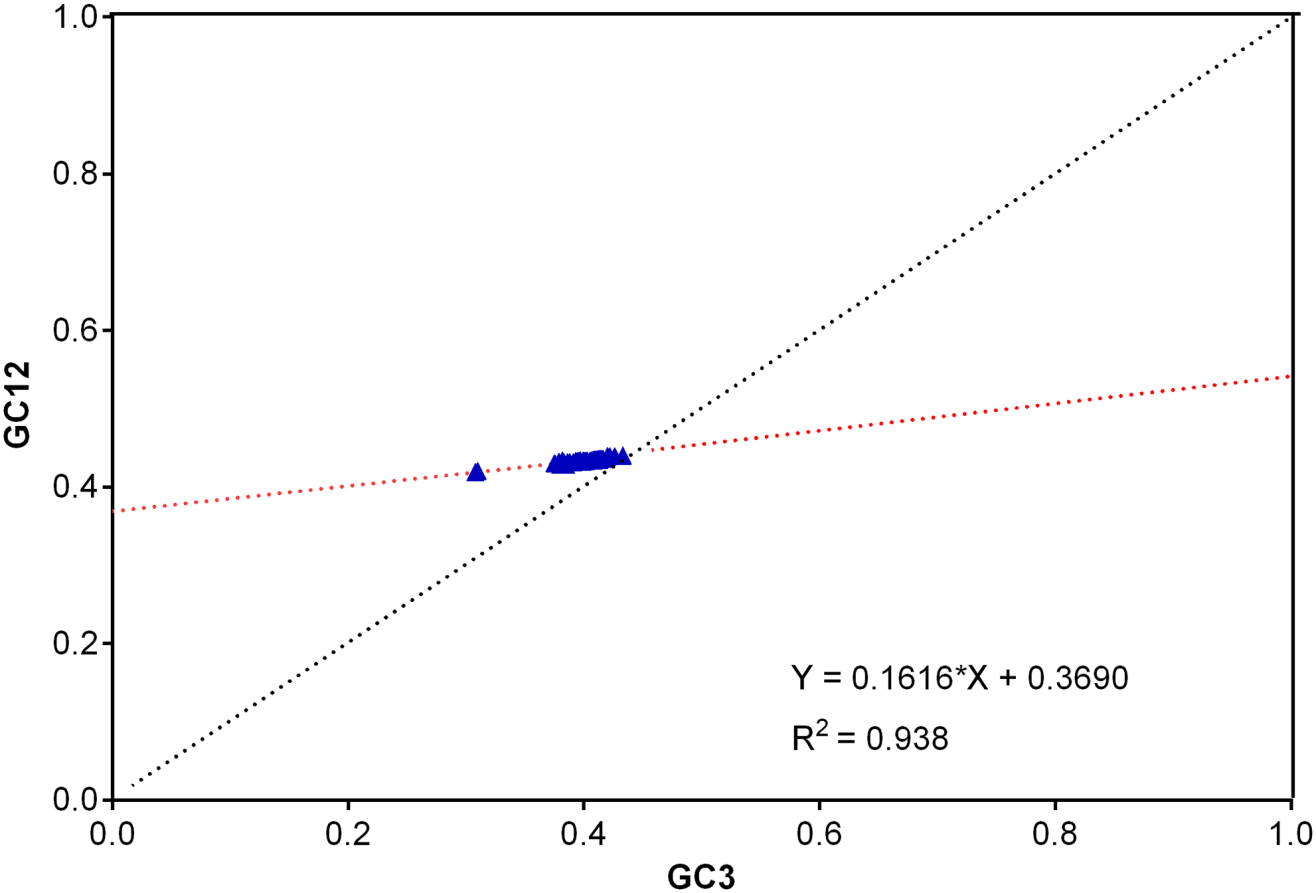
Neutrality plot analysis (GC12 vs GC3) for the entire coding sequences of EIVs. GC12 stands for the average value of GC contents at the first and second positions of the codons (GC1 and GC2), while GC3 refers to the GC contents at the third codon position. The red dotted line is the linear regression of GC12 against GC3, R^2^ = 0.938, P <0.001.

Further, we performed neutrality plot analysis to decipher the role of key determinant factors, i.e. natural selection and mutation pressure in shaping the codon usage bias. The neutrality plot revealed a narrow range of GC3 values (34.2% to 46.4%) distribution among EIV strains (Fig 7). The significantly high positive correlation between GC12 and GC3 (r = 0.953, p <0.0001) was noticed, indicating the presence of directional mutation pressure effect at all codon positions. Also, the slope of the regression line of the entire coding genomic sequences of EIVs was 0.1657. To decipher the degree to which mutation pressure plays role in different subtypes of EIVs, we performed regression analysis separately. We recorded different slopes for H3N8 (0.1852) and H7N7 (0.1404) subtypes. These results advocated that the effects of directional mutation pressure in H3N8 and H7N7 subtypes were 18.52% and 14.04%, respectively.

To know the extents by which different determinants were significantly influencing the codon usage bias in EIVs, we performed COA (described in detail elsewhere in the document). The COA analysis revealed that A3 or U3 (r = 0.977 and r = 0.861, respectively, p <0.001) showed statistically significant positive correlation with first major axis and negative correlation with G3 or C3 or GC3 (r = -0.993, -0.906, -0.995, respectively, p <0.001) (Table 3). In addition, the strains positions on axis 1 were strongly negatively correlated with the GC3s (r = -0.994, p <0.001) and ENc values (r = -0.968, p <0.001). Furthermore, Aromo and Gravy values were highly positively correlated with minor axis 2 (r = 0.614 and r = 0.514, respectively, p <0.01). This analysis suggests that mutational pressure or compositional constraints, and not the natural selection, play significant and a major role in overall shaping of codon usage bias in EIVs. Previously conducted similar studies also highlighted the significant role of mutational pressure in structuring the codon usage patterns in H1N1 pdm IAV [25], RNA and DNA viruses [48].

**Table 3:** Correlation analysis among different nucleotides compositions of EIV subtypes and the first two principal axes of COA.

**Variables**	**A**	**C**	**G**	**U**	**U3**	**C3**	**A3**	**G3**	**GC**	**AU**	**GC1**	**GC2**	**GC3**	**AU3**	**GC12**	**ENC**	**Axis1**
**A**																	
**C**	-0.915**																
**G**	-0.995**	0.904**															
**U**	0.891**	-0.976**	-0.889**														
**U3**	0.839**	-0.870**	-0.845**	0.925**													
**C3**	-0.887**	0.982**	0.877**	-0.970**	-0.898**												
**A3**	0.976**	-0.944**	-0.967**	0.905**	0.802**	-0.906**											
**G3**	-0.986**	0.924**	0.985**	-0.901**	-0.847**	0.890**	-0.982**										
**GC**	-0.993**	0.943**	0.991**	-0.922**	-0.856**	0.913**	-0.980**	0.989**									
**AU**	0.993**	-0.943**	-0.991**	0.922**	0.856**	-0.913**	0.980**	-0.989**	-1.000**								
**GC1**	-0.972**	0.918**	0.968**	-0.891**	-0.804**	0.878**	-0.969**	0.966**	0.976**	-0.976**							
**GC2**	-0.741**	0.641**	0.748**	-0.689**	-0.687**	0.627**	-0.670**	0.692**	0.724**	-0.724**	0.682**						
**GC3**	-0.983**	0.947**	0.979**	-0.922**	-0.863**	0.918**	-0.981**	0.994**	0.992**	-0.992**	0.962**	0.691**					
**AU3**	0.983**	-0.944**	-0.980**	0.920**	0.863**	-0.913**	0.982**	-0.995**	-0.991**	0.991**	-0.963**	-0.689**	-0.998**				
**GC12**	-0.978**	0.908**	0.980**	-0.892**	-0.824**	0.874**	-0.949**	0.954**	0.977**	-0.977**	0.976**	0.786**	0.954**	-0.953**			
**ENC**	-0.958**	0.936**	0.953**	-0.901**	-0.811**	0.897**	-0.981**	0.975**	0.966**	-0.966**	0.957**	0.642**	0.972**	-0.974**	0.928**		
**Axis1**	0.985**	-0.934**	-0.985**	0.914**	0.861**	-0.906**	0.977**	-0.993**	-0.990**	0.990**	-0.962**	-0.709**	-0.995**	0.994**	-0.959**	-0.968**	
**Axis2**	0.550**	-0.639**	-0.530**	0.572**	0.334*	-0.581**	0.653**	-0.574**	-0.577**	0.577**	-0.595**	-0.231	-0.580**	0.581**	-0.537**	-0.638**	0.564**

*p<0.001

**p<0.0001

### Bantam role of translational selection in codon usage bias

It has been proposed that the preferred codons are recognized by the most abundant isoacceptor tRNAs, which indicates the influence of translational selection on codon usage [76]. Since the translation is the main process in any virus life cycle, hence it is important to gain insight into the adaptation of EIV subtypes in the equine tRNAs pool. Consequently, we analyzed the most preferred codon families of EIV genomes with respect to host (equine) tRNAs pool. On comparing the tRNA anti-codon and synonymous codon families, except for Trp and Met, in seven (Tyr, His, Gln, Asn, Lys, Asp and Glu) out of nine amino acids, two-fold synonymous codon families were found to have ‘non-optimal codon–anticodon usage’ (most preferred codon in these families have their corresponding less frequent tRNA isotypes in equine cells). For example, the RSCU value of CAU (His) was higher than CAC, yet the wobble position of the most frequent tRNA-His anti-codon was G (Table 4). Rest two amino acids (Phe and Cys) were strongly preferred, i.e. ‘optimal codon–anticodon usage’. Likewise, ‘combined codon–anticodon usage’ was found in the rest of the synonymous codon families, where at least two synonymous codons RSCUs were distinctly higher than the others.

**Table 4:** Frequency of tRNA genes in equine cells for most preferentially used codons in EIVs.

Amino Acid	Most preferred codons in EIVs	tRNA isotypes in equine cells	Total count
Ala	GCA	AGC (27), GGC (0), CGC (8), **TGC (10)**	45
Gly	GGA	ACC (0), GCC (10), CCC (8), **TCC (5)**	23
Pro	CCA	AGG (10), GGG (0), CGG (3), **TGG (7)**	20
Thr	ACA	AGT (9), GGT (0), CGT (3), **TGT (7)**	19
Val	GTG (H3N8), GTA (H7N7)	AAC (12), GAC (3), **CAC (16), TAC (6)**	37
Ser	TCA	AGA (12), GGA (0), CGA (4), **TGA (4),** ACT (0), GCT (12)	32
Arg	CGA	ACG (10), GCG (1), CCG (4), **TCG (5),** CCT (7), TCT (6)	33
Leu	TTG (H3N8), CTT (H7N7)	**AAG (8),** GAG (0), CAG (3), TAG (5), **CAA (6),** TAA (4)	26
Phe	TTC	AAA (0), **GAA (13)**	13
Asn	AAT	**ATT (1),** GTT (21)	22
Lys	AAA	CTT (18), **TTT (15)**	33
Asp	GAT	**ATC (1),** GTC (10)	11
Glu	GAA	CTC (50), **TTC (11)**	61
His	CAT	**ATG (1),** GTG (12)	13
Gln	CAA	CTG (10), **TTG (6)**	16
Ile	ATT (H3N8), ATA (H7N7)	**AAT (20),** GAT (0), **TAT (4)**	24
Tyr	TAT	**ATA (1),** GTA (14)	15
Cys	TGC (H3N8), TGT (H7N7)	**ACA (0), GCA (26)**	26
Trp	TGG	CCA (7)	7
Met	ATG	CAT (23)	23

Note: Codons which are likely to be paired with respective anticodon are highlighted in bold.

On probing the codon-anticodon usage subtype-wise in EIVs, we detected differences in codon-anticodon usage in H3N8 and H7N7 subtypes at four positions (Table 4). It is interesting to note that most preferred codons in H3N8 subtype at these four codon-anticodon positions (Val, Leu, Ile, and Cys) corresponded with the most frequent tRNA isotypes of equine cells; whereas in H7N7 subtype corresponded with less frequent tRNA isotypes expect Leu. Overall, our findings suggested that codon usage preference of EIVs does not seem to well adapt to tRNA pool of equine cells. The similar findings have also been observed for HIV-1 [77] and H1N1pdm IAV in human cells [25]. The HIV-1, instead of being poorly adapted to tRNA pool of human cells, expressed well by inducing alterations in the tRNA pool (two types of tRNA pools at different viral replication cycle stage). The first kind of tRNA pool, where translation machinery is dedicated in translating host proteins (early genes are expressed at this stage), while in the second kind tRNA pool, tRNAs translating A-ending codons are selectively enriched in human infected cells (supporting the expression of late genes of HIV-1) [77]. It is not clear whether such mechanism of modulation of tRNA pool for enhancing the translation efficiency prevails in EIV or not, and needs to be addressed further in this aspect.

### Codon usage bias has a significant correlation to aromaticity and genome length

Correlation analysis was performed to assess the relationship between the codon usage bias and hydrophobicity or aromaticity or genome length (aa) in EIV genomes. It is evident from the Table 5 that the Gravy values have strong significant negative correlation with gene length (r = -0.470, p <0.001), while Aromo values showed significant negative correlation with GC3s and ENc (r = -0.219, p <0.05; r = -0.273, p <0.01, respectively).

**Table 5:** Correlation analysis among genome length (L_aa), GC, GRAVY, AROMO, GC3s, ENc and the first two principal axes of COA.

**Variables**	**L_aa**	**GC**	**AROMO**	**GRAVY**	**GC3s**	**ENc**	**Axis 1**
**L_aa**							
**GC**	0.239*						
**AROMO**	0.108	-0.218*					
**GRAVY**	-0.470**	-0.097	0.202				
**GC3s**	0.288**	0.991**	-0.219*	-0.130			
**ENc**	0.273**	0.966**	-0.273**	-0.160	0.971**		
**Axis1**	-0.273**	-0.990**	0.189	0.115	-0.994**	-0.968**	
**Axis2**	-0.212*	-0.575**	0.614**	0.514**	-0.585**	-0.638**	0.564**

**p <0.01

*p <0.05

The results indicated that the Aromo values were associated with the codon usage bias in EIV genomes. The data in Table 5 also revealed that the gene length was positively correlated with the ENc values (r = 0.273, p <0.01), signifying that both gene length and aromaticity have a high correlation to the codon usage bias. COA analysis (described elsewhere in the document) revealed a significant positive correlation of Aromo and Gravy values with only axis 2 (r = 0.614 and r = 0.514, respectively, p <0.01), implicating that these factors play minor role in overall codon usage bias in EIVs.

### Correspondence analysis

**Fig 8:**
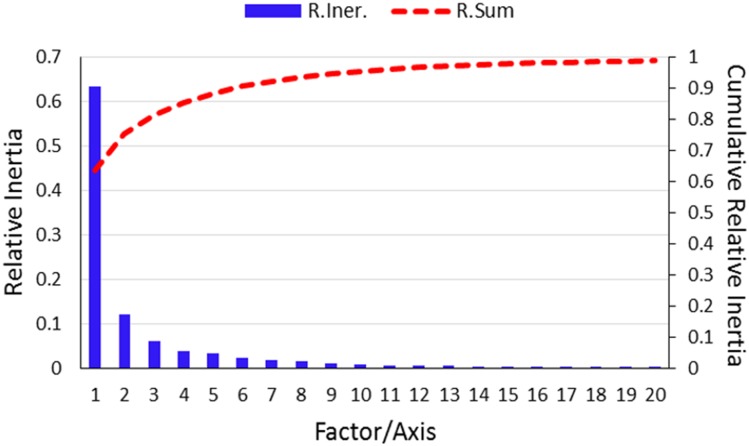
Contributions of the axes generated by Correspondence analysis (COA). The relative and cumulative inertia of the first 20 factors from a COA of the relative synonymous codon usage values. (R.Iner.—Relative Inertia, R.Sum—Relative sum or cumulative relative inertia).

Correspondence analysis (COA) is most commonly used multivariate statistical method to examine the variations in the RSCU values among the genes [41]. The significance of COA is reflected in its ability to distribute the different genes or strains in multidimensional space, including corresponding distribution of synonymous codons [78]. Therefore, COA of RSCU values was performed to understand the variations and trends of codon usage in EIVs. The first axis which explained 63.46% of the data inertia was the major factor in causing the variation, with each subsequent axes explained a declining amount of the variation (Fig 8). The first two axes accounted for nearly a third-fourth of total variation, hence our analysis was restricted to these two axes only. Moreover, multivariate correlation analysis was also performed to investigate the relationship between relative codon usage bias and nucleotide composition (Table 3).

**Fig 9:**
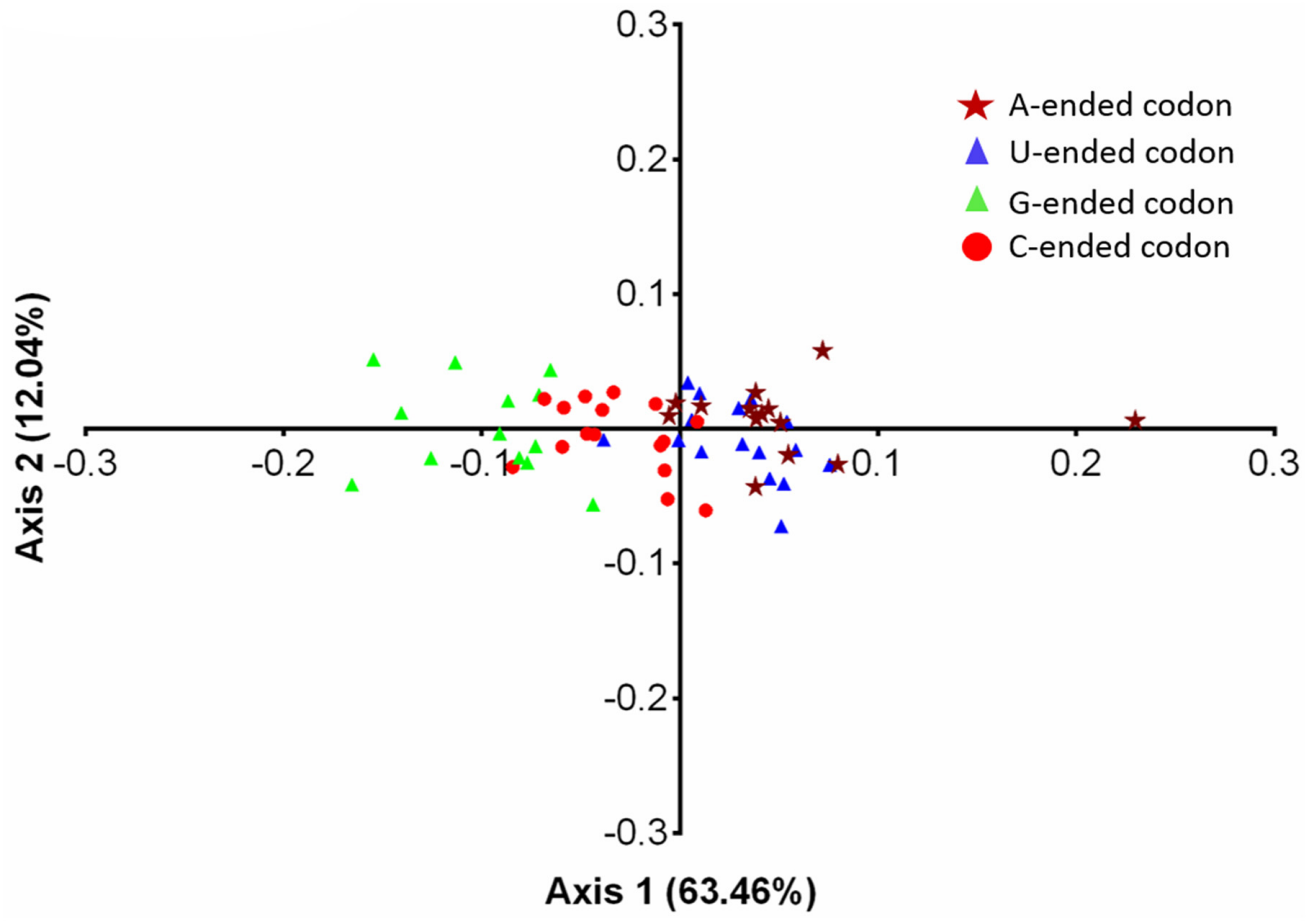
Correspondence analysis of the synonymous codon usage towards codons in EIVs. The analysis was based on the RSCU values of the 59 synonymous codons. The positions of each codon were described in the first two-main-dimensional coordinates. Different base-ended codons were marked in the figure, where the brown star, blue triangle, green triangle, and red circle refer to codons ending with A, U, G, and C, respectively.

**Fig 10:**
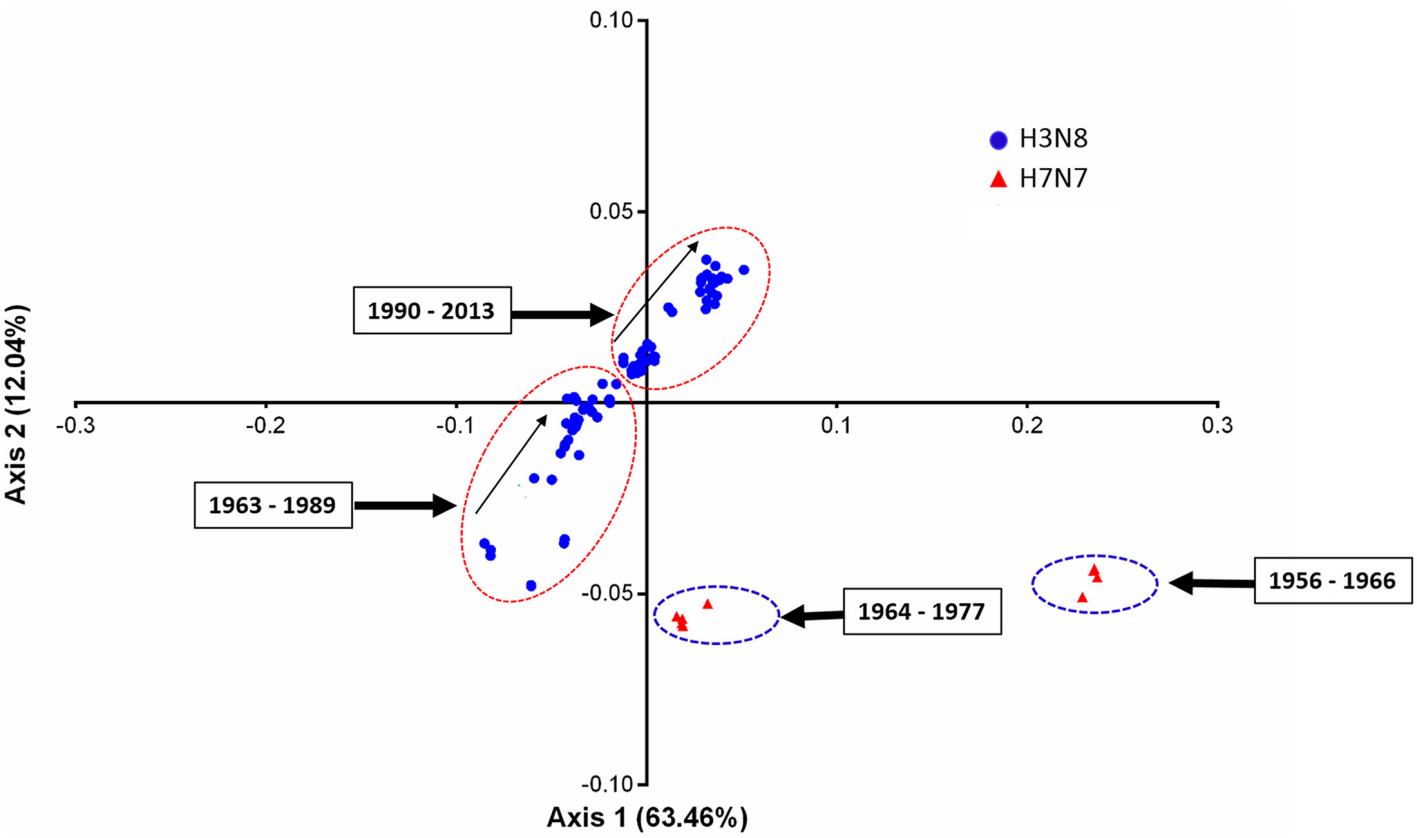
Correspondence analysis of the synonymous codon usage in two subtypes of EIVs. The analysis was based on the RSCU values of the 59 synonymous codons. The positions of each EIV strain were described in the first two-main-dimensional coordinates. Different subtypes of EIVs were marked in the figure, where the blue circle and red triangle refer to H3N8 and H7N7 subtypes, respectively.

When EIVs codons were allowed to get sorted based on the RSCU values across the two major axes of COA, the most extreme values were occupied by rarely used codons, and almost all of them contained the dinucleotides primarily CpG and UpA (Fig 9). The rarely used codons on axis 1 were UUA, UCG, CUG, CCG, ACG and GCG, and no such extreme values (rarely used codons) were observed on axis 2. The distribution of EIV strains based on the RSCU values on the first and second axes is shown in Fig 10. In general, four clusters were formed—two of which belong to H7N7 subtype. Of particular interest was the fact that the strains were separated along the first axis based on their GC contents, i.e. right side of the first axis occupied by the strains having GC <43%, while the left side by the strains having GC >43%. A steady increment in U and decline in GC content in RNA viruses over the course of time has been linked to their adaptation and evolution in mammalian hosts [27–28,79]. Furthermore, we analyzed the trends in nucleotide compositions of H3N8 and H7N7 subtypes of EIVs over the period of time (1963 to 2013). Interestingly, during the course of evolution, H3N8 subtype gradually reduced GC content over the time period of study, which is also reflected in the patterns followed by the positions of individual strain on first two major axes (Fig 10).

The avian-like H3N8 strain in the equine population emerged in 1989 in China, which is believed to have jumped directly from avian to the equine species without any reassortment [80]. This strain formed an outlier being having a completely different nucleotide compositions compared to the circulating equine H3N8 subtype of that time (S3 Fig) and also formed an outgroup in dendrogram (S2 Fig). Further, sudden fluctuations in GC content of H7N7 subtype isolated at different time intervals were noticed (S4 Fig). Previous study suggested that H7N7 subtype isolated from 1973 to 1977 were reassortants carrying H3N8 internal genes except M gene, and these reassortment events had occurred between 1964 and 1973 [81]. We came across the similar findings when we analyzed the nucleotide compositions of all gene segments of H7N7 isolates (S7 Table). The EIV H7N7 isolate (A/equine/Detroit/3/1964/H7N7) had GC content similarity to H3N8 viruses of that time for all the gene segments except HA and NA, while A/equine/Lexington/1/1966/H7N7 isolate possessed all gene segments having GC content similarity to H7N7 viruses. The H7N7 viruses isolated after 1975 possessed GC content similarity to H3N8 except HA and NA genes. These findings are in accordance with earlier findings and signify the evolution of H7N7 in conjunction with H3N8 subtype up to a certain time period.

RNA viruses do possess an error prone replicative machinery which allows them to form mutants cloud within a cell [82,83]. This mutants cloud preserves the identity of the viruses (do not allow them to become extinct) despite having high mutation rates. However, such mutants cloud was also supposed to form by H7N7 viruses to prevent their extinction. The possible explanations for disappearance of H7N7 subtype from equine population, as elucidated from our study could be due to (i) high codon usage bias in all gene segments except M and NS genes, (ii) lower directional mutation pressure, and (iii) lower adaptability to tRNA pool of equine cells compared to H3N8 subtype. In addition, widespread vaccination coverage around 1980s (probable ease in inducing protective antibody titer against H7N7 compared to H3N8), hetero-subtypic reassortment, and lower fitness of avian-like H7N7 genome in equine population are some other possible explanations suggested earlier [81].

## Conclusions

This study conclusively demonstrates that genome-wide codon usage patterns in EIVs are deciphered by the interplay of different determinant factors like mutation pressure, natural selection, Aromo values, genome length and undefined factors, though their dynamics may be complex. The codon usage bias in EIVs was weaker, and governed mainly by the mutation pressure or the nucleotide compositional constraints. These constraints are likely driven by the forces on dinucleotides and change over time, possibly due to evolution in a sequence specific manner under host innate immune pressure. The possible explanations for disappearance of H7N7 subtype might be associated with their comparatively higher codon usage bias, low mutation pressure and very less adaptation to tRNA pool of equine cells. The typical trends in codon usage as revealed by correspondence analysis allowed the differentiation of different EIV subtypes by forming the clusters. The variations in nucleotide compositions identified during the course of evolution suggest the co-evolution of both EIV subtypes. Hence, the findings of the present study aid in understanding the underlying factors involved in evolution of EIVs and fitness towards their hosts.

## Supporting Information

S1 Fig The selective force on the UpA dinucleotide as a function of time for H3N8. The force moves becomes smaller in magnitude over time. (TIF)

S2 Fig Phylogenetic analysis of whole genome sequences of all Equine Influenza virus isolates used in the study. (TIF)

S3 Fig Trends in different codon usage bias parameters in H3N8 viruses over the period of time (1963–2013). (TIF)

S4 Fig Trends in different codon usage bias parameters in H7N7 viruses over the period of time (1956–1977). (TIF)

S1 Table Details of Equine Influenza virus (EIV) strains of equid origin used in the study. (DOCX)

S2 Table Nucleotide compositional analysis of EIV genomes. (DOCX)

S3 Table Comparative analysis of ENc values of H3N8 and H7N7 subtypes of EIVs. (DOCX)

S4 Table Codon Adaptation Index (CAI) (both segment-wise and subtype-wise) of EIVs with respect to their potential hosts. (DOCX)

S5 Table Statistical analysis (ANOVA) of CAI values among different host species. (DOCX)

S6 Table Comparative nucleotide compositional analysis of H3N8 viruses originated from horse and dog. (DOCX)

S7 Table Trends in GC compositions in all gene segments of H7N7 viruses over the period of time (1956–1977). The sudden changes in GC contents in respective gene segments due to reassortment are highlighted.
(DOCX)
