# Supplementary figures and images for: Revelation of Influencing Factors in Overall Codon Usage Bias of Equine Influenza Viruses
Source: PLoS One. 2016 Apr 27;11(4):e0154376. doi: 10.1371/journal.pone.0154376 (PMC4847779; doi:10.1371/journal.pone.0154376)

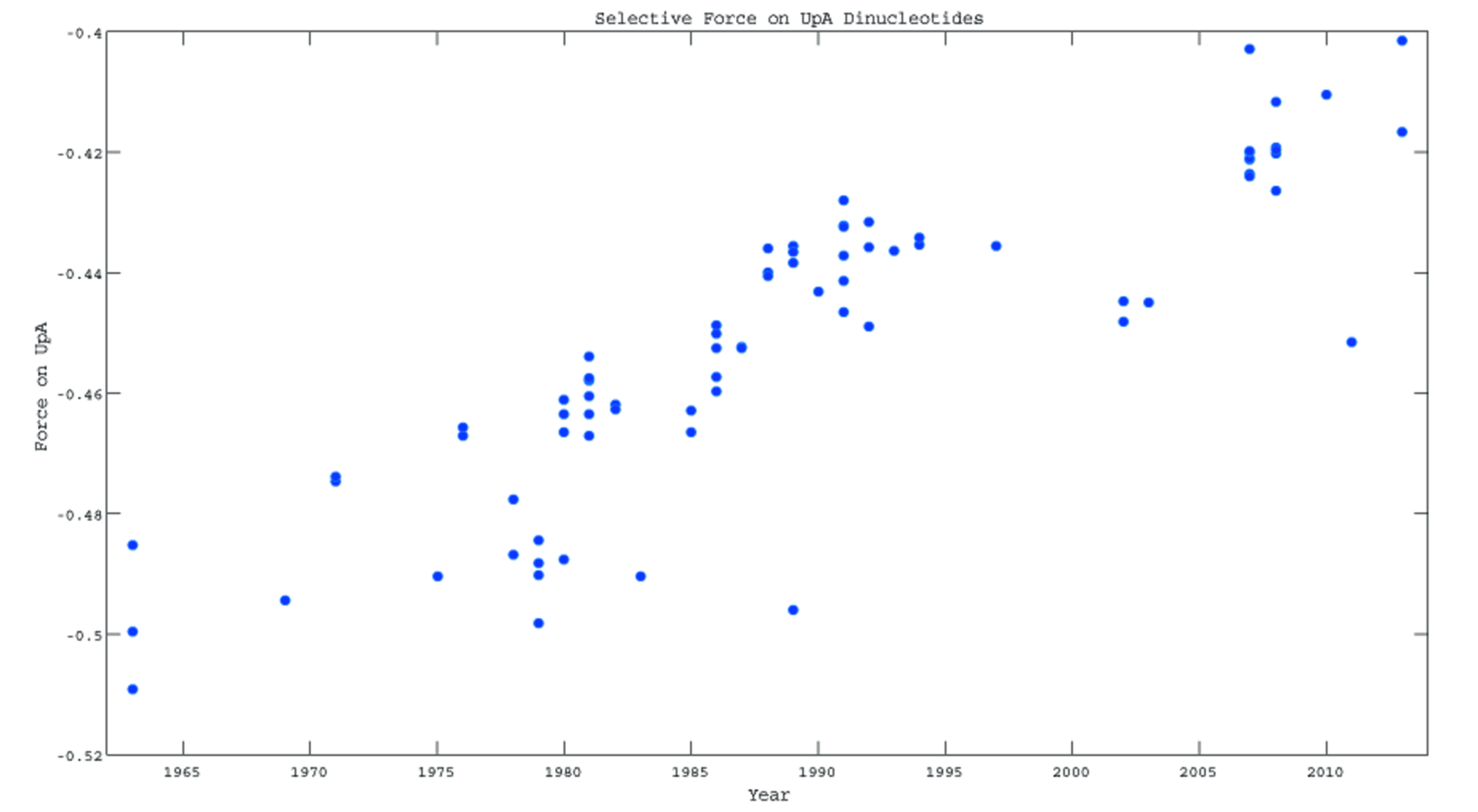

Supplement: S1 Fig — (TIF) [file pone.0154376.s001.tif]

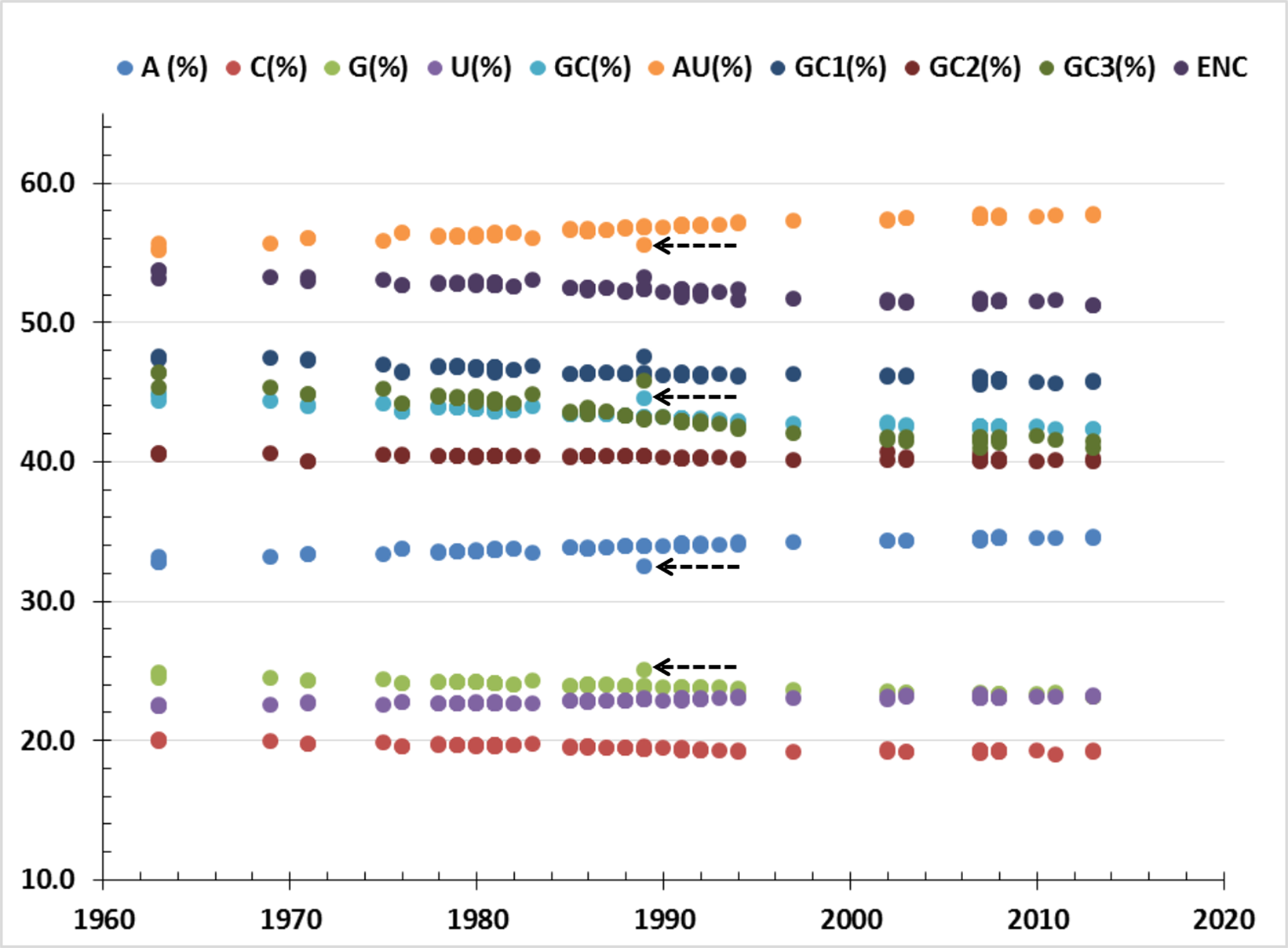

Supplement: S3 Fig — (TIF) [file pone.0154376.s003.tif]

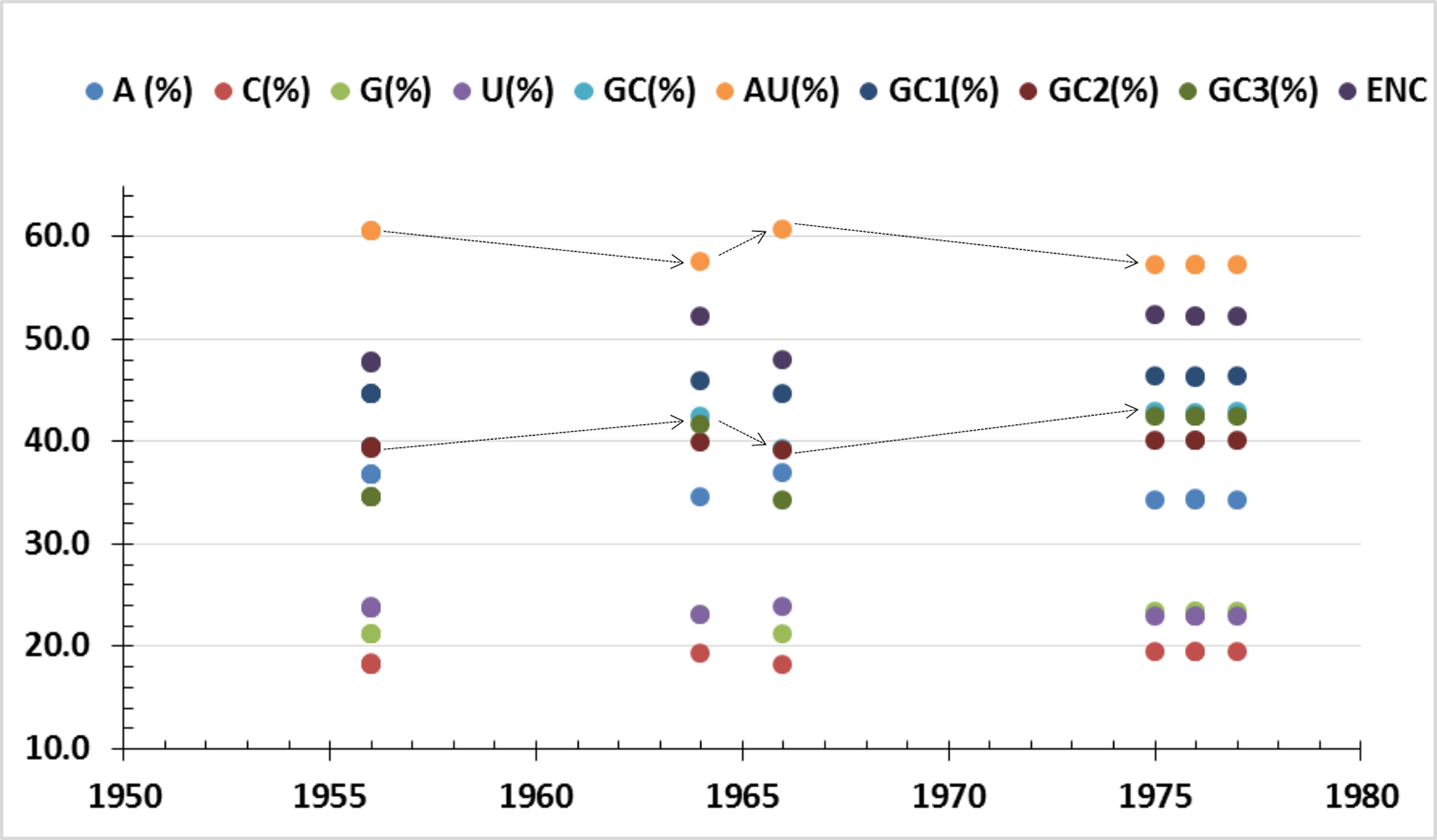

Supplement: S4 Fig — (TIF) [file pone.0154376.s004.tif]
