# Supplementary material for: Revelation of Influencing Factors in Overall Codon Usage Bias of Equine Influenza Viruses
Source: PLoS One. 2016 Apr 27;11(4):e0154376. doi: 10.1371/journal.pone.0154376 (PMC4847779; doi:10.1371/journal.pone.0154376)
Supplement: S1 Table — (DOCX) [file pone.0154376.s005.docx]

**S1 Table. Details of Equine Influenza virus (EIV) strains of equid origin used in the study.**

| **S. No.** | **Equine Influenza viruses used in this study** | **Country** | **Host** | | | **Year of isolation** | | **Subtype** | | **Segments** | | **Accession No.** | |
| --- | --- | --- | --- | --- | --- | --- | --- | --- | --- | --- | --- | --- | --- |
|  | A/equine/Heilongjiang/SS1/2013/H3N8 | China | | Equine | 2013 | | H3N8 | | PB2 | | KC986396 | |  |
|  | A/equine/Heilongjiang/SS1/2013/H3N8 | China | | Equine | 2013 | | H3N8 | | PB1 | | KC986397 | |  |
|  | A/equine/Heilongjiang/SS1/2013/H3N8 | China | | Equine | 2013 | | H3N8 | | PA | | KC986395 | |  |
|  | A/equine/Heilongjiang/SS1/2013/H3N8 | China | | Equine | 2013 | | H3N8 | | HA | | KC986390 | |  |
|  | A/equine/Heilongjiang/SS1/2013/H3N8 | China | | Equine | 2013 | | H3N8 | | NP | | KC986393 | |  |
|  | A/equine/Heilongjiang/SS1/2013/H3N8 | China | | Equine | 2013 | | H3N8 | | NA | | KC986392 | |  |
|  | A/equine/Heilongjiang/SS1/2013/H3N8 | China | | Equine | 2013 | | H3N8 | | MP | | KC986391 | |  |
|  | A/equine/Heilongjiang/SS1/2013/H3N8 | China | | Equine | 2013 | | H3N8 | | NS | | KC986394 | |  |
|  | A/equine/Xuzhou/01/2013/H3N8 | China | | Equine | 2013 | | H3N8 | | PB2 | | KF806992 | |  |
|  | A/equine/Xuzhou/01/2013/H3N8 | China | | Equine | 2013 | | H3N8 | | PB1 | | KF806991 | |  |
|  | A/equine/Xuzhou/01/2013/H3N8 | China | | Equine | 2013 | | H3N8 | | PA | | KF806990 | |  |
|  | A/equine/Xuzhou/01/2013/H3N8 | China | | Equine | 2013 | | H3N8 | | HA | | KF806985 | |  |
|  | A/equine/Xuzhou/01/2013/H3N8 | China | | Equine | 2013 | | H3N8 | | NP | | KF806988 | |  |
|  | A/equine/Xuzhou/01/2013/H3N8 | China | | Equine | 2013 | | H3N8 | | NA | | KF806987 | |  |
|  | A/equine/Xuzhou/01/2013/H3N8 | China | | Equine | 2013 | | H3N8 | | MP | | KF806986 | |  |
|  | A/equine/Xuzhou/01/2013/H3N8 | China | | Equine | 2013 | | H3N8 | | NS | | KF806989 | |  |
|  | A/equine/Kyonggi/SA1/2011/H3N8 | South Korea | | Equine | 2011 | | H3N8 | | PB2 | | JX844143 | |  |
|  | A/equine/Kyonggi/SA1/2011/H3N8 | South Korea | | Equine | 2011 | | H3N8 | | PB1 | | JX844144 | |  |
|  | A/equine/Kyonggi/SA1/2011/H3N8 | South Korea | | Equine | 2011 | | H3N8 | | PA | | JX844145 | |  |
|  | A/equine/Kyonggi/SA1/2011/H3N8 | South Korea | | Equine | 2011 | | H3N8 | | HA | | JX844146 | |  |
|  | A/equine/Kyonggi/SA1/2011/H3N8 | South Korea | | Equine | 2011 | | H3N8 | | NP | | JX844147 | |  |
|  | A/equine/Kyonggi/SA1/2011/H3N8 | South Korea | | Equine | 2011 | | H3N8 | | NA | | JX844148 | |  |
|  | A/equine/Kyonggi/SA1/2011/H3N8 | South Korea | | Equine | 2011 | | H3N8 | | MP | | JX844149 | |  |
|  | A/equine/Kyonggi/SA1/2011/H3N8 | South Korea | | Equine | 2011 | | H3N8 | | NS | | JX844150 | |  |
|  | A/equine/Heilongjiang/1/2010/H3N8 | China | | Equine | 2010 | | H3N8 | | PB2 | | KF309031 | |  |
|  | A/equine/Heilongjiang/1/2010/H3N8 | China | | Equine | 2010 | | H3N8 | | PB1 | | KF309032 | |  |
|  | A/equine/Heilongjiang/1/2010/H3N8 | China | | Equine | 2010 | | H3N8 | | PA | | KF309033 | |  |
|  | A/equine/Heilongjiang/1/2010/H3N8 | China | | Equine | 2010 | | H3N8 | | HA | | JQ265982 | |  |
|  | A/equine/Heilongjiang/1/2010/H3N8 | China | | Equine | 2010 | | H3N8 | | NP | | KF309034 | |  |
|  | A/equine/Heilongjiang/1/2010/H3N8 | China | | Equine | 2010 | | H3N8 | | NA | | KF309035 | |  |
|  | A/equine/Heilongjiang/1/2010/H3N8 | China | | Equine | 2010 | | H3N8 | | MP | | KF309036 | |  |
|  | A/equine/Heilongjiang/1/2010/H3N8 | China | | Equine | 2010 | | H3N8 | | NS | | KF309037 | |  |
|  | A/equine/Gansu/7/2008/H3N8 | China | | Equine | 2008 | | H3N8 | | PB2 | | EU794492 | |  |
|  | A/equine/Gansu/7/2008/H3N8 | China | | Equine | 2008 | | H3N8 | | PB1 | | EU794493 | |  |
|  | A/equine/Gansu/7/2008/H3N8 | China | | Equine | 2008 | | H3N8 | | PA | | EU794494 | |  |
|  | A/equine/Gansu/7/2008/H3N8 | China | | Equine | 2008 | | H3N8 | | HA | | EU794495 | |  |
|  | A/equine/Gansu/7/2008/H3N8 | China | | Equine | 2008 | | H3N8 | | NP | | EU794496 | |  |
|  | A/equine/Gansu/7/2008/H3N8 | China | | Equine | 2008 | | H3N8 | | NA | | EU794497 | |  |
|  | A/equine/Gansu/7/2008/H3N8 | China | | Equine | 2008 | | H3N8 | | MP | | EU794498 | |  |
|  | A/equine/Gansu/7/2008/H3N8 | China | | Equine | 2008 | | H3N8 | | NS | | EU794499 | |  |
|  | A/equine/Guangxi/1/2008/H3N8 | China | | Equine | 2008 | | H3N8 | | PB2 | | KP693697 | |  |
|  | A/equine/Guangxi/1/2008/H3N8 | China | | Equine | 2008 | | H3N8 | | PB1 | | KP693698 | |  |
|  | A/equine/Guangxi/1/2008/H3N8 | China | | Equine | 2008 | | H3N8 | | PA | | KP693699 | |  |
|  | A/equine/Guangxi/1/2008/H3N8 | China | | Equine | 2008 | | H3N8 | | HA | | KP693700 | |  |
|  | A/equine/Guangxi/1/2008/H3N8 | China | | Equine | 2008 | | H3N8 | | NP | | KP693701 | |  |
|  | A/equine/Guangxi/1/2008/H3N8 | China | | Equine | 2008 | | H3N8 | | NA | | KP693702 | |  |
|  | A/equine/Guangxi/1/2008/H3N8 | China | | Equine | 2008 | | H3N8 | | MP | | KP693703 | |  |
|  | A/equine/Guangxi/1/2008/H3N8 | China | | Equine | 2008 | | H3N8 | | NS | | KP693704 | |  |
|  | A/equine/Heilongjiang/10/2008/H3N8 | China | | Equine | 2008 | | H3N8 | | PB2 | | EU794508 | |  |
|  | A/equine/Heilongjiang/10/2008/H3N8 | China | | Equine | 2008 | | H3N8 | | PB1 | | EU794509 | |  |
|  | A/equine/Heilongjiang/10/2008/H3N8 | China | | Equine | 2008 | | H3N8 | | PA | | EU794510 | |  |
|  | A/equine/Heilongjiang/10/2008/H3N8 | China | | Equine | 2008 | | H3N8 | | HA | | EU794511 | |  |
|  | A/equine/Heilongjiang/10/2008/H3N8 | China | | Equine | 2008 | | H3N8 | | NP | | EU794512 | |  |
|  | A/equine/Heilongjiang/10/2008/H3N8 | China | | Equine | 2008 | | H3N8 | | NA | | EU794513 | |  |
|  | A/equine/Heilongjiang/10/2008/H3N8 | China | | Equine | 2008 | | H3N8 | | MP | | EU794514 | |  |
|  | A/equine/Heilongjiang/10/2008/H3N8 | China | | Equine | 2008 | | H3N8 | | NS | | EU794515 | |  |
|  | A/equine/Inner Mongolia/8/2008/H3N8 | China | | Equine | 2008 | | H3N8 | | PB2 | | EU794524 | |  |
|  | A/equine/Inner Mongolia/8/2008/H3N8 | China | | Equine | 2008 | | H3N8 | | PB1 | | EU794525 | |  |
|  | A/equine/Inner Mongolia/8/2008/H3N8 | China | | Equine | 2008 | | H3N8 | | PA | | EU794526 | |  |
|  | A/equine/Inner Mongolia/8/2008/H3N8 | China | | Equine | 2008 | | H3N8 | | HA | | EU794527 | |  |
|  | A/equine/Inner Mongolia/8/2008/H3N8 | China | | Equine | 2008 | | H3N8 | | NP | | EU794528 | |  |
|  | A/equine/Inner Mongolia/8/2008/H3N8 | China | | Equine | 2008 | | H3N8 | | NA | | EU794529 | |  |
|  | A/equine/Inner Mongolia/8/2008/H3N8 | China | | Equine | 2008 | | H3N8 | | MP | | EU794530 | |  |
|  | A/equine/Inner Mongolia/8/2008/H3N8 | China | | Equine | 2008 | | H3N8 | | NS | | EU794531 | |  |
|  | A/equine/Liaoning/9/2008/H3N8 | China | | Equine | 2008 | | H3N8 | | PB2 | | EU794516 | |  |
|  | A/equine/Liaoning/9/2008/H3N8 | China | | Equine | 2008 | | H3N8 | | PB1 | | EU794517 | |  |
|  | A/equine/Liaoning/9/2008/H3N8 | China | | Equine | 2008 | | H3N8 | | PA | | EU794518 | |  |
|  | A/equine/Liaoning/9/2008/H3N8 | China | | Equine | 2008 | | H3N8 | | HA | | EU794519 | |  |
|  | A/equine/Liaoning/9/2008/H3N8 | China | | Equine | 2008 | | H3N8 | | NP | | EU794520 | |  |
|  | A/equine/Liaoning/9/2008/H3N8 | China | | Equine | 2008 | | H3N8 | | NA | | EU794521 | |  |
|  | A/equine/Liaoning/9/2008/H3N8 | China | | Equine | 2008 | | H3N8 | | MP | | EU794522 | |  |
|  | A/equine/Liaoning/9/2008/H3N8 | China | | Equine | 2008 | | H3N8 | | NS | | EU794523 | |  |
|  | A/donkey/Xinjiang/5/2007/H3N8 | China | | Equine | 2007 | | H3N8 | | PB2 | | EU794572 | |  |
|  | A/donkey/Xinjiang/5/2007/H3N8 | China | | Equine | 2007 | | H3N8 | | PB1 | | EU794573 | |  |
|  | A/donkey/Xinjiang/5/2007/H3N8 | China | | Equine | 2007 | | H3N8 | | PA | | EU794574 | |  |
|  | A/donkey/Xinjiang/5/2007/H3N8 | China | | Equine | 2007 | | H3N8 | | HA | | EU794575 | |  |
|  | A/donkey/Xinjiang/5/2007/H3N8 | China | | Equine | 2007 | | H3N8 | | NP | | EU794576 | |  |
|  | A/donkey/Xinjiang/5/2007/H3N8 | China | | Equine | 2007 | | H3N8 | | NA | | EU794577 | |  |
|  | A/donkey/Xinjiang/5/2007/H3N8 | China | | Equine | 2007 | | H3N8 | | MP | | EU794578 | |  |
|  | A/donkey/Xinjiang/5/2007/H3N8 | China | | Equine | 2007 | | H3N8 | | NS | | EU794579 | |  |
|  | A/equine/Huabei/1/2007/H3N8 | China | | Equine | 2007 | | H3N8 | | PB2 | | GU571147 | |  |
|  | A/equine/Huabei/1/2007/H3N8 | China | | Equine | 2007 | | H3N8 | | PB1 | | GU571148 | |  |
|  | A/equine/Huabei/1/2007/H3N8 | China | | Equine | 2007 | | H3N8 | | PA | | GU571149 | |  |
|  | A/equine/Huabei/1/2007/H3N8 | China | | Equine | 2007 | | H3N8 | | HA | | GU571144 | |  |
|  | A/equine/Huabei/1/2007/H3N8 | China | | Equine | 2007 | | H3N8 | | NP | | GU571146 | |  |
|  | A/equine/Huabei/1/2007/H3N8 | China | | Equine | 2007 | | H3N8 | | NA | | GU571145 | |  |
|  | A/equine/Huabei/1/2007/H3N8 | China | | Equine | 2007 | | H3N8 | | MP | | GU571150 | |  |
|  | A/equine/Huabei/1/2007/H3N8 | China | | Equine | 2007 | | H3N8 | | NS | | GU571151 | |  |
|  | A/equine/Richmond/1/2007/H3N8 | United Kingdom | | Equine | 2007 | | H3N8 | | PB2 | | KF559332 | |  |
|  | A/equine/Richmond/1/2007/H3N8 | United Kingdom | | Equine | 2007 | | H3N8 | | PB1 | | KF559333 | |  |
|  | A/equine/Richmond/1/2007/H3N8 | United Kingdom | | Equine | 2007 | | H3N8 | | PA | | KF559334 | |  |
|  | A/equine/Richmond/1/2007/H3N8 | United Kingdom | | Equine | 2007 | | H3N8 | | HA | | FJ195395 | |  |
|  | A/equine/Richmond/1/2007/H3N8 | United Kingdom | | Equine | 2007 | | H3N8 | | NP | | KF559335 | |  |
|  | A/equine/Richmond/1/2007/H3N8 | United Kingdom | | Equine | 2007 | | H3N8 | | NA | | KF559336 | |  |
|  | A/equine/Richmond/1/2007/H3N8 | United Kingdom | | Equine | 2007 | | H3N8 | | MP | | KF559337 | |  |
|  | A/equine/Richmond/1/2007/H3N8 | United Kingdom | | Equine | 2007 | | H3N8 | | NS | | FJ195429 | |  |
|  | A/equine/Tottori/1/07/H3N8 | Japan | | Equine | 2007 | | H3N8 | | PB2 | | AB591847 | |  |
|  | A/equine/Tottori/1/07/H3N8 | Japan | | Equine | 2007 | | H3N8 | | PB1 | | AB591846 | |  |
|  | A/equine/Tottori/1/07/H3N8 | Japan | | Equine | 2007 | | H3N8 | | PA | | AB591845 | |  |
|  | A/equine/Tottori/1/07/H3N8 | Japan | | Equine | 2007 | | H3N8 | | HA | | AB591842 | |  |
|  | A/equine/Tottori/1/07/H3N8 | Japan | | Equine | 2007 | | H3N8 | | NP | | AB591844 | |  |
|  | A/equine/Tottori/1/07/H3N8 | Japan | | Equine | 2007 | | H3N8 | | NA | | AB591843 | |  |
|  | A/equine/Tottori/1/07/H3N8 | Japan | | Equine | 2007 | | H3N8 | | MP | | AB591848 | |  |
|  | A/equine/Tottori/1/07/H3N8 | Japan | | Equine | 2007 | | H3N8 | | NS | | AB591849 | |  |
|  | A/equine/Xinjiang/1/2007/H3N8 | China | | Equine | 2007 | | H3N8 | | PB2 | | EU794540 | |  |
|  | A/equine/Xinjiang/1/2007/H3N8 | China | | Equine | 2007 | | H3N8 | | PB1 | | EU794541 | |  |
|  | A/equine/Xinjiang/1/2007/H3N8 | China | | Equine | 2007 | | H3N8 | | PA | | EU794542 | |  |
|  | A/equine/Xinjiang/1/2007/H3N8 | China | | Equine | 2007 | | H3N8 | | HA | | EU794543 | |  |
|  | A/equine/Xinjiang/1/2007/H3N8 | China | | Equine | 2007 | | H3N8 | | NP | | EU794544 | |  |
|  | A/equine/Xinjiang/1/2007/H3N8 | China | | Equine | 2007 | | H3N8 | | NA | | EU794545 | |  |
|  | A/equine/Xinjiang/1/2007/H3N8 | China | | Equine | 2007 | | H3N8 | | MP | | EU794546 | |  |
|  | A/equine/Xinjiang/1/2007/H3N8 | China | | Equine | 2007 | | H3N8 | | NS | | EU794547 | |  |
|  | A/equine/Xinjiang/2/2007/H3N8 | China | | Equine | 2007 | | H3N8 | | PB2 | | EU794548 | |  |
|  | A/equine/Xinjiang/2/2007/H3N8 | China | | Equine | 2007 | | H3N8 | | PB1 | | EU794549 | |  |
|  | A/equine/Xinjiang/2/2007/H3N8 | China | | Equine | 2007 | | H3N8 | | PA | | EU794550 | |  |
|  | A/equine/Xinjiang/2/2007/H3N8 | China | | Equine | 2007 | | H3N8 | | HA | | EU794551 | |  |
|  | A/equine/Xinjiang/2/2007/H3N8 | China | | Equine | 2007 | | H3N8 | | NP | | EU794552 | |  |
|  | A/equine/Xinjiang/2/2007/H3N8 | China | | Equine | 2007 | | H3N8 | | NA | | EU794553 | |  |
|  | A/equine/Xinjiang/2/2007/H3N8 | China | | Equine | 2007 | | H3N8 | | MP | | EU794554 | |  |
|  | A/equine/Xinjiang/2/2007/H3N8 | China | | Equine | 2007 | | H3N8 | | NS | | EU794555 | |  |
|  | A/equine/Xinjiang/3/2007/H3N8 | China | | Equine | 2007 | | H3N8 | | PB2 | | EU794556 | |  |
|  | A/equine/Xinjiang/3/2007/H3N8 | China | | Equine | 2007 | | H3N8 | | PB1 | | EU794557 | |  |
|  | A/equine/Xinjiang/3/2007/H3N8 | China | | Equine | 2007 | | H3N8 | | PA | | EU794558 | |  |
|  | A/equine/Xinjiang/3/2007/H3N8 | China | | Equine | 2007 | | H3N8 | | HA | | EU794559 | |  |
|  | A/equine/Xinjiang/3/2007/H3N8 | China | | Equine | 2007 | | H3N8 | | NP | | EU794560 | |  |
|  | A/equine/Xinjiang/3/2007/H3N8 | China | | Equine | 2007 | | H3N8 | | NA | | EU794561 | |  |
|  | A/equine/Xinjiang/3/2007/H3N8 | China | | Equine | 2007 | | H3N8 | | MP | | EU794562 | |  |
|  | A/equine/Xinjiang/3/2007/H3N8 | China | | Equine | 2007 | | H3N8 | | NS | | EU794563 | |  |
|  | A/equine/Xinjiang/4/2007/H3N8 | China | | Equine | 2007 | | H3N8 | | PB2 | | EU794564 | |  |
|  | A/equine/Xinjiang/4/2007/H3N8 | China | | Equine | 2007 | | H3N8 | | PB1 | | EU794565 | |  |
|  | A/equine/Xinjiang/4/2007/H3N8 | China | | Equine | 2007 | | H3N8 | | PA | | EU794566 | |  |
|  | A/equine/Xinjiang/4/2007/H3N8 | China | | Equine | 2007 | | H3N8 | | HA | | EU794567 | |  |
|  | A/equine/Xinjiang/4/2007/H3N8 | China | | Equine | 2007 | | H3N8 | | NP | | EU794568 | |  |
|  | A/equine/Xinjiang/4/2007/H3N8 | China | | Equine | 2007 | | H3N8 | | NA | | EU794569 | |  |
|  | A/equine/Xinjiang/4/2007/H3N8 | China | | Equine | 2007 | | H3N8 | | MP | | EU794570 | |  |
|  | A/equine/Xinjiang/4/2007/H3N8 | China | | Equine | 2007 | | H3N8 | | NS | | EU794571 | |  |
|  | A/equine/Newmarket/5/2003/H3N8 | United Kingdom | | Equine | 2003 | | H3N8 | | PB2 | | FJ375221 | |  |
|  | A/equine/Newmarket/5/2003/H3N8 | United Kingdom | | Equine | 2003 | | H3N8 | | PB1 | | FJ375233 | |  |
|  | A/equine/Newmarket/5/2003/H3N8 | United Kingdom | | Equine | 2003 | | H3N8 | | PA | | FJ375228 | |  |
|  | A/equine/Newmarket/5/2003/H3N8 | United Kingdom | | Equine | 2003 | | H3N8 | | HA | | FJ375213 | |  |
|  | A/equine/Newmarket/5/2003/H3N8 | United Kingdom | | Equine | 2003 | | H3N8 | | NP | | FJ375216 | |  |
|  | A/equine/Newmarket/5/2003/H3N8 | United Kingdom | | Equine | 2003 | | H3N8 | | NA | | FJ375224 | |  |
|  | A/equine/Newmarket/5/2003/H3N8 | United Kingdom | | Equine | 2003 | | H3N8 | | MP | | FJ375236 | |  |
|  | A/equine/Newmarket/5/2003/H3N8 | United Kingdom | | Equine | 2003 | | H3N8 | | NS | | FJ375209 | |  |
|  | A/equine/Wisconsin/1/03/H3N8 | USA | | Equine | 2003 | | H3N8 | | PB2 | | DQ222920 | |  |
|  | A/equine/Wisconsin/1/03/H3N8 | USA | | Equine | 2003 | | H3N8 | | PB1 | | DQ222919 | |  |
|  | A/equine/Wisconsin/1/03/H3N8 | USA | | Equine | 2003 | | H3N8 | | PA | | DQ222918 | |  |
|  | A/equine/Wisconsin/1/03/H3N8 | USA | | Equine | 2003 | | H3N8 | | HA | | DQ222913 | |  |
|  | A/equine/Wisconsin/1/03/H3N8 | USA | | Equine | 2003 | | H3N8 | | NP | | DQ222915 | |  |
|  | A/equine/Wisconsin/1/03/H3N8 | USA | | Equine | 2003 | | H3N8 | | NA | | DQ222914 | |  |
|  | A/equine/Wisconsin/1/03/H3N8 | USA | | Equine | 2003 | | H3N8 | | MP | | DQ222916 | |  |
|  | A/equine/Wisconsin/1/03/H3N8 | USA | | Equine | 2003 | | H3N8 | | NS | | DQ222917 | |  |
|  | A/equine/California/8560/2002/H3N8 | USA | | Equine | 2002 | | H3N8 | | PB2 | | CY030766 | |  |
|  | A/equine/California/8560/2002/H3N8 | USA | | Equine | 2002 | | H3N8 | | PB1 | | CY030765 | |  |
|  | A/equine/California/8560/2002/H3N8 | USA | | Equine | 2002 | | H3N8 | | PA | | CY030764 | |  |
|  | A/equine/California/8560/2002/H3N8 | USA | | Equine | 2002 | | H3N8 | | HA | | CY030759 | |  |
|  | A/equine/California/8560/2002/H3N8 | USA | | Equine | 2002 | | H3N8 | | NP | | CY030762 | |  |
|  | A/equine/California/8560/2002/H3N8 | USA | | Equine | 2002 | | H3N8 | | NA | | CY030761 | |  |
|  | A/equine/California/8560/2002/H3N8 | USA | | Equine | 2002 | | H3N8 | | MP | | CY030760 | |  |
|  | A/equine/California/8560/2002/H3N8 | USA | | Equine | 2002 | | H3N8 | | NS | | CY030763 | |  |
|  | A/equine/Kentucky/5/2002/H3N8 | USA | | Equine | 2002 | | H3N8 | | PB2 | | AY855338 | |  |
|  | A/equine/Kentucky/5/2002/H3N8 | USA | | Equine | 2002 | | H3N8 | | PB1 | | AY855339 | |  |
|  | A/equine/Kentucky/5/2002/H3N8 | USA | | Equine | 2002 | | H3N8 | | PA | | AY855340 | |  |
|  | A/equine/Kentucky/5/2002/H3N8 | USA | | Equine | 2002 | | H3N8 | | HA | | AY855341 | |  |
|  | A/equine/Kentucky/5/2002/H3N8 | USA | | Equine | 2002 | | H3N8 | | NP | | AY855342 | |  |
|  | A/equine/Kentucky/5/2002/H3N8 | USA | | Equine | 2002 | | H3N8 | | NA | | AY855343 | |  |
|  | A/equine/Kentucky/5/2002/H3N8 | USA | | Equine | 2002 | | H3N8 | | MP | | AY855344 | |  |
|  | A/equine/Kentucky/5/2002/H3N8 | USA | | Equine | 2002 | | H3N8 | | NS | | AY855345 | |  |
|  | A/equine/California/4537/1997/H3N8 | USA | | Equine | 1997 | | H3N8 | | PB2 | | CY030758 | |  |
|  | A/equine/California/4537/1997/H3N8 | USA | | Equine | 1997 | | H3N8 | | PB1 | | CY030757 | |  |
|  | A/equine/California/4537/1997/H3N8 | USA | | Equine | 1997 | | H3N8 | | PA | | CY030756 | |  |
|  | A/equine/California/4537/1997/H3N8 | USA | | Equine | 1997 | | H3N8 | | HA | | CY030751 | |  |
|  | A/equine/California/4537/1997/H3N8 | USA | | Equine | 1997 | | H3N8 | | NP | | CY030754 | |  |
|  | A/equine/California/4537/1997/H3N8 | USA | | Equine | 1997 | | H3N8 | | NA | | CY030753 | |  |
|  | A/equine/California/4537/1997/H3N8 | USA | | Equine | 1997 | | H3N8 | | MP | | CY030752 | |  |
|  | A/equine/California/4537/1997/H3N8 | USA | | Equine | 1997 | | H3N8 | | NS | | CY030755 | |  |
|  | A/equine/Kentucky/8/1994/H3N8 | USA | | Equine | 1994 | | H3N8 | | PB2 | | CY030188 | |  |
|  | A/equine/Kentucky/8/1994/H3N8 | USA | | Equine | 1994 | | H3N8 | | PB1 | | CY030187 | |  |
|  | A/equine/Kentucky/8/1994/H3N8 | USA | | Equine | 1994 | | H3N8 | | PA | | CY030186 | |  |
|  | A/equine/Kentucky/8/1994/H3N8 | USA | | Equine | 1994 | | H3N8 | | HA | | CY030181 | |  |
|  | A/equine/Kentucky/8/1994/H3N8 | USA | | Equine | 1994 | | H3N8 | | NP | | CY030184 | |  |
|  | A/equine/Kentucky/8/1994/H3N8 | USA | | Equine | 1994 | | H3N8 | | NA | | CY030183 | |  |
|  | A/equine/Kentucky/8/1994/H3N8 | USA | | Equine | 1994 | | H3N8 | | MP | | CY030182 | |  |
|  | A/equine/Kentucky/8/1994/H3N8 | USA | | Equine | 1994 | | H3N8 | | NS | | CY030185 | |  |
|  | A/equine/Qinghai/1/1994/H3N8 | China | | Equine | 1994 | | H3N8 | | PB2 | | EU794532 | |  |
|  | A/equine/Qinghai/1/1994/H3N8 | China | | Equine | 1994 | | H3N8 | | PB1 | | EU794533 | |  |
|  | A/equine/Qinghai/1/1994/H3N8 | China | | Equine | 1994 | | H3N8 | | PA | | EU794534 | |  |
|  | A/equine/Qinghai/1/1994/H3N8 | China | | Equine | 1994 | | H3N8 | | HA | | EU794535 | |  |
|  | A/equine/Qinghai/1/1994/H3N8 | China | | Equine | 1994 | | H3N8 | | NP | | EU794536 | |  |
|  | A/equine/Qinghai/1/1994/H3N8 | China | | Equine | 1994 | | H3N8 | | NA | | EU794537 | |  |
|  | A/equine/Qinghai/1/1994/H3N8 | China | | Equine | 1994 | | H3N8 | | MP | | EU794538 | |  |
|  | A/equine/Qinghai/1/1994/H3N8 | China | | Equine | 1994 | | H3N8 | | NS | | EU794539 | |  |
|  | A/equine/Switzerland/173/1993/H3N8 | Switzerland | | Equine | 1993 | | H3N8 | | PB2 | | CY032364 | |  |
|  | A/equine/Switzerland/173/1993/H3N8 | Switzerland | | Equine | 1993 | | H3N8 | | PB1 | | CY032363 | |  |
|  | A/equine/Switzerland/173/1993/H3N8 | Switzerland | | Equine | 1993 | | H3N8 | | PA | | CY032362 | |  |
|  | A/equine/Switzerland/173/1993/H3N8 | Switzerland | | Equine | 1993 | | H3N8 | | HA | | CY032357 | |  |
|  | A/equine/Switzerland/173/1993/H3N8 | Switzerland | | Equine | 1993 | | H3N8 | | NP | | CY032360 | |  |
|  | A/equine/Switzerland/173/1993/H3N8 | Switzerland | | Equine | 1993 | | H3N8 | | NA | | CY032359 | |  |
|  | A/equine/Switzerland/173/1993/H3N8 | Switzerland | | Equine | 1993 | | H3N8 | | MP | | CY032358 | |  |
|  | A/equine/Switzerland/173/1993/H3N8 | Switzerland | | Equine | 1993 | | H3N8 | | NS | | CY032361 | |  |
|  | A/equine/Austria/421/1992/H3N8 | Austria | | Equine | 1992 | | H3N8 | | PB2 | | CY032356 | |  |
|  | A/equine/Austria/421/1992/H3N8 | Austria | | Equine | 1992 | | H3N8 | | PB1 | | CY032355 | |  |
|  | A/equine/Austria/421/1992/H3N8 | Austria | | Equine | 1992 | | H3N8 | | PA | | CY032354 | |  |
|  | A/equine/Austria/421/1992/H3N8 | Austria | | Equine | 1992 | | H3N8 | | HA | | CY032349 | |  |
|  | A/equine/Austria/421/1992/H3N8 | Austria | | Equine | 1992 | | H3N8 | | NP | | CY032352 | |  |
|  | A/equine/Austria/421/1992/H3N8 | Austria | | Equine | 1992 | | H3N8 | | NA | | CY032351 | |  |
|  | A/equine/Austria/421/1992/H3N8 | Austria | | Equine | 1992 | | H3N8 | | MP | | CY032350 | |  |
|  | A/equine/Austria/421/1992/H3N8 | Austria | | Equine | 1992 | | H3N8 | | NS | | CY032353 | |  |
|  | A/equine/Italy/1199/1992/H3N8 | Italy | | Equine | 1992 | | H3N8 | | PB2 | | CY032348 | |  |
|  | A/equine/Italy/1199/1992/H3N8 | Italy | | Equine | 1992 | | H3N8 | | PB1 | | CY032347 | |  |
|  | A/equine/Italy/1199/1992/H3N8 | Italy | | Equine | 1992 | | H3N8 | | PA | | CY032346 | |  |
|  | A/equine/Italy/1199/1992/H3N8 | Italy | | Equine | 1992 | | H3N8 | | HA | | CY032341 | |  |
|  | A/equine/Italy/1199/1992/H3N8 | Italy | | Equine | 1992 | | H3N8 | | NP | | CY032344 | |  |
|  | A/equine/Italy/1199/1992/H3N8 | Italy | | Equine | 1992 | | H3N8 | | NA | | CY032343 | |  |
|  | A/equine/Italy/1199/1992/H3N8 | Italy | | Equine | 1992 | | H3N8 | | MP | | CY032342 | |  |
|  | A/equine/Italy/1199/1992/H3N8 | Italy | | Equine | 1992 | | H3N8 | | NS | | CY032345 | |  |
|  | A/equine/Kentucky/1/1992/H3N8 | USA | | Equine | 1992 | | H3N8 | | PB2 | | CY030156 | |  |
|  | A/equine/Kentucky/1/1992/H3N8 | USA | | Equine | 1992 | | H3N8 | | PB1 | | CY030155 | |  |
|  | A/equine/Kentucky/1/1992/H3N8 | USA | | Equine | 1992 | | H3N8 | | PA | | CY030154 | |  |
|  | A/equine/Kentucky/1/1992/H3N8 | USA | | Equine | 1992 | | H3N8 | | HA | | CY030149 | |  |
|  | A/equine/Kentucky/1/1992/H3N8 | USA | | Equine | 1992 | | H3N8 | | NP | | CY030152 | |  |
|  | A/equine/Kentucky/1/1992/H3N8 | USA | | Equine | 1992 | | H3N8 | | NA | | CY030151 | |  |
|  | A/equine/Kentucky/1/1992/H3N8 | USA | | Equine | 1992 | | H3N8 | | MP | | CY030150 | |  |
|  | A/equine/Kentucky/1/1992/H3N8 | USA | | Equine | 1992 | | H3N8 | | NS | | CY030153 | |  |
|  | A/equine/Alaska/29759/1991/H3N8 | USA | | Equine | 1991 | | H3N8 | | PB2 | | CY030164 | |  |
|  | A/equine/Alaska/29759/1991/H3N8 | USA | | Equine | 1991 | | H3N8 | | PB1 | | CY030163 | |  |
|  | A/equine/Alaska/29759/1991/H3N8 | USA | | Equine | 1991 | | H3N8 | | PA | | CY030162 | |  |
|  | A/equine/Alaska/29759/1991/H3N8 | USA | | Equine | 1991 | | H3N8 | | HA | | CY030157 | |  |
|  | A/equine/Alaska/29759/1991/H3N8 | USA | | Equine | 1991 | | H3N8 | | NP | | CY030160 | |  |
|  | A/equine/Alaska/29759/1991/H3N8 | USA | | Equine | 1991 | | H3N8 | | NA | | CY030159 | |  |
|  | A/equine/Alaska/29759/1991/H3N8 | USA | | Equine | 1991 | | H3N8 | | MP | | CY030158 | |  |
|  | A/equine/Alaska/29759/1991/H3N8 | USA | | Equine | 1991 | | H3N8 | | NS | | CY030161 | |  |
|  | A/equine/Italy/1062/1991/H3N8 | Italy | | Equine | 1991 | | H3N8 | | PB2 | | CY032380 | |  |
|  | A/equine/Italy/1062/1991/H3N8 | Italy | | Equine | 1991 | | H3N8 | | PB1 | | CY032379 | |  |
|  | A/equine/Italy/1062/1991/H3N8 | Italy | | Equine | 1991 | | H3N8 | | PA | | CY032378 | |  |
|  | A/equine/Italy/1062/1991/H3N8 | Italy | | Equine | 1991 | | H3N8 | | HA | | CY032373 | |  |
|  | A/equine/Italy/1062/1991/H3N8 | Italy | | Equine | 1991 | | H3N8 | | NP | | CY032376 | |  |
|  | A/equine/Italy/1062/1991/H3N8 | Italy | | Equine | 1991 | | H3N8 | | NA | | CY032375 | |  |
|  | A/equine/Italy/1062/1991/H3N8 | Italy | | Equine | 1991 | | H3N8 | | MP | | CY032374 | |  |
|  | A/equine/Italy/1062/1991/H3N8 | Italy | | Equine | 1991 | | H3N8 | | NS | | CY032377 | |  |
|  | A/equine/Italy/824/1991/H3N8 | Italy | | Equine | 1991 | | H3N8 | | PB2 | | CY032340 | |  |
|  | A/equine/Italy/824/1991/H3N8 | Italy | | Equine | 1991 | | H3N8 | | PB1 | | CY032339 | |  |
|  | A/equine/Italy/824/1991/H3N8 | Italy | | Equine | 1991 | | H3N8 | | PA | | CY032338 | |  |
|  | A/equine/Italy/824/1991/H3N8 | Italy | | Equine | 1991 | | H3N8 | | HA | | CY032333 | |  |
|  | A/equine/Italy/824/1991/H3N8 | Italy | | Equine | 1991 | | H3N8 | | NP | | CY032336 | |  |
|  | A/equine/Italy/824/1991/H3N8 | Italy | | Equine | 1991 | | H3N8 | | NA | | CY032335 | |  |
|  | A/equine/Italy/824/1991/H3N8 | Italy | | Equine | 1991 | | H3N8 | | MP | | CY032334 | |  |
|  | A/equine/Italy/824/1991/H3N8 | Italy | | Equine | 1991 | | H3N8 | | NS | | CY032337 | |  |
|  | A/equine/Kentucky/1/1991/H3N8 | USA | | Equine | 1991 | | H3N8 | | PB2 | | CY030180 | |  |
|  | A/equine/Kentucky/1/1991/H3N8 | USA | | Equine | 1991 | | H3N8 | | PB1 | | CY030179 | |  |
|  | A/equine/Kentucky/1/1991/H3N8 | USA | | Equine | 1991 | | H3N8 | | PA | | CY030178 | |  |
|  | A/equine/Kentucky/1/1991/H3N8 | USA | | Equine | 1991 | | H3N8 | | HA | | CY030173 | |  |
|  | A/equine/Kentucky/1/1991/H3N8 | USA | | Equine | 1991 | | H3N8 | | NP | | CY030176 | |  |
|  | A/equine/Kentucky/1/1991/H3N8 | USA | | Equine | 1991 | | H3N8 | | NA | | CY030175 | |  |
|  | A/equine/Kentucky/1/1991/H3N8 | USA | | Equine | 1991 | | H3N8 | | MP | | CY030174 | |  |
|  | A/equine/Kentucky/1/1991/H3N8 | USA | | Equine | 1991 | | H3N8 | | NS | | CY030177 | |  |
|  | A/equine/Rome/5/1991/H3N8 | Italy | | Equine | 1991 | | H3N8 | | PB2 | | CY032372 | |  |
|  | A/equine/Rome/5/1991/H3N8 | Italy | | Equine | 1991 | | H3N8 | | PB1 | | CY032371 | |  |
|  | A/equine/Rome/5/1991/H3N8 | Italy | | Equine | 1991 | | H3N8 | | PA | | CY032370 | |  |
|  | A/equine/Rome/5/1991/H3N8 | Italy | | Equine | 1991 | | H3N8 | | HA | | CY032365 | |  |
|  | A/equine/Rome/5/1991/H3N8 | Italy | | Equine | 1991 | | H3N8 | | NP | | CY032368 | |  |
|  | A/equine/Rome/5/1991/H3N8 | Italy | | Equine | 1991 | | H3N8 | | NA | | CY032367 | |  |
|  | A/equine/Rome/5/1991/H3N8 | Italy | | Equine | 1991 | | H3N8 | | MP | | CY032366 | |  |
|  | A/equine/Rome/5/1991/H3N8 | Italy | | Equine | 1991 | | H3N8 | | NS | | CY032369 | |  |
|  | A/equine/Texas/39655/1991/H3N8 | USA | | Equine | 1991 | | H3N8 | | PB2 | | CY030148 | |  |
|  | A/equine/Texas/39655/1991/H3N8 | USA | | Equine | 1991 | | H3N8 | | PB1 | | CY030147 | |  |
|  | A/equine/Texas/39655/1991/H3N8 | USA | | Equine | 1991 | | H3N8 | | PA | | CY030146 | |  |
|  | A/equine/Texas/39655/1991/H3N8 | USA | | Equine | 1991 | | H3N8 | | HA | | CY030141 | |  |
|  | A/equine/Texas/39655/1991/H3N8 | USA | | Equine | 1991 | | H3N8 | | NP | | CY030144 | |  |
|  | A/equine/Texas/39655/1991/H3N8 | USA | | Equine | 1991 | | H3N8 | | NA | | CY030143 | |  |
|  | A/equine/Texas/39655/1991/H3N8 | USA | | Equine | 1991 | | H3N8 | | MP | | CY030142 | |  |
|  | A/equine/Texas/39655/1991/H3N8 | USA | | Equine | 1991 | | H3N8 | | NS | | CY030145 | |  |
|  | A/equine/Kentucky/1277/1990/H3N8 | USA | | Equine | 1990 | | H3N8 | | PB2 | | CY030140 | |  |
|  | A/equine/Kentucky/1277/1990/H3N8 | USA | | Equine | 1990 | | H3N8 | | PB1 | | CY030139 | |  |
|  | A/equine/Kentucky/1277/1990/H3N8 | USA | | Equine | 1990 | | H3N8 | | PA | | CY030138 | |  |
|  | A/equine/Kentucky/1277/1990/H3N8 | USA | | Equine | 1990 | | H3N8 | | HA | | CY030133 | |  |
|  | A/equine/Kentucky/1277/1990/H3N8 | USA | | Equine | 1990 | | H3N8 | | NP | | CY030136 | |  |
|  | A/equine/Kentucky/1277/1990/H3N8 | USA | | Equine | 1990 | | H3N8 | | NA | | CY030135 | |  |
|  | A/equine/Kentucky/1277/1990/H3N8 | USA | | Equine | 1990 | | H3N8 | | MP | | CY030134 | |  |
|  | A/equine/Kentucky/1277/1990/H3N8 | USA | | Equine | 1990 | | H3N8 | | NS | | CY030137 | |  |
|  | A/equine/Berlin/1/1989/H3N8 | Germany | | Equine | 1989 | | H3N8 | | PB2 | | CY032420 | |  |
|  | A/equine/Berlin/1/1989/H3N8 | Germany | | Equine | 1989 | | H3N8 | | PB1 | | CY032419 | |  |
|  | A/equine/Berlin/1/1989/H3N8 | Germany | | Equine | 1989 | | H3N8 | | PA | | CY032418 | |  |
|  | A/equine/Berlin/1/1989/H3N8 | Germany | | Equine | 1989 | | H3N8 | | HA | | CY032413 | |  |
|  | A/equine/Berlin/1/1989/H3N8 | Germany | | Equine | 1989 | | H3N8 | | NP | | CY032416 | |  |
|  | A/equine/Berlin/1/1989/H3N8 | Germany | | Equine | 1989 | | H3N8 | | NA | | CY032415 | |  |
|  | A/equine/Berlin/1/1989/H3N8 | Germany | | Equine | 1989 | | H3N8 | | MP | | CY032414 | |  |
|  | A/equine/Berlin/1/1989/H3N8 | Germany | | Equine | 1989 | | H3N8 | | NS | | CY032417 | |  |
|  | A/equine/Jilin/1/1989/H3N8 | China | | Equine | 1989 | | H3N8 | | PB2 | | KF285454 | |  |
|  | A/equine/Jilin/1/1989/H3N8 | China | | Equine | 1989 | | H3N8 | | PB1 | | KF285455 | |  |
|  | A/equine/Jilin/1/1989/H3N8 | China | | Equine | 1989 | | H3N8 | | PA | | KF285456 | |  |
|  | A/equine/Jilin/1/1989/H3N8 | China | | Equine | 1989 | | H3N8 | | HA | | M65018 | |  |
|  | A/equine/Jilin/1/1989/H3N8 | China | | Equine | 1989 | | H3N8 | | NP | | M63786 | |  |
|  | A/equine/Jilin/1/1989/H3N8 | China | | Equine | 1989 | | H3N8 | | NA | | L06579 | |  |
|  | A/equine/Jilin/1/1989/H3N8 | China | | Equine | 1989 | | H3N8 | | MP | | M65019 | |  |
|  | A/equine/Jilin/1/1989/H3N8 | China | | Equine | 1989 | | H3N8 | | NS | | M65020 | |  |
|  | A/equine/Rook/93753/1989/H3N8 | United Kingdom | | Equine | 1989 | | H3N8 | | PB2 | | CY032332 | |  |
|  | A/equine/Rook/93753/1989/H3N8 | United Kingdom | | Equine | 1989 | | H3N8 | | PB1 | | CY032331 | |  |
|  | A/equine/Rook/93753/1989/H3N8 | United Kingdom | | Equine | 1989 | | H3N8 | | PA | | CY032330 | |  |
|  | A/equine/Rook/93753/1989/H3N8 | United Kingdom | | Equine | 1989 | | H3N8 | | HA | | CY032325 | |  |
|  | A/equine/Rook/93753/1989/H3N8 | United Kingdom | | Equine | 1989 | | H3N8 | | NP | | CY032328 | |  |
|  | A/equine/Rook/93753/1989/H3N8 | United Kingdom | | Equine | 1989 | | H3N8 | | NA | | CY032327 | |  |
|  | A/equine/Rook/93753/1989/H3N8 | United Kingdom | | Equine | 1989 | | H3N8 | | MP | | CY032326 | |  |
|  | A/equine/Rook/93753/1989/H3N8 | United Kingdom | | Equine | 1989 | | H3N8 | | NS | | CY032329 | |  |
|  | A/equine/Sussex/1/1989/H3N8 | United Kingdom | | Equine | 1989 | | H3N8 | | PB2 | | CY032324 | |  |
|  | A/equine/Sussex/1/1989/H3N8 | United Kingdom | | Equine | 1989 | | H3N8 | | PB1 | | CY032323 | |  |
|  | A/equine/Sussex/1/1989/H3N8 | United Kingdom | | Equine | 1989 | | H3N8 | | PA | | CY032322 | |  |
|  | A/equine/Sussex/1/1989/H3N8 | United Kingdom | | Equine | 1989 | | H3N8 | | HA | | CY032317 | |  |
|  | A/equine/Sussex/1/1989/H3N8 | United Kingdom | | Equine | 1989 | | H3N8 | | NP | | CY032320 | |  |
|  | A/equine/Sussex/1/1989/H3N8 | United Kingdom | | Equine | 1989 | | H3N8 | | NA | | CY032319 | |  |
|  | A/equine/Sussex/1/1989/H3N8 | United Kingdom | | Equine | 1989 | | H3N8 | | MP | | CY032318 | |  |
|  | A/equine/Sussex/1/1989/H3N8 | United Kingdom | | Equine | 1989 | | H3N8 | | NS | | CY032321 | |  |
|  | A/equine/Kentucky/692/1988/H3N8 | USA | | Equine | 1988 | | H3N8 | | PB2 | | CY030116 | |  |
|  | A/equine/Kentucky/692/1988/H3N8 | USA | | Equine | 1988 | | H3N8 | | PB1 | | CY030115 | |  |
|  | A/equine/Kentucky/692/1988/H3N8 | USA | | Equine | 1988 | | H3N8 | | PA | | CY030114 | |  |
|  | A/equine/Kentucky/692/1988/H3N8 | USA | | Equine | 1988 | | H3N8 | | HA | | CY030109 | |  |
|  | A/equine/Kentucky/692/1988/H3N8 | USA | | Equine | 1988 | | H3N8 | | NP | | CY030112 | |  |
|  | A/equine/Kentucky/692/1988/H3N8 | USA | | Equine | 1988 | | H3N8 | | NA | | CY030111 | |  |
|  | A/equine/Kentucky/692/1988/H3N8 | USA | | Equine | 1988 | | H3N8 | | MP | | CY030110 | |  |
|  | A/equine/Kentucky/692/1988/H3N8 | USA | | Equine | 1988 | | H3N8 | | NS | | CY030113 | |  |
|  | A/equine/Kentucky/694/1988/H3N8 | USA | | Equine | 1988 | | H3N8 | | PB2 | | CY030124 | |  |
|  | A/equine/Kentucky/694/1988/H3N8 | USA | | Equine | 1988 | | H3N8 | | PB1 | | CY030123 | |  |
|  | A/equine/Kentucky/694/1988/H3N8 | USA | | Equine | 1988 | | H3N8 | | PA | | CY030122 | |  |
|  | A/equine/Kentucky/694/1988/H3N8 | USA | | Equine | 1988 | | H3N8 | | HA | | CY030117 | |  |
|  | A/equine/Kentucky/694/1988/H3N8 | USA | | Equine | 1988 | | H3N8 | | NP | | CY030120 | |  |
|  | A/equine/Kentucky/694/1988/H3N8 | USA | | Equine | 1988 | | H3N8 | | NA | | CY030119 | |  |
|  | A/equine/Kentucky/694/1988/H3N8 | USA | | Equine | 1988 | | H3N8 | | MP | | CY030118 | |  |
|  | A/equine/Kentucky/694/1988/H3N8 | USA | | Equine | 1988 | | H3N8 | | NS | | CY030121 | |  |
|  | A/equine/Kentucky/698/1988/H3N8 | USA | | Equine | 1988 | | H3N8 | | PB2 | | CY030132 | |  |
|  | A/equine/Kentucky/698/1988/H3N8 | USA | | Equine | 1988 | | H3N8 | | PB1 | | CY030131 | |  |
|  | A/equine/Kentucky/698/1988/H3N8 | USA | | Equine | 1988 | | H3N8 | | PA | | CY030130 | |  |
|  | A/equine/Kentucky/698/1988/H3N8 | USA | | Equine | 1988 | | H3N8 | | HA | | CY030125 | |  |
|  | A/equine/Kentucky/698/1988/H3N8 | USA | | Equine | 1988 | | H3N8 | | NP | | CY030128 | |  |
|  | A/equine/Kentucky/698/1988/H3N8 | USA | | Equine | 1988 | | H3N8 | | NA | | CY030127 | |  |
|  | A/equine/Kentucky/698/1988/H3N8 | USA | | Equine | 1988 | | H3N8 | | MP | | CY030126 | |  |
|  | A/equine/Kentucky/698/1988/H3N8 | USA | | Equine | 1988 | | H3N8 | | NS | | CY030129 | |  |
|  | A/equine/Kentucky/1/1987/H3N8 | USA | | Equine | 1987 | | H3N8 | | PB2 | | CY030108 | |  |
|  | A/equine/Kentucky/1/1987/H3N8 | USA | | Equine | 1987 | | H3N8 | | PB1 | | CY030107 | |  |
|  | A/equine/Kentucky/1/1987/H3N8 | USA | | Equine | 1987 | | H3N8 | | PA | | CY030106 | |  |
|  | A/equine/Kentucky/1/1987/H3N8 | USA | | Equine | 1987 | | H3N8 | | HA | | CY030101 | |  |
|  | A/equine/Kentucky/1/1987/H3N8 | USA | | Equine | 1987 | | H3N8 | | NP | | CY030104 | |  |
|  | A/equine/Kentucky/1/1987/H3N8 | USA | | Equine | 1987 | | H3N8 | | NA | | CY030103 | |  |
|  | A/equine/Kentucky/1/1987/H3N8 | USA | | Equine | 1987 | | H3N8 | | MP | | CY030102 | |  |
|  | A/equine/Kentucky/1/1987/H3N8 | USA | | Equine | 1987 | | H3N8 | | NS | | CY030105 | |  |
|  | A/equine/Kentucky/2/1987/H3N8 | USA | | Equine | 1987 | | H3N8 | | PB2 | | CY031545 | |  |
|  | A/equine/Kentucky/2/1987/H3N8 | USA | | Equine | 1987 | | H3N8 | | PB1 | | CY031544 | |  |
|  | A/equine/Kentucky/2/1987/H3N8 | USA | | Equine | 1987 | | H3N8 | | PA | | CY031543 | |  |
|  | A/equine/Kentucky/2/1987/H3N8 | USA | | Equine | 1987 | | H3N8 | | HA | | CY031538 | |  |
|  | A/equine/Kentucky/2/1987/H3N8 | USA | | Equine | 1987 | | H3N8 | | NP | | CY031541 | |  |
|  | A/equine/Kentucky/2/1987/H3N8 | USA | | Equine | 1987 | | H3N8 | | NA | | CY031540 | |  |
|  | A/equine/Kentucky/2/1987/H3N8 | USA | | Equine | 1987 | | H3N8 | | MP | | CY031539 | |  |
|  | A/equine/Kentucky/2/1987/H3N8 | USA | | Equine | 1987 | | H3N8 | | NS | | CY031542 | |  |
|  | A/equine/Johannesburg/1/1986/H3N8 | South Africa | | Equine | 1986 | | H3N8 | | PB2 | | CY032960 | |  |
|  | A/equine/Johannesburg/1/1986/H3N8 | South Africa | | Equine | 1986 | | H3N8 | | PB1 | | CY032959 | |  |
|  | A/equine/Johannesburg/1/1986/H3N8 | South Africa | | Equine | 1986 | | H3N8 | | PA | | CY032958 | |  |
|  | A/equine/Johannesburg/1/1986/H3N8 | South Africa | | Equine | 1986 | | H3N8 | | HA | | CY032953 | |  |
|  | A/equine/Johannesburg/1/1986/H3N8 | South Africa | | Equine | 1986 | | H3N8 | | NP | | CY032956 | |  |
|  | A/equine/Johannesburg/1/1986/H3N8 | South Africa | | Equine | 1986 | | H3N8 | | NA | | CY032955 | |  |
|  | A/equine/Johannesburg/1/1986/H3N8 | South Africa | | Equine | 1986 | | H3N8 | | MP | | CY032954 | |  |
|  | A/equine/Johannesburg/1/1986/H3N8 | South Africa | | Equine | 1986 | | H3N8 | | NS | | CY032957 | |  |
|  | A/equine/Kentucky/1/1986/H3N8 | USA | | Equine | 1986 | | H3N8 | | PB2 | | CY030100 | |  |
|  | A/equine/Kentucky/1/1986/H3N8 | USA | | Equine | 1986 | | H3N8 | | PB1 | | CY030099 | |  |
|  | A/equine/Kentucky/1/1986/H3N8 | USA | | Equine | 1986 | | H3N8 | | PA | | CY030098 | |  |
|  | A/equine/Kentucky/1/1986/H3N8 | USA | | Equine | 1986 | | H3N8 | | HA | | CY030093 | |  |
|  | A/equine/Kentucky/1/1986/H3N8 | USA | | Equine | 1986 | | H3N8 | | NP | | CY030096 | |  |
|  | A/equine/Kentucky/1/1986/H3N8 | USA | | Equine | 1986 | | H3N8 | | NA | | CY030095 | |  |
|  | A/equine/Kentucky/1/1986/H3N8 | USA | | Equine | 1986 | | H3N8 | | MP | | CY030094 | |  |
|  | A/equine/Kentucky/1/1986/H3N8 | USA | | Equine | 1986 | | H3N8 | | NS | | CY030097 | |  |
|  | A/equine/Kentucky/2/1986/H3N8 | USA | | Equine | 1986 | | H3N8 | | PB2 | | CY032228 | |  |
|  | A/equine/Kentucky/2/1986/H3N8 | USA | | Equine | 1986 | | H3N8 | | PB1 | | CY032227 | |  |
|  | A/equine/Kentucky/2/1986/H3N8 | USA | | Equine | 1986 | | H3N8 | | PA | | CY032226 | |  |
|  | A/equine/Kentucky/2/1986/H3N8 | USA | | Equine | 1986 | | H3N8 | | HA | | CY032221 | |  |
|  | A/equine/Kentucky/2/1986/H3N8 | USA | | Equine | 1986 | | H3N8 | | NP | | CY032224 | |  |
|  | A/equine/Kentucky/2/1986/H3N8 | USA | | Equine | 1986 | | H3N8 | | NA | | CY032223 | |  |
|  | A/equine/Kentucky/2/1986/H3N8 | USA | | Equine | 1986 | | H3N8 | | MP | | CY032222 | |  |
|  | A/equine/Kentucky/2/1986/H3N8 | USA | | Equine | 1986 | | H3N8 | | NS | | CY032225 | |  |
|  | A/equine/Kentucky/3/1986/H3N8 | USA | | Equine | 1986 | | H3N8 | | PB2 | | CY032236 | |  |
|  | A/equine/Kentucky/3/1986/H3N8 | USA | | Equine | 1986 | | H3N8 | | PB1 | | CY032235 | |  |
|  | A/equine/Kentucky/3/1986/H3N8 | USA | | Equine | 1986 | | H3N8 | | PA | | CY032234 | |  |
|  | A/equine/Kentucky/3/1986/H3N8 | USA | | Equine | 1986 | | H3N8 | | HA | | CY032229 | |  |
|  | A/equine/Kentucky/3/1986/H3N8 | USA | | Equine | 1986 | | H3N8 | | NP | | CY032232 | |  |
|  | A/equine/Kentucky/3/1986/H3N8 | USA | | Equine | 1986 | | H3N8 | | NA | | CY032231 | |  |
|  | A/equine/Kentucky/3/1986/H3N8 | USA | | Equine | 1986 | | H3N8 | | MP | | CY032230 | |  |
|  | A/equine/Kentucky/3/1986/H3N8 | USA | | Equine | 1986 | | H3N8 | | NS | | CY032233 | |  |
|  | A/equine/Tennessee/5/1986/H3N8 | USA | | Equine | 1986 | | H3N8 | | PB2 | | CY030172 | |  |
|  | A/equine/Tennessee/5/1986/H3N8 | USA | | Equine | 1986 | | H3N8 | | PB1 | | CY030171 | |  |
|  | A/equine/Tennessee/5/1986/H3N8 | USA | | Equine | 1986 | | H3N8 | | PA | | CY030170 | |  |
|  | A/equine/Tennessee/5/1986/H3N8 | USA | | Equine | 1986 | | H3N8 | | HA | | CY030165 | |  |
|  | A/equine/Tennessee/5/1986/H3N8 | USA | | Equine | 1986 | | H3N8 | | NP | | CY030168 | |  |
|  | A/equine/Tennessee/5/1986/H3N8 | USA | | Equine | 1986 | | H3N8 | | NA | | CY030167 | |  |
|  | A/equine/Tennessee/5/1986/H3N8 | USA | | Equine | 1986 | | H3N8 | | MP | | CY030166 | |  |
|  | A/equine/Tennessee/5/1986/H3N8 | USA | | Equine | 1986 | | H3N8 | | NS | | CY030169 | |  |
|  | A/equine/Cordoba/18/1985/H3N8 | Argentina | | Equine | 1985 | | H3N8 | | PB2 | | CY032308 | |  |
|  | A/equine/Cordoba/18/1985/H3N8 | Argentina | | Equine | 1985 | | H3N8 | | PB1 | | CY032307 | |  |
|  | A/equine/Cordoba/18/1985/H3N8 | Argentina | | Equine | 1985 | | H3N8 | | PA | | CY032306 | |  |
|  | A/equine/Cordoba/18/1985/H3N8 | Argentina | | Equine | 1985 | | H3N8 | | HA | | CY032301 | |  |
|  | A/equine/Cordoba/18/1985/H3N8 | Argentina | | Equine | 1985 | | H3N8 | | NP | | CY032304 | |  |
|  | A/equine/Cordoba/18/1985/H3N8 | Argentina | | Equine | 1985 | | H3N8 | | NA | | CY032303 | |  |
|  | A/equine/Cordoba/18/1985/H3N8 | Argentina | | Equine | 1985 | | H3N8 | | MP | | CY032302 | |  |
|  | A/equine/Cordoba/18/1985/H3N8 | Argentina | | Equine | 1985 | | H3N8 | | NS | | CY032305 | |  |
|  | A/equine/Santa Fe/1/1985/H3N8 | USA | | Equine | 1985 | | H3N8 | | PB2 | | CY032316 | |  |
|  | A/equine/Santa Fe/1/1985/H3N8 | USA | | Equine | 1985 | | H3N8 | | PB1 | | CY032315 | |  |
|  | A/equine/Santa Fe/1/1985/H3N8 | USA | | Equine | 1985 | | H3N8 | | PA | | CY032314 | |  |
|  | A/equine/Santa Fe/1/1985/H3N8 | USA | | Equine | 1985 | | H3N8 | | HA | | CY032309 | |  |
|  | A/equine/Santa Fe/1/1985/H3N8 | USA | | Equine | 1985 | | H3N8 | | NP | | CY032312 | |  |
|  | A/equine/Santa Fe/1/1985/H3N8 | USA | | Equine | 1985 | | H3N8 | | NA | | CY032311 | |  |
|  | A/equine/Santa Fe/1/1985/H3N8 | USA | | Equine | 1985 | | H3N8 | | MP | | CY032310 | |  |
|  | A/equine/Santa Fe/1/1985/H3N8 | USA | | Equine | 1985 | | H3N8 | | NS | | CY032313 | |  |
|  | A/equine/New York/VR-297/1983/H3N8 | USA | | Equine | 1983 | | H3N8 | | PB2 | | CY028923 | |  |
|  | A/equine/New York/VR-297/1983/H3N8 | USA | | Equine | 1983 | | H3N8 | | PB1 | | CY028922 | |  |
|  | A/equine/New York/VR-297/1983/H3N8 | USA | | Equine | 1983 | | H3N8 | | PA | | CY028921 | |  |
|  | A/equine/New York/VR-297/1983/H3N8 | USA | | Equine | 1983 | | H3N8 | | HA | | CY028916 | |  |
|  | A/equine/New York/VR-297/1983/H3N8 | USA | | Equine | 1983 | | H3N8 | | NP | | CY028919 | |  |
|  | A/equine/New York/VR-297/1983/H3N8 | USA | | Equine | 1983 | | H3N8 | | NA | | CY028918 | |  |
|  | A/equine/New York/VR-297/1983/H3N8 | USA | | Equine | 1983 | | H3N8 | | MP | | CY028917 | |  |
|  | A/equine/New York/VR-297/1983/H3N8 | USA | | Equine | 1983 | | H3N8 | | NS | | CY028920 | |  |
|  | A/equine/California/103/1982/H3N8 | USA | | Equine | 1982 | | H3N8 | | PB2 | | CY028915 | |  |
|  | A/equine/California/103/1982/H3N8 | USA | | Equine | 1982 | | H3N8 | | PB1 | | CY028914 | |  |
|  | A/equine/California/103/1982/H3N8 | USA | | Equine | 1982 | | H3N8 | | PA | | CY028913 | |  |
|  | A/equine/California/103/1982/H3N8 | USA | | Equine | 1982 | | H3N8 | | HA | | CY028908 | |  |
|  | A/equine/California/103/1982/H3N8 | USA | | Equine | 1982 | | H3N8 | | NP | | CY028911 | |  |
|  | A/equine/California/103/1982/H3N8 | USA | | Equine | 1982 | | H3N8 | | NA | | CY028910 | |  |
|  | A/equine/California/103/1982/H3N8 | USA | | Equine | 1982 | | H3N8 | | MP | | CY028909 | |  |
|  | A/equine/California/103/1982/H3N8 | USA | | Equine | 1982 | | H3N8 | | NS | | CY028912 | |  |
|  | A/equine/California/83/1982/H3N8 | USA | | Equine | 1982 | | H3N8 | | PB2 | | CY030092 | |  |
|  | A/equine/California/83/1982/H3N8 | USA | | Equine | 1982 | | H3N8 | | PB1 | | CY030091 | |  |
|  | A/equine/California/83/1982/H3N8 | USA | | Equine | 1982 | | H3N8 | | PA | | CY030090 | |  |
|  | A/equine/California/83/1982/H3N8 | USA | | Equine | 1982 | | H3N8 | | HA | | CY030085 | |  |
|  | A/equine/California/83/1982/H3N8 | USA | | Equine | 1982 | | H3N8 | | NP | | CY030088 | |  |
|  | A/equine/California/83/1982/H3N8 | USA | | Equine | 1982 | | H3N8 | | NA | | CY030087 | |  |
|  | A/equine/California/83/1982/H3N8 | USA | | Equine | 1982 | | H3N8 | | MP | | CY030086 | |  |
|  | A/equine/California/83/1982/H3N8 | USA | | Equine | 1982 | | H3N8 | | NS | | CY030089 | |  |
|  | A/equine/Georgia/1/1981/H3N8 | USA | | Equine | 1981 | | H3N8 | | PB2 | | CY028867 | |  |
|  | A/equine/Georgia/1/1981/H3N8 | USA | | Equine | 1981 | | H3N8 | | PB1 | | CY028866 | |  |
|  | A/equine/Georgia/1/1981/H3N8 | USA | | Equine | 1981 | | H3N8 | | PA | | CY028865 | |  |
|  | A/equine/Georgia/1/1981/H3N8 | USA | | Equine | 1981 | | H3N8 | | HA | | CY028860 | |  |
|  | A/equine/Georgia/1/1981/H3N8 | USA | | Equine | 1981 | | H3N8 | | NP | | CY028863 | |  |
|  | A/equine/Georgia/1/1981/H3N8 | USA | | Equine | 1981 | | H3N8 | | NA | | CY028862 | |  |
|  | A/equine/Georgia/1/1981/H3N8 | USA | | Equine | 1981 | | H3N8 | | MP | | CY028861 | |  |
|  | A/equine/Georgia/1/1981/H3N8 | USA | | Equine | 1981 | | H3N8 | | NS | | CY028864 | |  |
|  | A/equine/Georgia/10/1981/H3N8 | USA | | Equine | 1981 | | H3N8 | | PB2 | | CY028891 | |  |
|  | A/equine/Georgia/10/1981/H3N8 | USA | | Equine | 1981 | | H3N8 | | PB1 | | CY028890 | |  |
|  | A/equine/Georgia/10/1981/H3N8 | USA | | Equine | 1981 | | H3N8 | | PA | | CY028889 | |  |
|  | A/equine/Georgia/10/1981/H3N8 | USA | | Equine | 1981 | | H3N8 | | HA | | CY028884 | |  |
|  | A/equine/Georgia/10/1981/H3N8 | USA | | Equine | 1981 | | H3N8 | | NP | | CY028887 | |  |
|  | A/equine/Georgia/10/1981/H3N8 | USA | | Equine | 1981 | | H3N8 | | NA | | CY028886 | |  |
|  | A/equine/Georgia/10/1981/H3N8 | USA | | Equine | 1981 | | H3N8 | | MP | | CY028885 | |  |
|  | A/equine/Georgia/10/1981/H3N8 | USA | | Equine | 1981 | | H3N8 | | NS | | CY028888 | |  |
|  | A/equine/Georgia/13/1981/H3N8 | USA | | Equine | 1981 | | H3N8 | | PB2 | | CY028899 | |  |
|  | A/equine/Georgia/13/1981/H3N8 | USA | | Equine | 1981 | | H3N8 | | PB1 | | CY028898 | |  |
|  | A/equine/Georgia/13/1981/H3N8 | USA | | Equine | 1981 | | H3N8 | | PA | | CY028897 | |  |
|  | A/equine/Georgia/13/1981/H3N8 | USA | | Equine | 1981 | | H3N8 | | HA | | CY028892 | |  |
|  | A/equine/Georgia/13/1981/H3N8 | USA | | Equine | 1981 | | H3N8 | | NP | | CY028895 | |  |
|  | A/equine/Georgia/13/1981/H3N8 | USA | | Equine | 1981 | | H3N8 | | NA | | CY028894 | |  |
|  | A/equine/Georgia/13/1981/H3N8 | USA | | Equine | 1981 | | H3N8 | | MP | | CY028893 | |  |
|  | A/equine/Georgia/13/1981/H3N8 | USA | | Equine | 1981 | | H3N8 | | NS | | CY028896 | |  |
|  | A/equine/Georgia/3/1981/H3N8 | USA | | Equine | 1981 | | H3N8 | | PB2 | | CY028875 | |  |
|  | A/equine/Georgia/3/1981/H3N8 | USA | | Equine | 1981 | | H3N8 | | PB1 | | CY028874 | |  |
|  | A/equine/Georgia/3/1981/H3N8 | USA | | Equine | 1981 | | H3N8 | | PA | | CY028873 | |  |
|  | A/equine/Georgia/3/1981/H3N8 | USA | | Equine | 1981 | | H3N8 | | HA | | CY028868 | |  |
|  | A/equine/Georgia/3/1981/H3N8 | USA | | Equine | 1981 | | H3N8 | | NP | | CY028871 | |  |
|  | A/equine/Georgia/3/1981/H3N8 | USA | | Equine | 1981 | | H3N8 | | NA | | CY028870 | |  |
|  | A/equine/Georgia/3/1981/H3N8 | USA | | Equine | 1981 | | H3N8 | | MP | | CY028869 | |  |
|  | A/equine/Georgia/3/1981/H3N8 | USA | | Equine | 1981 | | H3N8 | | NS | | CY028872 | |  |
|  | A/equine/Georgia/9/1981/H3N8 | USA | | Equine | 1981 | | H3N8 | | PB2 | | CY028883 | |  |
|  | A/equine/Georgia/9/1981/H3N8 | USA | | Equine | 1981 | | H3N8 | | PB1 | | CY028882 | |  |
|  | A/equine/Georgia/9/1981/H3N8 | USA | | Equine | 1981 | | H3N8 | | PA | | CY028881 | |  |
|  | A/equine/Georgia/9/1981/H3N8 | USA | | Equine | 1981 | | H3N8 | | HA | | CY028876 | |  |
|  | A/equine/Georgia/9/1981/H3N8 | USA | | Equine | 1981 | | H3N8 | | NP | | CY028879 | |  |
|  | A/equine/Georgia/9/1981/H3N8 | USA | | Equine | 1981 | | H3N8 | | NA | | CY028878 | |  |
|  | A/equine/Georgia/9/1981/H3N8 | USA | | Equine | 1981 | | H3N8 | | MP | | CY028877 | |  |
|  | A/equine/Georgia/9/1981/H3N8 | USA | | Equine | 1981 | | H3N8 | | NS | | CY028880 | |  |
|  | A/equine/Kentucky/1/1981/H3N8 | USA | | Equine | 1981 | | H3N8 | | PB2 | | CY028835 | |  |
|  | A/equine/Kentucky/1/1981/H3N8 | USA | | Equine | 1981 | | H3N8 | | PB1 | | CY028834 | |  |
|  | A/equine/Kentucky/1/1981/H3N8 | USA | | Equine | 1981 | | H3N8 | | PA | | CY028833 | |  |
|  | A/equine/Kentucky/1/1981/H3N8 | USA | | Equine | 1981 | | H3N8 | | HA | | CY028828 | |  |
|  | A/equine/Kentucky/1/1981/H3N8 | USA | | Equine | 1981 | | H3N8 | | NP | | CY028831 | |  |
|  | A/equine/Kentucky/1/1981/H3N8 | USA | | Equine | 1981 | | H3N8 | | NA | | CY028830 | |  |
|  | A/equine/Kentucky/1/1981/H3N8 | USA | | Equine | 1981 | | H3N8 | | MP | | CY028829 | |  |
|  | A/equine/Kentucky/1/1981/H3N8 | USA | | Equine | 1981 | | H3N8 | | NS | | CY028832 | |  |
|  | A/equine/Kentucky/2/1981/H3N8 | USA | | Equine | 1981 | | H3N8 | | PB2 | | CY028827 | |  |
|  | A/equine/Kentucky/2/1981/H3N8 | USA | | Equine | 1981 | | H3N8 | | PB1 | | CY028826 | |  |
|  | A/equine/Kentucky/2/1981/H3N8 | USA | | Equine | 1981 | | H3N8 | | PA | | CY028825 | |  |
|  | A/equine/Kentucky/2/1981/H3N8 | USA | | Equine | 1981 | | H3N8 | | HA | | CY028820 | |  |
|  | A/equine/Kentucky/2/1981/H3N8 | USA | | Equine | 1981 | | H3N8 | | NP | | CY028823 | |  |
|  | A/equine/Kentucky/2/1981/H3N8 | USA | | Equine | 1981 | | H3N8 | | NA | | CY028822 | |  |
|  | A/equine/Kentucky/2/1981/H3N8 | USA | | Equine | 1981 | | H3N8 | | MP | | CY028821 | |  |
|  | A/equine/Kentucky/2/1981/H3N8 | USA | | Equine | 1981 | | H3N8 | | NS | | CY028824 | |  |
|  | A/equine/Kentucky/3/1981/H3N8 | USA | | Equine | 1981 | | H3N8 | | PB2 | | CY030084 | |  |
|  | A/equine/Kentucky/3/1981/H3N8 | USA | | Equine | 1981 | | H3N8 | | PB1 | | CY030083 | |  |
|  | A/equine/Kentucky/3/1981/H3N8 | USA | | Equine | 1981 | | H3N8 | | PA | | CY030082 | |  |
|  | A/equine/Kentucky/3/1981/H3N8 | USA | | Equine | 1981 | | H3N8 | | HA | | CY030077 | |  |
|  | A/equine/Kentucky/3/1981/H3N8 | USA | | Equine | 1981 | | H3N8 | | NP | | CY030080 | |  |
|  | A/equine/Kentucky/3/1981/H3N8 | USA | | Equine | 1981 | | H3N8 | | NA | | CY030079 | |  |
|  | A/equine/Kentucky/3/1981/H3N8 | USA | | Equine | 1981 | | H3N8 | | MP | | CY030078 | |  |
|  | A/equine/Kentucky/3/1981/H3N8 | USA | | Equine | 1981 | | H3N8 | | NS | | CY030081 | |  |
|  | A/equine/Kentucky/Rosie100/1981/H3N8 | USA | | Equine | 1981 | | H3N8 | | PB2 | | CY030750 | |  |
|  | A/equine/Kentucky/Rosie100/1981/H3N8 | USA | | Equine | 1981 | | H3N8 | | PB1 | | CY030749 | |  |
|  | A/equine/Kentucky/Rosie100/1981/H3N8 | USA | | Equine | 1981 | | H3N8 | | PA | | CY030748 | |  |
|  | A/equine/Kentucky/Rosie100/1981/H3N8 | USA | | Equine | 1981 | | H3N8 | | HA | | CY030743 | |  |
|  | A/equine/Kentucky/Rosie100/1981/H3N8 | USA | | Equine | 1981 | | H3N8 | | NP | | CY030746 | |  |
|  | A/equine/Kentucky/Rosie100/1981/H3N8 | USA | | Equine | 1981 | | H3N8 | | NA | | CY030745 | |  |
|  | A/equine/Kentucky/Rosie100/1981/H3N8 | USA | | Equine | 1981 | | H3N8 | | MP | | CY030744 | |  |
|  | A/equine/Kentucky/Rosie100/1981/H3N8 | USA | | Equine | 1981 | | H3N8 | | NS | | CY030747 | |  |
|  | A/equine/Kentucky/magnificent_genius1/1981/H3N8 | USA | | Equine | 1981 | | H3N8 | | PB2 | | CY028907 | |  |
|  | A/equine/Kentucky/magnificent_genius1/1981/H3N8 | USA | | Equine | 1981 | | H3N8 | | PB1 | | CY028906 | |  |
|  | A/equine/Kentucky/magnificent_genius1/1981/H3N8 | USA | | Equine | 1981 | | H3N8 | | PA | | CY028905 | |  |
|  | A/equine/Kentucky/magnificent_genius1/1981/H3N8 | USA | | Equine | 1981 | | H3N8 | | HA | | CY028900 | |  |
|  | A/equine/Kentucky/magnificent_genius1/1981/H3N8 | USA | | Equine | 1981 | | H3N8 | | NP | | CY028903 | |  |
|  | A/equine/Kentucky/magnificent_genius1/1981/H3N8 | USA | | Equine | 1981 | | H3N8 | | NA | | CY028902 | |  |
|  | A/equine/Kentucky/magnificent_genius1/1981/H3N8 | USA | | Equine | 1981 | | H3N8 | | MP | | CY028901 | |  |
|  | A/equine/Kentucky/magnificent_genius1/1981/H3N8 | USA | | Equine | 1981 | | H3N8 | | NS | | CY028904 | |  |
|  | A/equine/California/1/1980/H3N8 | USA | | Equine | 1980 | | H3N8 | | PB2 | | CY028819 | |  |
|  | A/equine/California/1/1980/H3N8 | USA | | Equine | 1980 | | H3N8 | | PB1 | | CY028818 | |  |
|  | A/equine/California/1/1980/H3N8 | USA | | Equine | 1980 | | H3N8 | | PA | | CY028817 | |  |
|  | A/equine/California/1/1980/H3N8 | USA | | Equine | 1980 | | H3N8 | | HA | | CY028812 | |  |
|  | A/equine/California/1/1980/H3N8 | USA | | Equine | 1980 | | H3N8 | | NP | | CY028815 | |  |
|  | A/equine/California/1/1980/H3N8 | USA | | Equine | 1980 | | H3N8 | | NA | | CY028814 | |  |
|  | A/equine/California/1/1980/H3N8 | USA | | Equine | 1980 | | H3N8 | | MP | | CY028813 | |  |
|  | A/equine/California/1/1980/H3N8 | USA | | Equine | 1980 | | H3N8 | | NS | | CY028816 | |  |
|  | A/equine/Kentucky/2/1980/H3N8 | USA | | Equine | 1980 | | H3N8 | | PB2 | | CY032944 | |  |
|  | A/equine/Kentucky/2/1980/H3N8 | USA | | Equine | 1980 | | H3N8 | | PB1 | | CY032943 | |  |
|  | A/equine/Kentucky/2/1980/H3N8 | USA | | Equine | 1980 | | H3N8 | | PA | | CY032942 | |  |
|  | A/equine/Kentucky/2/1980/H3N8 | USA | | Equine | 1980 | | H3N8 | | HA | | CY032937 | |  |
|  | A/equine/Kentucky/2/1980/H3N8 | USA | | Equine | 1980 | | H3N8 | | NP | | CY032940 | |  |
|  | A/equine/Kentucky/2/1980/H3N8 | USA | | Equine | 1980 | | H3N8 | | NA | | CY032939 | |  |
|  | A/equine/Kentucky/2/1980/H3N8 | USA | | Equine | 1980 | | H3N8 | | MP | | CY032938 | |  |
|  | A/equine/Kentucky/2/1980/H3N8 | USA | | Equine | 1980 | | H3N8 | | NS | | CY032941 | |  |
|  | A/equine/Kentucky/4/1980/H3N8 | USA | | Equine | 1980 | | H3N8 | | PB2 | | CY028811 | |  |
|  | A/equine/Kentucky/4/1980/H3N8 | USA | | Equine | 1980 | | H3N8 | | PB1 | | CY028810 | |  |
|  | A/equine/Kentucky/4/1980/H3N8 | USA | | Equine | 1980 | | H3N8 | | PA | | CY028809 | |  |
|  | A/equine/Kentucky/4/1980/H3N8 | USA | | Equine | 1980 | | H3N8 | | HA | | CY028804 | |  |
|  | A/equine/Kentucky/4/1980/H3N8 | USA | | Equine | 1980 | | H3N8 | | NP | | CY028807 | |  |
|  | A/equine/Kentucky/4/1980/H3N8 | USA | | Equine | 1980 | | H3N8 | | NA | | CY028806 | |  |
|  | A/equine/Kentucky/4/1980/H3N8 | USA | | Equine | 1980 | | H3N8 | | MP | | CY028805 | |  |
|  | A/equine/Kentucky/4/1980/H3N8 | USA | | Equine | 1980 | | H3N8 | | NS | | CY028808 | |  |
|  | A/equine/Romania/1/1980/H3N8 | Romania | | Equine | 1980 | | H3N8 | | PB2 | | CY032396 | |  |
|  | A/equine/Romania/1/1980/H3N8 | Romania | | Equine | 1980 | | H3N8 | | PB1 | | CY032395 | |  |
|  | A/equine/Romania/1/1980/H3N8 | Romania | | Equine | 1980 | | H3N8 | | PA | | CY032394 | |  |
|  | A/equine/Romania/1/1980/H3N8 | Romania | | Equine | 1980 | | H3N8 | | HA | | CY032389 | |  |
|  | A/equine/Romania/1/1980/H3N8 | Romania | | Equine | 1980 | | H3N8 | | NP | | CY032392 | |  |
|  | A/equine/Romania/1/1980/H3N8 | Romania | | Equine | 1980 | | H3N8 | | NA | | CY032391 | |  |
|  | A/equine/Romania/1/1980/H3N8 | Romania | | Equine | 1980 | | H3N8 | | MP | | CY032390 | |  |
|  | A/equine/Romania/1/1980/H3N8 | Romania | | Equine | 1980 | | H3N8 | | NS | | CY032393 | |  |
|  | A/equine/Fontainebleau/1/1979/H3N8 | France | | Equine | 1979 | | H3N8 | | PB2 | | CY032412 | |  |
|  | A/equine/Fontainebleau/1/1979/H3N8 | France | | Equine | 1979 | | H3N8 | | PB1 | | CY032411 | |  |
|  | A/equine/Fontainebleau/1/1979/H3N8 | France | | Equine | 1979 | | H3N8 | | PA | | CY032410 | |  |
|  | A/equine/Fontainebleau/1/1979/H3N8 | France | | Equine | 1979 | | H3N8 | | HA | | CY032405 | |  |
|  | A/equine/Fontainebleau/1/1979/H3N8 | France | | Equine | 1979 | | H3N8 | | NP | | CY032408 | |  |
|  | A/equine/Fontainebleau/1/1979/H3N8 | France | | Equine | 1979 | | H3N8 | | NA | | CY032407 | |  |
|  | A/equine/Fontainebleau/1/1979/H3N8 | France | | Equine | 1979 | | H3N8 | | MP | | CY032406 | |  |
|  | A/equine/Fontainebleau/1/1979/H3N8 | France | | Equine | 1979 | | H3N8 | | NS | | CY032409 | |  |
|  | A/equine/New Market/1/1979/H3N8 | USA | | Equine | 1979 | | H3N8 | | PB2 | | CY096898 | |  |
|  | A/equine/New Market/1/1979/H3N8 | USA | | Equine | 1979 | | H3N8 | | PB1 | | CY096897 | |  |
|  | A/equine/New Market/1/1979/H3N8 | USA | | Equine | 1979 | | H3N8 | | PA | | CY096896 | |  |
|  | A/equine/New Market/1/1979/H3N8 | USA | | Equine | 1979 | | H3N8 | | HA | | CY096891 | |  |
|  | A/equine/New Market/1/1979/H3N8 | USA | | Equine | 1979 | | H3N8 | | NP | | CY096894 | |  |
|  | A/equine/New Market/1/1979/H3N8 | USA | | Equine | 1979 | | H3N8 | | NA | | CY096893 | |  |
|  | A/equine/New Market/1/1979/H3N8 | USA | | Equine | 1979 | | H3N8 | | MP | | CY096892 | |  |
|  | A/equine/New Market/1/1979/H3N8 | USA | | Equine | 1979 | | H3N8 | | NS | | CY096895 | |  |
|  | A/equine/New Market/nasalwash1/1979/H3N8 | USA | | Equine | 1979 | | H3N8 | | PB2 | | CY096906 | |  |
|  | A/equine/New Market/nasalwash1/1979/H3N8 | USA | | Equine | 1979 | | H3N8 | | PB1 | | CY096905 | |  |
|  | A/equine/New Market/nasalwash1/1979/H3N8 | USA | | Equine | 1979 | | H3N8 | | PA | | CY096904 | |  |
|  | A/equine/New Market/nasalwash1/1979/H3N8 | USA | | Equine | 1979 | | H3N8 | | HA | | CY096899 | |  |
|  | A/equine/New Market/nasalwash1/1979/H3N8 | USA | | Equine | 1979 | | H3N8 | | NP | | CY096902 | |  |
|  | A/equine/New Market/nasalwash1/1979/H3N8 | USA | | Equine | 1979 | | H3N8 | | NA | | CY096901 | |  |
|  | A/equine/New Market/nasalwash1/1979/H3N8 | USA | | Equine | 1979 | | H3N8 | | MP | | CY096900 | |  |
|  | A/equine/New Market/nasalwash1/1979/H3N8 | USA | | Equine | 1979 | | H3N8 | | NS | | CY096903 | |  |
|  | A/equine/Switzerland/1118/1979/H3N8 | Switzerland | | Equine | 1979 | | H3N8 | | PB2 | | CY032388 | |  |
|  | A/equine/Switzerland/1118/1979/H3N8 | Switzerland | | Equine | 1979 | | H3N8 | | PB1 | | CY032387 | |  |
|  | A/equine/Switzerland/1118/1979/H3N8 | Switzerland | | Equine | 1979 | | H3N8 | | PA | | CY032386 | |  |
|  | A/equine/Switzerland/1118/1979/H3N8 | Switzerland | | Equine | 1979 | | H3N8 | | HA | | CY032381 | |  |
|  | A/equine/Switzerland/1118/1979/H3N8 | Switzerland | | Equine | 1979 | | H3N8 | | NP | | CY032384 | |  |
|  | A/equine/Switzerland/1118/1979/H3N8 | Switzerland | | Equine | 1979 | | H3N8 | | NA | | CY032383 | |  |
|  | A/equine/Switzerland/1118/1979/H3N8 | Switzerland | | Equine | 1979 | | H3N8 | | MP | | CY032382 | |  |
|  | A/equine/Switzerland/1118/1979/H3N8 | Switzerland | | Equine | 1979 | | H3N8 | | NS | | CY032385 | |  |
|  | A/equine/Kascakew/1/1978/H3N8 | unknown | | Equine | 1978 | | H3N8 | | PB2 | | CY033488 | |  |
|  | A/equine/Kascakew/1/1978/H3N8 | unknown | | Equine | 1978 | | H3N8 | | PB1 | | CY033487 | |  |
|  | A/equine/Kascakew/1/1978/H3N8 | unknown | | Equine | 1978 | | H3N8 | | PA | | CY033486 | |  |
|  | A/equine/Kascakew/1/1978/H3N8 | unknown | | Equine | 1978 | | H3N8 | | HA | | CY033481 | |  |
|  | A/equine/Kascakew/1/1978/H3N8 | unknown | | Equine | 1978 | | H3N8 | | NP | | CY033484 | |  |
|  | A/equine/Kascakew/1/1978/H3N8 | unknown | | Equine | 1978 | | H3N8 | | NA | | CY033483 | |  |
|  | A/equine/Kascakew/1/1978/H3N8 | unknown | | Equine | 1978 | | H3N8 | | MP | | CY033482 | |  |
|  | A/equine/Kascakew/1/1978/H3N8 | unknown | | Equine | 1978 | | H3N8 | | NS | | CY033485 | |  |
|  | A/equine/Kentucky/1/1978/H3N8 | USA | | Equine | 1978 | | H3N8 | | PB2 | | CY028803 | |  |
|  | A/equine/Kentucky/1/1978/H3N8 | USA | | Equine | 1978 | | H3N8 | | PB1 | | CY028802 | |  |
|  | A/equine/Kentucky/1/1978/H3N8 | USA | | Equine | 1978 | | H3N8 | | PA | | CY028801 | |  |
|  | A/equine/Kentucky/1/1978/H3N8 | USA | | Equine | 1978 | | H3N8 | | HA | | CY028796 | |  |
|  | A/equine/Kentucky/1/1978/H3N8 | USA | | Equine | 1978 | | H3N8 | | NP | | CY028799 | |  |
|  | A/equine/Kentucky/1/1978/H3N8 | USA | | Equine | 1978 | | H3N8 | | NA | | CY028798 | |  |
|  | A/equine/Kentucky/1/1978/H3N8 | USA | | Equine | 1978 | | H3N8 | | MP | | CY028797 | |  |
|  | A/equine/Kentucky/1/1978/H3N8 | USA | | Equine | 1978 | | H3N8 | | NS | | CY028800 | |  |
|  | A/equine/Argentina/1/1977/H7N7 | Argentina | | Equine | 1977 | | H7N7 | | PB2 | | CY036902 | |  |
|  | A/equine/Argentina/1/1977/H7N7 | Argentina | | Equine | 1977 | | H7N7 | | PB1 | | CY036901 | |  |
|  | A/equine/Argentina/1/1977/H7N7 | Argentina | | Equine | 1977 | | H7N7 | | PA | | CY036900 | |  |
|  | A/equine/Argentina/1/1977/H7N7 | Argentina | | Equine | 1977 | | H7N7 | | HA | | CY036895 | |  |
|  | A/equine/Argentina/1/1977/H7N7 | Argentina | | Equine | 1977 | | H7N7 | | NP | | CY036898 | |  |
|  | A/equine/Argentina/1/1977/H7N7 | Argentina | | Equine | 1977 | | H7N7 | | NA | | CY036897 | |  |
|  | A/equine/Argentina/1/1977/H7N7 | Argentina | | Equine | 1977 | | H7N7 | | MP | | CY036896 | |  |
|  | A/equine/Argentina/1/1977/H7N7 | Argentina | | Equine | 1977 | | H7N7 | | NS | | CY036899 | |  |
|  | A/equine/Kentucky/bitter_boredom5/1976/H3N8 | USA | | Equine | 1976 | | H3N8 | | PB2 | | CY028859 | |  |
|  | A/equine/Kentucky/bitter_boredom5/1976/H3N8 | USA | | Equine | 1976 | | H3N8 | | PB1 | | CY028858 | |  |
|  | A/equine/Kentucky/bitter_boredom5/1976/H3N8 | USA | | Equine | 1976 | | H3N8 | | PA | | CY028857 | |  |
|  | A/equine/Kentucky/bitter_boredom5/1976/H3N8 | USA | | Equine | 1976 | | H3N8 | | HA | | CY028852 | |  |
|  | A/equine/Kentucky/bitter_boredom5/1976/H3N8 | USA | | Equine | 1976 | | H3N8 | | NP | | CY028855 | |  |
|  | A/equine/Kentucky/bitter_boredom5/1976/H3N8 | USA | | Equine | 1976 | | H3N8 | | NA | | CY028854 | |  |
|  | A/equine/Kentucky/bitter_boredom5/1976/H3N8 | USA | | Equine | 1976 | | H3N8 | | MP | | CY028853 | |  |
|  | A/equine/Kentucky/bitter_boredom5/1976/H3N8 | USA | | Equine | 1976 | | H3N8 | | NS | | CY028856 | |  |
|  | A/equine/Kentucky/pass_the_pepper1/1976/H3N8 | USA | | Equine | 1976 | | H3N8 | | PB2 | | CY028851 | |  |
|  | A/equine/Kentucky/pass_the_pepper1/1976/H3N8 | USA | | Equine | 1976 | | H3N8 | | PB1 | | CY028850 | |  |
|  | A/equine/Kentucky/pass_the_pepper1/1976/H3N8 | USA | | Equine | 1976 | | H3N8 | | PA | | CY028849 | |  |
|  | A/equine/Kentucky/pass_the_pepper1/1976/H3N8 | USA | | Equine | 1976 | | H3N8 | | HA | | CY028844 | |  |
|  | A/equine/Kentucky/pass_the_pepper1/1976/H3N8 | USA | | Equine | 1976 | | H3N8 | | NP | | CY028847 | |  |
|  | A/equine/Kentucky/pass_the_pepper1/1976/H3N8 | USA | | Equine | 1976 | | H3N8 | | NA | | CY028846 | |  |
|  | A/equine/Kentucky/pass_the_pepper1/1976/H3N8 | USA | | Equine | 1976 | | H3N8 | | MP | | CY028845 | |  |
|  | A/equine/Kentucky/pass_the_pepper1/1976/H3N8 | USA | | Equine | 1976 | | H3N8 | | NS | | CY028848 | |  |
|  | A/equine/Sao Paulo/4/1976/H7N7 | Brazil | | Equine | 1976 | | H7N7 | | PB2 | | CY036886 | |  |
|  | A/equine/Sao Paulo/4/1976/H7N7 | Brazil | | Equine | 1976 | | H7N7 | | PB1 | | CY036885 | |  |
|  | A/equine/Sao Paulo/4/1976/H7N7 | Brazil | | Equine | 1976 | | H7N7 | | PA | | CY036884 | |  |
|  | A/equine/Sao Paulo/4/1976/H7N7 | Brazil | | Equine | 1976 | | H7N7 | | HA | | CY036879 | |  |
|  | A/equine/Sao Paulo/4/1976/H7N7 | Brazil | | Equine | 1976 | | H7N7 | | NP | | CY036882 | |  |
|  | A/equine/Sao Paulo/4/1976/H7N7 | Brazil | | Equine | 1976 | | H7N7 | | NA | | CY036881 | |  |
|  | A/equine/Sao Paulo/4/1976/H7N7 | Brazil | | Equine | 1976 | | H7N7 | | MP | | CY036880 | |  |
|  | A/equine/Sao Paulo/4/1976/H7N7 | Brazil | | Equine | 1976 | | H7N7 | | NS | | CY036883 | |  |
|  | A/equine/Uruguay/1063/1976/H7N7 | Uruguay | | Equine | 1976 | | H7N7 | | PB2 | | CY036894 | |  |
|  | A/equine/Uruguay/1063/1976/H7N7 | Uruguay | | Equine | 1976 | | H7N7 | | PB1 | | CY036893 | |  |
|  | A/equine/Uruguay/1063/1976/H7N7 | Uruguay | | Equine | 1976 | | H7N7 | | PA | | CY036892 | |  |
|  | A/equine/Uruguay/1063/1976/H7N7 | Uruguay | | Equine | 1976 | | H7N7 | | HA | | CY036887 | |  |
|  | A/equine/Uruguay/1063/1976/H7N7 | Uruguay | | Equine | 1976 | | H7N7 | | NP | | CY036890 | |  |
|  | A/equine/Uruguay/1063/1976/H7N7 | Uruguay | | Equine | 1976 | | H7N7 | | NA | | CY036889 | |  |
|  | A/equine/Uruguay/1063/1976/H7N7 | Uruguay | | Equine | 1976 | | H7N7 | | MP | | CY036888 | |  |
|  | A/equine/Uruguay/1063/1976/H7N7 | Uruguay | | Equine | 1976 | | H7N7 | | NS | | CY036891 | |  |
|  | A/equine/Kentucky/1a/1975/H7N7 | USA | | Equine | 1975 | | H7N7 | | PB2 | | CY036878 | |  |
|  | A/equine/Kentucky/1a/1975/H7N7 | USA | | Equine | 1975 | | H7N7 | | PB1 | | CY036877 | |  |
|  | A/equine/Kentucky/1a/1975/H7N7 | USA | | Equine | 1975 | | H7N7 | | PA | | CY036876 | |  |
|  | A/equine/Kentucky/1a/1975/H7N7 | USA | | Equine | 1975 | | H7N7 | | HA | | CY036871 | |  |
|  | A/equine/Kentucky/1a/1975/H7N7 | USA | | Equine | 1975 | | H7N7 | | NP | | CY036874 | |  |
|  | A/equine/Kentucky/1a/1975/H7N7 | USA | | Equine | 1975 | | H7N7 | | NA | | CY036873 | |  |
|  | A/equine/Kentucky/1a/1975/H7N7 | USA | | Equine | 1975 | | H7N7 | | MP | | CY036872 | |  |
|  | A/equine/Kentucky/1a/1975/H7N7 | USA | | Equine | 1975 | | H7N7 | | NS | | CY036875 | |  |
|  | A/equine/New York/1/1975/H3N8 | USA | | Equine | 1975 | | H3N8 | | PB2 | | CY030196 | |  |
|  | A/equine/New York/1/1975/H3N8 | USA | | Equine | 1975 | | H3N8 | | PB1 | | CY030195 | |  |
|  | A/equine/New York/1/1975/H3N8 | USA | | Equine | 1975 | | H3N8 | | PA | | CY030194 | |  |
|  | A/equine/New York/1/1975/H3N8 | USA | | Equine | 1975 | | H3N8 | | HA | | CY030189 | |  |
|  | A/equine/New York/1/1975/H3N8 | USA | | Equine | 1975 | | H3N8 | | NP | | CY030192 | |  |
|  | A/equine/New York/1/1975/H3N8 | USA | | Equine | 1975 | | H3N8 | | NA | | CY030191 | |  |
|  | A/equine/New York/1/1975/H3N8 | USA | | Equine | 1975 | | H3N8 | | MP | | CY030190 | |  |
|  | A/equine/New York/1/1975/H3N8 | USA | | Equine | 1975 | | H3N8 | | NS | | CY030193 | |  |
|  | A/equine/Sachiyama/1/1971/H3N8 | Japan | | Equine | 1971 | | H3N8 | | PB2 | | CY034941 | |  |
|  | A/equine/Sachiyama/1/1971/H3N8 | Japan | | Equine | 1971 | | H3N8 | | PB1 | | CY034940 | |  |
|  | A/equine/Sachiyama/1/1971/H3N8 | Japan | | Equine | 1971 | | H3N8 | | PA | | CY034939 | |  |
|  | A/equine/Sachiyama/1/1971/H3N8 | Japan | | Equine | 1971 | | H3N8 | | HA | | CY034934 | |  |
|  | A/equine/Sachiyama/1/1971/H3N8 | Japan | | Equine | 1971 | | H3N8 | | NP | | CY034937 | |  |
|  | A/equine/Sachiyama/1/1971/H3N8 | Japan | | Equine | 1971 | | H3N8 | | NA | | CY034936 | |  |
|  | A/equine/Sachiyama/1/1971/H3N8 | Japan | | Equine | 1971 | | H3N8 | | MP | | CY034935 | |  |
|  | A/equine/Sachiyama/1/1971/H3N8 | Japan | | Equine | 1971 | | H3N8 | | NS | | CY034938 | |  |
|  | A/equine/Tokyo/2/1971H3N8 | Japan | | Equine | 1971 | | H3N8 | | PB2 | | CY096922 | |  |
|  | A/equine/Tokyo/2/1971H3N8 | Japan | | Equine | 1971 | | H3N8 | | PB1 | | CY096921 | |  |
|  | A/equine/Tokyo/2/1971H3N8 | Japan | | Equine | 1971 | | H3N8 | | PA | | CY096920 | |  |
|  | A/equine/Tokyo/2/1971H3N8 | Japan | | Equine | 1971 | | H3N8 | | HA | | CY096915 | |  |
|  | A/equine/Tokyo/2/1971H3N8 | Japan | | Equine | 1971 | | H3N8 | | NP | | CY096918 | |  |
|  | A/equine/Tokyo/2/1971H3N8 | Japan | | Equine | 1971 | | H3N8 | | NA | | CY096917 | |  |
|  | A/equine/Tokyo/2/1971H3N8 | Japan | | Equine | 1971 | | H3N8 | | MP | | CY096916 | |  |
|  | A/equine/Tokyo/2/1971H3N8 | Japan | | Equine | 1971 | | H3N8 | | NS | | CY096919 | |  |
|  | A/equine/Sao Paulo/1/1969/H3N8 | Brazil | | Equine | 1969 | | H3N8 | | PB2 | | CY032404 | |  |
|  | A/equine/Sao Paulo/1/1969/H3N8 | Brazil | | Equine | 1969 | | H3N8 | | PB1 | | CY032403 | |  |
|  | A/equine/Sao Paulo/1/1969/H3N8 | Brazil | | Equine | 1969 | | H3N8 | | PA | | CY032402 | |  |
|  | A/equine/Sao Paulo/1/1969/H3N8 | Brazil | | Equine | 1969 | | H3N8 | | HA | | CY032397 | |  |
|  | A/equine/Sao Paulo/1/1969/H3N8 | Brazil | | Equine | 1969 | | H3N8 | | NP | | CY032400 | |  |
|  | A/equine/Sao Paulo/1/1969/H3N8 | Brazil | | Equine | 1969 | | H3N8 | | NA | | CY032399 | |  |
|  | A/equine/Sao Paulo/1/1969/H3N8 | Brazil | | Equine | 1969 | | H3N8 | | MP | | CY032398 | |  |
|  | A/equine/Sao Paulo/1/1969/H3N8 | Brazil | | Equine | 1969 | | H3N8 | | NS | | CY032401 | |  |
|  | A/equine/Lexington/1/1966/H7N7 | USA | | Equine | 1966 | | H7N7 | | PB2 | | CY039398 | |  |
|  | A/equine/Lexington/1/1966/H7N7 | USA | | Equine | 1966 | | H7N7 | | PB1 | | CY039397 | |  |
|  | A/equine/Lexington/1/1966/H7N7 | USA | | Equine | 1966 | | H7N7 | | PA | | CY039396 | |  |
|  | A/equine/Lexington/1/1966/H7N7 | USA | | Equine | 1966 | | H7N7 | | HA | | CY039391 | |  |
|  | A/equine/Lexington/1/1966/H7N7 | USA | | Equine | 1966 | | H7N7 | | NP | | CY039394 | |  |
|  | A/equine/Lexington/1/1966/H7N7 | USA | | Equine | 1966 | | H7N7 | | NA | | CY039393 | |  |
|  | A/equine/Lexington/1/1966/H7N7 | USA | | Equine | 1966 | | H7N7 | | MP | | CY039392 | |  |
|  | A/equine/Lexington/1/1966/H7N7 | USA | | Equine | 1966 | | H7N7 | | NS | | CY039395 | |  |
|  | A/equine/Detroit/3/1964/H7N7 | USA | | Equine | 1964 | | H7N7 | | PB2 | | KF435054 | |  |
|  | A/equine/Detroit/3/1964/H7N7 | USA | | Equine | 1964 | | H7N7 | | PB1 | | KF435055 | |  |
|  | A/equine/Detroit/3/1964/H7N7 | USA | | Equine | 1964 | | H7N7 | | PA | | KF435056 | |  |
|  | A/equine/Detroit/3/1964/H7N7 | USA | | Equine | 1964 | | H7N7 | | HA | | KF435057 | |  |
|  | A/equine/Detroit/3/1964/H7N7 | USA | | Equine | 1964 | | H7N7 | | NP | | KF435058 | |  |
|  | A/equine/Detroit/3/1964/H7N7 | USA | | Equine | 1964 | | H7N7 | | NA | | KF435059 | |  |
|  | A/equine/Detroit/3/1964/H7N7 | USA | | Equine | 1964 | | H7N7 | | MP | | KF435060 | |  |
|  | A/equine/Detroit/3/1964/H7N7 | USA | | Equine | 1964 | | H7N7 | | NS | | KF435061 | |  |
|  | A/equine/Miami/1/1963/H3N8 | USA | | Equine | 1963 | | H3N8 | | PB2 | | CY028843 | |  |
|  | A/equine/Miami/1/1963/H3N8 | USA | | Equine | 1963 | | H3N8 | | PB1 | | CY028842 | |  |
|  | A/equine/Miami/1/1963/H3N8 | USA | | Equine | 1963 | | H3N8 | | PA | | CY028841 | |  |
|  | A/equine/Miami/1/1963/H3N8 | USA | | Equine | 1963 | | H3N8 | | HA | | CY028836 | |  |
|  | A/equine/Miami/1/1963/H3N8 | USA | | Equine | 1963 | | H3N8 | | NP | | CY028839 | |  |
|  | A/equine/Miami/1/1963/H3N8 | USA | | Equine | 1963 | | H3N8 | | NA | | CY028838 | |  |
|  | A/equine/Miami/1/1963/H3N8 | USA | | Equine | 1963 | | H3N8 | | MP | | CY028837 | |  |
|  | A/equine/Miami/1/1963/H3N8 | USA | | Equine | 1963 | | H3N8 | | NS | | CY028840 | |  |
|  | A/equine/Sao Paulo/6/1963/H3N8 | Brazil | | Equine | 1963 | | H3N8 | | PB2 | | CY032300 | |  |
|  | A/equine/Sao Paulo/6/1963/H3N8 | Brazil | | Equine | 1963 | | H3N8 | | PB1 | | CY032299 | |  |
|  | A/equine/Sao Paulo/6/1963/H3N8 | Brazil | | Equine | 1963 | | H3N8 | | PA | | CY032298 | |  |
|  | A/equine/Sao Paulo/6/1963/H3N8 | Brazil | | Equine | 1963 | | H3N8 | | HA | | CY032293 | |  |
|  | A/equine/Sao Paulo/6/1963/H3N8 | Brazil | | Equine | 1963 | | H3N8 | | NP | | CY032296 | |  |
|  | A/equine/Sao Paulo/6/1963/H3N8 | Brazil | | Equine | 1963 | | H3N8 | | NA | | CY032295 | |  |
|  | A/equine/Sao Paulo/6/1963/H3N8 | Brazil | | Equine | 1963 | | H3N8 | | MP | | CY032294 | |  |
|  | A/equine/Sao Paulo/6/1963/H3N8 | Brazil | | Equine | 1963 | | H3N8 | | NS | | CY032297 | |  |
|  | A/equine/Uruguay/1/1963/H3N8 | Uruguay | | Equine | 1963 | | H3N8 | | PB2 | | CY032428 | |  |
|  | A/equine/Uruguay/1/1963/H3N8 | Uruguay | | Equine | 1963 | | H3N8 | | PB1 | | CY032427 | |  |
|  | A/equine/Uruguay/1/1963/H3N8 | Uruguay | | Equine | 1963 | | H3N8 | | PA | | CY032426 | |  |
|  | A/equine/Uruguay/1/1963/H3N8 | Uruguay | | Equine | 1963 | | H3N8 | | HA | | CY032421 | |  |
|  | A/equine/Uruguay/1/1963/H3N8 | Uruguay | | Equine | 1963 | | H3N8 | | NP | | CY032424 | |  |
|  | A/equine/Uruguay/1/1963/H3N8 | Uruguay | | Equine | 1963 | | H3N8 | | NA | | CY032423 | |  |
|  | A/equine/Uruguay/1/1963/H3N8 | Uruguay | | Equine | 1963 | | H3N8 | | MP | | CY032422 | |  |
|  | A/equine/Uruguay/1/1963/H3N8 | Uruguay | | Equine | 1963 | | H3N8 | | NS | | CY032425 | |  |
|  | A/equine/Prague/1/1956/H7N7 | Czech Republic | | Equine | 1956 | | H7N7 | | PB2 | | CY096914 | |  |
|  | A/equine/Prague/1/1956/H7N7 | Czech Republic | | Equine | 1956 | | H7N7 | | PB1 | | CY096913 | |  |
|  | A/equine/Prague/1/1956/H7N7 | Czech Republic | | Equine | 1956 | | H7N7 | | PA | | CY096912 | |  |
|  | A/equine/Prague/1/1956/H7N7 | Czech Republic | | Equine | 1956 | | H7N7 | | HA | | CY096907 | |  |
|  | A/equine/Prague/1/1956/H7N7 | Czech Republic | | Equine | 1956 | | H7N7 | | NP | | CY096910 | |  |
|  | A/equine/Prague/1/1956/H7N7 | Czech Republic | | Equine | 1956 | | H7N7 | | NA | | CY096909 | |  |
|  | A/equine/Prague/1/1956/H7N7 | Czech Republic | | Equine | 1956 | | H7N7 | | MP | | CY096908 | |  |
|  | A/equine/Prague/1/1956/H7N7 | Czech Republic | | Equine | 1956 | | H7N7 | | NS | | CY096911 | |  |
|  | A/equine/Prague/1956/H7N7 | Czech Republic | | Equine | 1956 | | H7N7 | | PB2 | | CY130141 | |  |
|  | A/equine/Prague/1956/H7N7 | Czech Republic | | Equine | 1956 | | H7N7 | | PB1 | | CY130140 | |  |
|  | A/equine/Prague/1956/H7N7 | Czech Republic | | Equine | 1956 | | H7N7 | | PA | | CY130139 | |  |
|  | A/equine/Prague/1956/H7N7 | Czech Republic | | Equine | 1956 | | H7N7 | | HA | | CY130134 | |  |
|  | A/equine/Prague/1956/H7N7 | Czech Republic | | Equine | 1956 | | H7N7 | | NP | | CY130137 | |  |
|  | A/equine/Prague/1956/H7N7 | Czech Republic | | Equine | 1956 | | H7N7 | | NA | | CY130136 | |  |
|  | A/equine/Prague/1956/H7N7 | Czech Republic | | Equine | 1956 | | H7N7 | | MP | | CY130135 | |  |
|  | A/equine/Prague/1956/H7N7 | Czech Republic | | Equine | 1956 | | H7N7 | | NS | | CY130138 | |  |
|  | A/equine/Prague/2/1956/H7N7 | Czech Republic | | Equine | 1956 | | H7N7 | | PB2 | | CY087823 | |  |
|  | A/equine/Prague/2/1956/H7N7 | Czech Republic | | Equine | 1956 | | H7N7 | | PB1 | | CY087822 | |  |
|  | A/equine/Prague/2/1956/H7N7 | Czech Republic | | Equine | 1956 | | H7N7 | | PA | | CY087821 | |  |
|  | A/equine/Prague/2/1956/H7N7 | Czech Republic | | Equine | 1956 | | H7N7 | | HA | | CY087816 | |  |
|  | A/equine/Prague/2/1956/H7N7 | Czech Republic | | Equine | 1956 | | H7N7 | | NP | | CY087819 | |  |
|  | A/equine/Prague/2/1956/H7N7 | Czech Republic | | Equine | 1956 | | H7N7 | | NA | | CY087818 | |  |
|  | A/equine/Prague/2/1956/H7N7 | Czech Republic | | Equine | 1956 | | H7N7 | | MP | | CY087817 | |  |
|  | A/equine/Prague/2/1956/H7N7 | Czech Republic | | Equine | 1956 | | H7N7 | | NS | | CY087820 | |  |
