# Supplementary material for: Revelation of Influencing Factors in Overall Codon Usage Bias of Equine Influenza Viruses
Source: PLoS One. 2016 Apr 27;11(4):e0154376. doi: 10.1371/journal.pone.0154376 (PMC4847779; doi:10.1371/journal.pone.0154376)
Supplement: S2 Table — (DOCX) [file pone.0154376.s006.docx]

**S2 Table: Nucleotide compositional analysis of EIV genomes.**

| **S. No.** | **EIV Strains** | **A(%)** | **C(%)** | **G(%)** | **U(%)** | **U3(%)** | **C3(%)** | **A3(%)** | **G3(%)** | **GC**  **(%)** | **AU**  **(%)** | **GC1**  **(%)** | **GC2**  **(%)** | **GC3**  **(%)** | **AU3**  **(%)** | **GC12**  **(%)** | **ENC** |
| --- | --- | --- | --- | --- | --- | --- | --- | --- | --- | --- | --- | --- | --- | --- | --- | --- | --- |
| 1 | A/equine/Heilongjiang/SS1/2013/H3N8 | 34.5 | 19.1 | 23.1 | 23.2 | 24.2 | 20.6 | 34.8 | 20.4 | 42.2 | 57.7 | 45.8 | 40.2 | 41 | 59 | 43.0 | 51.18 |
| 2 | A/equine/Xuzhou/01/2013/H3N8 | 34.6 | 19.2 | 23.1 | 23.1 | 23.8 | 20.8 | 34.9 | 20.6 | 42.3 | 57.7 | 45.7 | 40 | 41.4 | 58.7 | 42.9 | 51.16 |
| 3 | A/equine/Kyonggi/SA1/2011/H3N8 | 34.5 | 19.0 | 23.4 | 23.1 | 23.8 | 20.5 | 34.6 | 21 | 42.3 | 57.6 | 45.6 | 40.1 | 41.5 | 58.4 | 42.9 | 51.56 |
| 4 | A/equine/Heilongjiang/1/2010/H3N8 | 34.5 | 19.2 | 23.3 | 23.1 | 23.7 | 20.8 | 34.5 | 21 | 42.4 | 57.5 | 45.7 | 40 | 41.8 | 58.2 | 42.9 | 51.48 |
| 5 | A/equine/Gansu/7/2008/H3N8 | 34.5 | 19.2 | 23.3 | 23.1 | 23.8 | 20.6 | 34.6 | 20.9 | 42.4 | 57.5 | 45.9 | 40.1 | 41.5 | 58.4 | 43.0 | 51.45 |
| 6 | A/equine/Guangxi/1/2008/H3N8 | 34.5 | 19.2 | 23.2 | 23.1 | 23.8 | 20.7 | 34.6 | 20.9 | 42.4 | 57.6 | 45.7 | 40 | 41.6 | 58.4 | 42.9 | 51.45 |
| 7 | A/equine/Heilongjiang/10/2008/H3N8 | 34.5 | 19.2 | 23.3 | 23.0 | 23.8 | 20.6 | 34.7 | 20.9 | 42.4 | 57.5 | 45.9 | 40.2 | 41.5 | 58.5 | 43.1 | 51.46 |
| 8 | A/equine/Inner_Mongolia/8/2008/H3N8 | 34.5 | 19.2 | 23.3 | 23.0 | 23.7 | 20.7 | 34.6 | 21 | 42.5 | 57.5 | 45.9 | 40.1 | 41.7 | 58.3 | 43.0 | 51.54 |
| 9 | A/equine/Liaoning/9/2008/H3N8 | 34.6 | 19.2 | 23.2 | 23.1 | 23.9 | 20.5 | 34.8 | 20.8 | 42.3 | 57.6 | 45.9 | 40.1 | 41.3 | 58.7 | 43.0 | 51.51 |
| 10 | A/donkey/Xinjiang/5/2007/H3N8 | 34.5 | 19.2 | 23.3 | 23.1 | 23.8 | 20.6 | 34.6 | 20.9 | 42.4 | 57.6 | 45.8 | 40 | 41.5 | 58.4 | 42.9 | 51.5 |
| 11 | A/equine/Huabei/1/2007/H3N8 | 34.4 | 19.2 | 23.3 | 23.1 | 24 | 20.7 | 34.6 | 20.7 | 42.4 | 57.5 | 45.8 | 40.3 | 41.4 | 58.6 | 43.1 | 51.4 |
| 12 | A/equine/Richmond/1/2007/H3N8 | 34.4 | 19.0 | 23.3 | 23.3 | 24.4 | 20.4 | 34.7 | 20.6 | 42.2 | 57.7 | 45.5 | 40.6 | 41 | 59.1 | 43.1 | 51.26 |
| 13 | A/equine/Tottori/1/2007/H3N8 | 34.3 | 19.1 | 23.4 | 23.2 | 24.1 | 20.6 | 34.5 | 20.8 | 42.5 | 57.5 | 46 | 40.3 | 41.4 | 58.6 | 43.2 | 51.57 |
| 14 | A/equine/Xinjiang/1/2007/H3N8 | 34.5 | 19.2 | 23.3 | 23.0 | 23.8 | 20.6 | 34.6 | 21 | 42.5 | 57.5 | 46 | 40.2 | 41.6 | 58.4 | 43.1 | 51.53 |
| 15 | A/equine/Xinjiang/2/2007/H3N8 | 34.5 | 19.2 | 23.3 | 23.0 | 23.8 | 20.7 | 34.6 | 21 | 42.5 | 57.5 | 46 | 40.1 | 41.7 | 58.4 | 43.1 | 51.51 |
| 16 | A/equine/Xinjiang/3/2007/H3N8 | 34.4 | 19.2 | 23.3 | 23.1 | 23.8 | 20.7 | 34.5 | 21 | 42.5 | 57.5 | 45.9 | 40.1 | 41.7 | 58.3 | 43.0 | 51.63 |
| 17 | A/equine/Xinjiang/4/2007/H3N8 | 34.4 | 19.2 | 23.3 | 23.0 | 23.8 | 20.7 | 34.6 | 20.9 | 42.5 | 57.5 | 46.1 | 40.2 | 41.6 | 58.4 | 43.2 | 51.43 |
| 18 | A/equine/Newmarket/5/2003/H3N8 | 34.3 | 19.2 | 23.4 | 23.1 | 23.9 | 20.6 | 34.5 | 21.1 | 42.5 | 57.4 | 46.1 | 40.1 | 41.7 | 58.4 | 43.1 | 51.5 |
| 19 | A/equine/Wisconsin/1/2003/H3N8 | 34.3 | 19.1 | 23.4 | 23.2 | 24.2 | 20.5 | 34.4 | 20.9 | 42.5 | 57.5 | 46.2 | 40.3 | 41.4 | 58.6 | 43.3 | 51.37 |
| 20 | A/equine/California/8560/2002/H3N8 | 34.3 | 19.2 | 23.4 | 23.1 | 23.8 | 20.6 | 34.4 | 21.1 | 42.6 | 57.4 | 46.2 | 40.1 | 41.7 | 58.2 | 43.2 | 51.54 |
| 21 | A/equine/Kentucky/5/2002/H3N8 | 34.3 | 19.3 | 23.5 | 22.9 | 23.9 | 20.9 | 34.6 | 20.6 | 42.7 | 57.2 | 46.1 | 40.7 | 41.5 | 58.5 | 43.4 | 51.38 |
| 22 | A/equine/California/4537/1997/H3N8 | 34.2 | 19.2 | 23.5 | 23.0 | 23.8 | 20.6 | 34.1 | 21.4 | 42.7 | 57.3 | 46.3 | 40.1 | 42 | 57.9 | 43.2 | 51.64 |
| 23 | A/equine/Kentucky/8/1994/H3N8 | 34.2 | 19.3 | 23.5 | 23.0 | 23.6 | 20.9 | 34.1 | 21.4 | 42.8 | 57.2 | 46.1 | 40.2 | 42.3 | 57.7 | 43.2 | 51.61 |
| 24 | A/equine/Qinghai/1/1994/H3N8 | 34.0 | 19.2 | 23.7 | 23.1 | 23.9 | 20.6 | 33.6 | 21.9 | 42.8 | 57.1 | 46.2 | 40.1 | 42.5 | 57.5 | 43.2 | 52.33 |
| 25 | A/equine/Switzerland/173/1993/H3N8 | 34.0 | 19.3 | 23.8 | 23.0 | 23.7 | 20.8 | 33.7 | 21.9 | 43.0 | 57.0 | 46.3 | 40.3 | 42.7 | 57.4 | 43.3 | 52.18 |
| 26 | A/equine/Austria/421/1992/H3N8 | 33.9 | 19.3 | 23.8 | 23.0 | 23.6 | 20.9 | 33.5 | 22 | 43.1 | 56.9 | 46.3 | 40.3 | 42.9 | 57.1 | 43.3 | 52.22 |
| 27 | A/equine/Italy/1199/1992/H3N8 | 34.0 | 19.3 | 23.8 | 23.0 | 23.6 | 20.8 | 33.7 | 21.9 | 43.0 | 57.0 | 46.3 | 40.2 | 42.7 | 57.3 | 43.3 | 52.14 |
| 28 | A/equine/Kentucky/1/1992/H3N8 | 34.1 | 19.3 | 23.7 | 22.9 | 23.5 | 21 | 33.8 | 21.7 | 42.9 | 57.0 | 46.1 | 40.3 | 42.7 | 57.3 | 43.2 | 51.86 |
| 29 | A/equine/Alaska/29759/1991/H3N8 | 34.1 | 19.4 | 23.7 | 22.9 | 23.4 | 21.1 | 33.7 | 21.8 | 43.0 | 57.0 | 46.2 | 40.2 | 42.9 | 57.1 | 43.2 | 51.99 |
| 30 | A/equine/Italy/1062/1991/H3N8 | 34.0 | 19.3 | 23.8 | 22.9 | 23.6 | 20.9 | 33.5 | 22 | 43.1 | 56.9 | 46.4 | 40.2 | 42.9 | 57.1 | 43.3 | 52.32 |
| 31 | A/equine/Italy/824/1991/H3N8 | 34.0 | 19.3 | 23.8 | 23.0 | 23.6 | 20.8 | 33.5 | 22 | 43.0 | 56.9 | 46.3 | 40.2 | 42.8 | 57.1 | 43.3 | 52.3 |
| 32 | A/equine/Kentucky/1/1991/H3N8 | 34.1 | 19.4 | 23.7 | 22.9 | 23.4 | 21.1 | 33.8 | 21.8 | 43.0 | 57.0 | 46.2 | 40.2 | 42.9 | 57.2 | 43.2 | 51.72 |
| 33 | A/equine/Rome/5/1991/H3N8 | 33.9 | 19.3 | 23.8 | 23.0 | 23.7 | 20.8 | 33.4 | 22 | 43.0 | 56.9 | 46.4 | 40.3 | 42.8 | 57.1 | 43.4 | 52.34 |
| 34 | A/equine/Texas/39655/1991/H3N8 | 34.1 | 19.4 | 23.7 | 22.9 | 23.3 | 21.1 | 33.7 | 21.8 | 43.0 | 56.9 | 46.3 | 40.2 | 42.9 | 57 | 43.3 | 51.94 |
| 35 | A/equine/Kentucky/1277/1990/H3N8 | 33.9 | 19.4 | 23.8 | 22.9 | 23.3 | 21.2 | 33.5 | 22 | 43.2 | 56.8 | 46.2 | 40.3 | 43.2 | 56.8 | 43.3 | 52.11 |
| 36 | A/equine/Berlin/1/1989/H3N8 | 33.9 | 19.3 | 23.8 | 22.9 | 23.5 | 20.9 | 33.5 | 22.1 | 43.1 | 56.8 | 46.3 | 40.4 | 43 | 57 | 43.4 | 52.3 |
| 37 | A/equine/Jilin/1/1989/H3N8 | 32.5 | 19.5 | 25.0 | 23.0 | 24.4 | 21 | 29.8 | 24.8 | 44.5 | 55.5 | 47.5 | 40.4 | 45.8 | 54.2 | 44.0 | 53.22 |
| 38 | A/equine/Rook/93753/1989/H3N8 | 33.9 | 19.3 | 23.9 | 22.9 | 23.6 | 20.9 | 33.3 | 22.2 | 43.1 | 56.8 | 46.3 | 40.4 | 43.1 | 56.9 | 43.4 | 52.35 |
| 39 | A/equine/Sussex/1/1989/H3N8 | 33.9 | 19.3 | 23.8 | 23.0 | 23.6 | 20.9 | 33.3 | 22.2 | 43.1 | 56.8 | 46.4 | 40.4 | 43.1 | 56.9 | 43.4 | 52.43 |
| 40 | A/equine/Kentucky/692/1988/H3N8 | 33.9 | 19.4 | 23.9 | 22.9 | 23.4 | 21.1 | 33.3 | 22.2 | 43.2 | 56.7 | 46.4 | 40.4 | 43.3 | 56.7 | 43.4 | 52.22 |
| 41 | A/equine/Kentucky/694/1988/H3N8 | 33.9 | 19.4 | 23.9 | 22.9 | 23.4 | 21.1 | 33.3 | 22.2 | 43.2 | 56.7 | 46.3 | 40.4 | 43.3 | 56.7 | 43.4 | 52.19 |
| 42 | A/equine/Kentucky/698/1988/H3N8 | 33.9 | 19.4 | 23.8 | 22.9 | 23.4 | 21.1 | 33.3 | 22.2 | 43.2 | 56.8 | 46.3 | 40.4 | 43.3 | 56.7 | 43.4 | 52.23 |
| 43 | A/equine/Kentucky/1/1987/H3N8 | 33.8 | 19.5 | 24.0 | 22.8 | 23.3 | 21.2 | 33.1 | 22.4 | 43.4 | 56.6 | 46.4 | 40.4 | 43.6 | 56.4 | 43.4 | 52.4 |
| 44 | A/equine/Kentucky/2/1987/H3N8 | 33.8 | 19.5 | 24.0 | 22.8 | 23.3 | 21.2 | 33.1 | 22.4 | 43.3 | 56.6 | 46.4 | 40.4 | 43.6 | 56.4 | 43.4 | 52.4 |
| 45 | A/equine/Johannesburg/1/1986/H3N8 | 33.8 | 19.5 | 24.0 | 22.8 | 23.2 | 21.3 | 33 | 22.5 | 43.4 | 56.5 | 46.3 | 40.4 | 43.8 | 56.2 | 43.4 | 52.47 |
| 46 | A/equine/Kentucky/1/1986/H3N8 | 33.7 | 19.6 | 24.0 | 22.7 | 23.2 | 21.4 | 33 | 22.5 | 43.5 | 56.5 | 46.4 | 40.4 | 43.9 | 56.2 | 43.4 | 52.45 |
| 47 | A/equine/Kentucky/2/1986/H3N8 | 33.8 | 19.6 | 24.0 | 22.7 | 23.1 | 21.3 | 33 | 22.5 | 43.5 | 56.5 | 46.4 | 40.4 | 43.8 | 56.1 | 43.4 | 52.46 |
| 48 | A/equine/Kentucky/3/1986/H3N8 | 33.8 | 19.5 | 24.0 | 22.8 | 23.2 | 21.3 | 33 | 22.5 | 43.4 | 56.5 | 46.4 | 40.4 | 43.8 | 56.2 | 43.4 | 52.41 |
| 49 | A/equine/Tennessee/5/1986/H3N8 | 33.8 | 19.5 | 23.9 | 22.8 | 23.3 | 21.2 | 33.3 | 22.2 | 43.3 | 56.7 | 46.3 | 40.4 | 43.4 | 56.6 | 43.4 | 52.29 |
| 50 | A/equine/Cordoba/18/1985/H3N8 | 33.8 | 19.5 | 23.9 | 22.8 | 23.4 | 21.2 | 33.1 | 22.4 | 43.3 | 56.7 | 46.3 | 40.3 | 43.6 | 56.5 | 43.3 | 52.44 |
| 51 | A/equine/Santa_Fe/1/1985/H3N8 | 33.8 | 19.5 | 23.9 | 22.8 | 23.3 | 21.2 | 33.1 | 22.3 | 43.3 | 56.6 | 46.3 | 40.4 | 43.5 | 56.4 | 43.4 | 52.43 |
| 52 | A/equine/New_York/VR-297/1983/H3N8 | 33.5 | 19.7 | 24.2 | 22.6 | 22.9 | 21.7 | 32.3 | 23.1 | 43.9 | 56.1 | 46.8 | 40.4 | 44.8 | 55.2 | 43.6 | 53.01 |
| 53 | A/equine/California/103/1982/H3N8 | 33.7 | 19.6 | 24.0 | 22.7 | 23 | 21.5 | 32.9 | 22.6 | 43.6 | 56.4 | 46.6 | 40.4 | 44.1 | 55.9 | 43.5 | 52.49 |
| 54 | A/equine/California/83/1982/H3N8 | 33.7 | 19.6 | 24.0 | 22.7 | 23 | 21.5 | 32.9 | 22.6 | 43.6 | 56.4 | 46.6 | 40.4 | 44.1 | 55.9 | 43.5 | 52.5 |
| 55 | A/equine/Georgia/1/1981/H3N8 | 33.6 | 19.6 | 24.1 | 22.7 | 23 | 21.5 | 32.7 | 22.7 | 43.7 | 56.3 | 46.7 | 40.4 | 44.2 | 55.7 | 43.6 | 52.65 |
| 56 | A/equine/Georgia/10/1981/H3N8 | 33.6 | 19.6 | 24.1 | 22.7 | 23 | 21.5 | 32.7 | 22.8 | 43.7 | 56.3 | 46.7 | 40.4 | 44.3 | 55.7 | 43.6 | 52.67 |
| 57 | A/equine/Georgia/13/1981/H3N8 | 33.6 | 19.6 | 24.1 | 22.7 | 23 | 21.6 | 32.7 | 22.8 | 43.7 | 56.3 | 46.6 | 40.4 | 44.4 | 55.7 | 43.5 | 52.71 |
| 58 | A/equine/Georgia/3/1981/H3N8 | 33.6 | 19.6 | 24.1 | 22.7 | 23 | 21.5 | 32.7 | 22.8 | 43.7 | 56.3 | 46.7 | 40.4 | 44.3 | 55.7 | 43.6 | 52.68 |
| 59 | A/equine/Georgia/9/1981/H3N8 | 33.6 | 19.6 | 24.1 | 22.7 | 23 | 21.6 | 32.7 | 22.7 | 43.7 | 56.3 | 46.7 | 40.4 | 44.3 | 55.7 | 43.6 | 52.67 |
| 60 | A/equine/Kentucky/1/1981/H3N8 | 33.6 | 19.7 | 24.1 | 22.6 | 22.9 | 21.6 | 32.7 | 22.8 | 43.7 | 56.2 | 46.7 | 40.4 | 44.4 | 55.6 | 43.6 | 52.8 |
| 61 | A/equine/Kentucky/2/1981/H3N8 | 33.7 | 19.7 | 24.1 | 22.6 | 22.9 | 21.6 | 32.8 | 22.7 | 43.7 | 56.3 | 46.6 | 40.4 | 44.3 | 55.7 | 43.5 | 52.78 |
| 62 | A/equine/Kentucky/3/1981/H3N8 | 33.6 | 19.7 | 24.1 | 22.6 | 22.9 | 21.6 | 32.7 | 22.8 | 43.7 | 56.2 | 46.7 | 40.4 | 44.4 | 55.6 | 43.6 | 52.81 |
| 63 | A/equine/Kentucky/Rosie100/1981/H3N8 | 33.7 | 19.6 | 24.0 | 22.7 | 23.1 | 21.4 | 32.8 | 22.7 | 43.5 | 56.4 | 46.4 | 40.4 | 44.1 | 55.9 | 43.4 | 52.61 |
| 64 | A/equine/Kentucky/magnificent_genius1/1981/H3N8 | 33.7 | 19.6 | 24.1 | 22.7 | 23.1 | 21.4 | 32.8 | 22.7 | 43.6 | 56.4 | 46.5 | 40.4 | 44.1 | 55.9 | 43.5 | 52.6 |
| 65 | A/equine/California/1/1980/H3N8 | 33.6 | 19.7 | 24.2 | 22.6 | 22.9 | 21.6 | 32.5 | 23 | 43.8 | 56.2 | 46.7 | 40.4 | 44.6 | 55.4 | 43.6 | 52.88 |
| 66 | A/equine/Kentucky/2/1980/H3N8 | 33.6 | 19.7 | 24.1 | 22.6 | 22.9 | 21.6 | 32.6 | 22.9 | 43.7 | 56.2 | 46.7 | 40.4 | 44.5 | 55.5 | 43.6 | 52.73 |
| 67 | A/equine/Kentucky/4/1980/H3N8 | 33.6 | 19.6 | 24.2 | 22.7 | 23 | 21.6 | 32.4 | 23 | 43.8 | 56.2 | 46.6 | 40.3 | 44.6 | 55.4 | 43.5 | 52.85 |
| 68 | A/equine/Romania/1/1980/H3N8 | 33.6 | 19.6 | 24.2 | 22.7 | 23.2 | 21.3 | 32.5 | 22.9 | 43.7 | 56.3 | 46.7 | 40.4 | 44.2 | 55.7 | 43.6 | 52.6 |
| 69 | A/equine/Fontainebleau/1/1979/H3N8 | 33.6 | 19.7 | 24.2 | 22.6 | 23 | 21.6 | 32.5 | 22.9 | 43.8 | 56.2 | 46.8 | 40.4 | 44.5 | 55.5 | 43.6 | 52.74 |
| 70 | A/equine/New_Market/1/1979/H3N8 | 33.5 | 19.7 | 24.2 | 22.6 | 23 | 21.6 | 32.4 | 23 | 43.8 | 56.2 | 46.7 | 40.4 | 44.6 | 55.4 | 43.6 | 52.78 |
| 71 | A/equine/New_Market/nasalwash1/1979/H3N8 | 33.6 | 19.7 | 24.2 | 22.6 | 23 | 21.6 | 32.5 | 22.9 | 43.8 | 56.2 | 46.7 | 40.4 | 44.5 | 55.5 | 43.6 | 52.74 |
| 72 | A/equine/Switzerland/1118/1979/H3N8 | 33.5 | 19.7 | 24.2 | 22.7 | 23.1 | 21.5 | 32.4 | 23 | 43.8 | 56.2 | 46.8 | 40.4 | 44.5 | 55.5 | 43.6 | 52.7 |
| 73 | A/equine/Kascakew/1/1978/H3N8 | 33.6 | 19.7 | 24.2 | 22.6 | 23 | 21.6 | 32.5 | 23 | 43.8 | 56.2 | 46.7 | 40.4 | 44.6 | 55.5 | 43.6 | 52.73 |
| 74 | A/equine/Kentucky/1/1978/H3N8 | 33.5 | 19.7 | 24.2 | 22.6 | 23.1 | 21.6 | 32.3 | 23.1 | 43.9 | 56.1 | 46.8 | 40.4 | 44.7 | 55.4 | 43.6 | 52.83 |
| 75 | A/equine/Argentina/1/1977/H7N7 | 34.3 | 19.5 | 23.4 | 22.9 | 24.3 | 21.1 | 33.3 | 21.3 | 42.8 | 57.2 | 46.3 | 40 | 42.4 | 57.6 | 43.2 | 52.26 |
| 76 | A/equine/Kentucky/bitter_boredom5/1976/H3N8 | 33.7 | 19.6 | 24.1 | 22.7 | 23.1 | 21.4 | 32.8 | 22.7 | 43.6 | 56.4 | 46.4 | 40.4 | 44.1 | 55.9 | 43.4 | 52.61 |
| 77 | A/equine/Kentucky/pass_the_pepper1/1976/H3N8 | 33.7 | 19.5 | 24.1 | 22.7 | 23.1 | 21.4 | 32.8 | 22.7 | 43.6 | 56.4 | 46.5 | 40.5 | 44.1 | 55.9 | 43.5 | 52.61 |
| 78 | A/equine/Sao_Paulo/4/1976/H7N7 | 34.3 | 19.5 | 23.3 | 22.9 | 24.3 | 21.2 | 33.3 | 21.3 | 42.8 | 57.2 | 46.3 | 40 | 42.5 | 57.6 | 43.2 | 52.24 |
| 79 | A/equine/Uruguay/1063/1976/H7N7 | 34.3 | 19.5 | 23.3 | 22.8 | 24.2 | 21.2 | 33.4 | 21.2 | 42.8 | 57.2 | 46.2 | 40.1 | 42.4 | 57.6 | 43.2 | 52.23 |
| 80 | A/equine/Kentucky/1a/1975/H7N7 | 34.3 | 19.5 | 23.4 | 22.9 | 24.3 | 21.1 | 33.2 | 21.4 | 42.8 | 57.2 | 46.3 | 40 | 42.5 | 57.5 | 43.2 | 52.41 |
| 81 | A/equine/New_York/1/1975/H3N8 | 33.3 | 19.8 | 24.4 | 22.5 | 22.8 | 21.8 | 32 | 23.4 | 44.1 | 55.9 | 46.9 | 40.5 | 45.2 | 54.8 | 43.7 | 53.02 |
| 82 | A/equine/Sachiyama/1/1971/H3N8 | 33.4 | 19.7 | 24.2 | 22.7 | 23.2 | 21.7 | 32 | 23.1 | 43.9 | 56.0 | 47.2 | 40 | 44.8 | 55.2 | 43.6 | 52.96 |
| 83 | A/equine/Tokyo/2/1971/H3N8 | 33.3 | 19.7 | 24.3 | 22.7 | 23.2 | 21.7 | 32 | 23.1 | 44.0 | 56.0 | 47.3 | 40 | 44.8 | 55.2 | 43.7 | 53.17 |
| 84 | A/equine/Sao_Paulo/1/1969/H3N8 | 33.1 | 19.9 | 24.5 | 22.5 | 23.1 | 21.8 | 31.7 | 23.5 | 44.3 | 55.6 | 47.4 | 40.6 | 45.3 | 54.8 | 44.0 | 53.17 |
| 85 | A/equine/Lexington/1/1966/H7N7 | 36.9 | 18.2 | 21.1 | 23.8 | 26.2 | 18.1 | 39.5 | 16.1 | 39.2 | 60.7 | 44.6 | 39.2 | 34.2 | 65.7 | 41.9 | 47.92 |
| 86 | A/equine/Detroit/3/1964/H7N7 | 34.5 | 19.3 | 23.1 | 23.1 | 24.6 | 20.7 | 33.7 | 21 | 42.4 | 57.6 | 45.9 | 39.9 | 41.7 | 58.3 | 42.9 | 52.19 |
| 87 | A/equine/Miami/1/1963/H3N8 | 32.8 | 20.0 | 24.8 | 22.4 | 22.7 | 22.1 | 30.9 | 24.3 | 44.8 | 55.2 | 47.5 | 40.6 | 46.4 | 53.6 | 44.1 | 53.72 |
| 88 | A/equine/Sao_Paulo/6/1963/H3N8 | 33.2 | 19.9 | 24.4 | 22.5 | 23 | 21.8 | 31.7 | 23.5 | 44.3 | 55.7 | 47.3 | 40.5 | 45.3 | 54.7 | 43.9 | 53.15 |
| 89 | A/equine/Uruguay/1/1963/H3N8 | 32.8 | 20.0 | 24.8 | 22.4 | 22.7 | 22.1 | 30.9 | 24.3 | 44.7 | 55.2 | 47.4 | 40.6 | 46.4 | 53.6 | 44.0 | 53.65 |
| 90 | A/equine/Prague/1/1956/H7N7 | 36.7 | 18.3 | 21.2 | 23.8 | 26.2 | 18.3 | 39.2 | 16.2 | 39.4 | 60.5 | 44.7 | 39.4 | 34.5 | 65.4 | 42.1 | 47.85 |
| 91 | A/equine/Prague/1956/H7N7 | 36.8 | 18.3 | 21.2 | 23.8 | 26.2 | 18.3 | 39.2 | 16.2 | 39.4 | 60.5 | 44.7 | 39.3 | 34.5 | 65.4 | 42.0 | 47.7 |
| 92 | A/equine/Prague/2/1956/H7N7 | 36.8 | 18.3 | 21.2 | 23.8 | 26.3 | 18.3 | 39.3 | 16.2 | 39.4 | 60.5 | 44.7 | 39.4 | 34.5 | 65.6 | 42.1 | 47.81 |
|  | **Mean** | **34.0** | **19.4** | **23.7** | **22.9** | **23.6** | **21.0** | **33.5** | **21.9** | **43.1** | **56.9** | **46.3** | **40.3** | **42.9** | **57.1** | **43.3** | **52.1** |
|  | **SD** | **0.73** | **0.32** | **0.68** | **0.27** | **0.72** | **0.72** | **1.58** | **1.52** | **0.99** | **0.98** | **0.54** | **0.26** | **2.22** | **2.21** | **0.38** | **1.07** |
