# Supplementary material for: Revelation of Influencing Factors in Overall Codon Usage Bias of Equine Influenza Viruses
Source: PLoS One. 2016 Apr 27;11(4):e0154376. doi: 10.1371/journal.pone.0154376 (PMC4847779; doi:10.1371/journal.pone.0154376)
Supplement: S3 Table — (DOCX) [file pone.0154376.s007.docx]

**S3 Table: Comparative analysis of ENc values of H3N8 and H7N7 subtypes of EIVs.**

| **Genes** | **H3N8 (Mean±SD)** | **H7N7 (Mean±SD)** |
| --- | --- | --- |
| **HA** | 49.14±1.63 | 46.59±0.77 |
| **NA** | 50.30±1.24 | 44.29±0.53 |
| **MP** | 53.53±0.64 | 58.80±0.58 |
| **NP** | 52.43±0.60 | 49.42±2.95 |
| **NS** | 47.38±1.32 | 49.44±0.62 |
| **PA** | 52.27±0.55 | 50.34±3.50 |
| **PB1** | 53.41±0.68 | 51.90±3.04 |
| **PB2** | 49.89±0.67 | 48.38±1.56 |
