# Supplementary material for: Revelation of Influencing Factors in Overall Codon Usage Bias of Equine Influenza Viruses
Source: PLoS One. 2016 Apr 27;11(4):e0154376. doi: 10.1371/journal.pone.0154376 (PMC4847779; doi:10.1371/journal.pone.0154376)
Supplement: S4 Table — (DOCX) [file pone.0154376.s008.docx]

**S4 Table: Codon Adaptation Index (CAI) (both segment-wise and subtype-wise) of EIVs with respect to their potential hosts.**

| **Species** | **Genotypes** | **Different gene segments of EIVs** | | | | | | | |
| --- | --- | --- | --- | --- | --- | --- | --- | --- | --- |
|  |  | **HA** | **MP** | **NA** | **NP** | **NS** | **PA** | **PB1** | **PB2** |
| **Equine** | H3N8 | 0.654±0.004 | 0.681±0.005 | 0.632±0.003 | 0.679±0.005 | 0.637±0.003 | 0.675±0.004 | 0.677±0.006 | 0.647±0.009 |
|  | H7N7 | 0.633±0.005 | 0.640±0.008 | 0.623±0.003 | 0.670±0.01 | 0.629±0.01 | 0.663±0.02 | 0.670±0.01 | 0.633±0.02 |
| **Human** | H3N8 | 0.737±0.003 | 0.742±0.004 | 0.715±0.003 | 0.746±0.003 | 0.709±0.003 | 0.752±0.003 | 0.749±0.004 | 0.721±0.007 |
|  | H7N7 | 0.717±0.005 | 0.709±0.008 | 0.712±0.003 | 0.741±0.01 | 0.705±0.007 | 0.744±0.01 | 0.746±0.008 | 0.712±0.02 |
| **Pig** | H3N8 | 0.618±0.004 | 0.645±0.005 | 0.592±0.003 | 0.640±0.005 | 0.597±0.003 | 0.639±0.005 | 0.640±0.006 | 0.611±0.009 |
|  | H7N7 | 0.590±0.006 | 0.600±0.008 | 0.582±0.003 | 0.630±0.02 | 0.589±0.01 | 0.627±0.02 | 0.632±0.01 | 0.595±0.02 |
| **Macaque** | H3N8 | 0.819±0.004 | 0.815±0.004 | 0.802±0.005 | 0.823±0.003 | 0.791±0.002 | 0.830±0.002 | 0.823±0.003 | 0.799±0.006 |
|  | H7N7 | 0.811±0.005 | 0.791±0.006 | 0.805±0.002 | 0.822±0.001 | 0.789±0.004 | 0.827±0.008 | 0.824±0.001 | 0.796±0.01 |
| **Red Jungle Fowl** | H3N8 | 0.763±0.003 | 0.772±0.005 | 0.742±0.003 | 0.780±0.004 | 0.731±0.003 | 0.776±0.003 | 0.776±0.005 | 0.753±0.007 |
|  | H7N7 | 0.740±0.005 | 0.737±0.006 | 0.731±0.003 | 0.774±0.01 | 0.729±0.006 | 0.768±0.01 | 0.774±0.007 | 0.742±0.02 |
| **Duck** | H3N8 | 0.690±0.003 | 0.711±0.006 | 0.664±0.003 | 0.716±0.005 | 0.665±0.003 | 0.708±0.004 | 0.708±0.006 | 0.686±0.008 |
|  | H7N7 | 0.668±0.006 | 0.673±0.006 | 0.651±0.003 | 0.708±0.01 | 0.660±0.009 | 0.698±0.02 | 0.704±0.01 | 0.672±0.02 |
| **Goose** | H3N8 | 0.693±0.005 | 0.703±0.004 | 0.668±0.004 | 0.714±0.004 | 0.687±0.004 | 0.721±0.003 | 0.703±0.002 | 0.692±0.006 |
|  | H7N7 | 0.703±0.004 | 0.676±0.003 | 0.677±0.006 | 0.711±0.001 | 0.676±0.009 | 0.715±0.01 | 0.705±0.001 | 0.685±0.02 |
| **Dog** | H3N8 | 0.669±0.002 | 0.665±0.006 | 0.641±0.003 | 0.678±0.001 | 0.641±0.002 | 0.677±0.0005 | 0.672±0.0009 | 0.639±0.0005 |
