# Supplementary material for: Revelation of Influencing Factors in Overall Codon Usage Bias of Equine Influenza Viruses
Source: PLoS One. 2016 Apr 27;11(4):e0154376. doi: 10.1371/journal.pone.0154376 (PMC4847779; doi:10.1371/journal.pone.0154376)
Supplement: S5 Table — (DOCX) [file pone.0154376.s009.docx]

**S5 Table: Statistical analysis (ANOVA) of CAI values among different host species**.

| **Host species** | **Goose** | **Duck** | **Equine** | **Red Jungle Fowl** | **Human** | **Macaque** |
| --- | --- | --- | --- | --- | --- | --- |
| **Goose** |  |  |  |  |  |  |
| **Duck** | ns |  |  |  |  |  |
| **Equines** | ns | ns |  |  |  |  |
| **Red Jungle Fowl** | ns | ns | ** |  |  |  |
| **Human** | ns | ns | ns | ns |  |  |
| **Macaque** | * | ** | **** | ns | ns |  |
| **Pig** | ns | ns | ns | **** | ** | **** |

Note: ns- non-significant, *p>0.1, **p>0.01, ****p>0.0001
