# Supplementary material for: Revelation of Influencing Factors in Overall Codon Usage Bias of Equine Influenza Viruses
Source: PLoS One. 2016 Apr 27;11(4):e0154376. doi: 10.1371/journal.pone.0154376 (PMC4847779; doi:10.1371/journal.pone.0154376)
Supplement: S6 Table — (DOCX) [file pone.0154376.s010.docx]

**S6 Table: Comparative nucleotide compositional analysis of H3N8 viruses originated from horse and dog.**

| **Year of Isolation** | **Strains** | **A (%)** | **C(%)** | **G(%)** | **U(%)** | **GC(%)** | **ENC** |
| --- | --- | --- | --- | --- | --- | --- | --- |
| 2008 | A/equine/Guangxi/1/2008/H3N8 | 34.5 | 19.2 | 23.2 | 23.1 | 42.40 | 51.45 |
| 2008 | A/equine/Heilongjiang/10/2008/H3N8 | 34.5 | 19.2 | 23.3 | 23.0 | 42.48 | 51.46 |
| 2008 | A/equine/Inner_Mongolia/8/2008/H3N8 | 34.5 | 19.2 | 23.3 | 23.0 | 42.51 | 51.54 |
| 2008 | A/equine/Liaoning/9/2008/H3N8 | 34.6 | 19.2 | 23.2 | 23.1 | 42.39 | 51.51 |
| 2009 | A/canine/PA/111788/2009/H3N8 | 34.6 | 19.2 | 23.0 | 23.1 | 42.20 | 51.52 |
| 2010 | A/equine/Heilongjiang/1/2010/H3N8 | 34.5 | 19.2 | 23.3 | 23.1 | 42.47 | 51.48 |
| 2010 | A/canine/PA/33225.4/2010/H3N8 | 34.6 | 19.2 | 22.3 | 23.1 | 42.16 | 51.56 |
| 2011 | A/equine/Kyonggi/SA1/2011/H3N8 | 34.5 | 19.0 | 23.4 | 23.1 | 42.36 | 51.56 |
| 2011 | A/canine/NY/120106.2/2011/H3N8 | 34.8 | 19.2 | 22.9 | 23.1 | 42.09 | 51.43 |
| 2013 | A/equine/Heilongjiang/SS1/2013/H3N8 | 34.5 | 19.1 | 23.1 | 23.2 | 42.27 | 51.18 |
| 2013 | A/equine/Xuzhou/01/2013/H3N8 | 34.6 | 19.2 | 23.1 | 23.1 | 42.32 | 51.16 |
| 2013 | A/canine/VT/11039/2013/H3N8 | 34.9 | 19.2 | 22.8 | 23.1 | 41.98 | 51.26 |
