# Supplementary material for: Revelation of Influencing Factors in Overall Codon Usage Bias of Equine Influenza Viruses
Source: PLoS One. 2016 Apr 27;11(4):e0154376. doi: 10.1371/journal.pone.0154376 (PMC4847779; doi:10.1371/journal.pone.0154376)
Supplement: S7 Table — The sudden changes in GC contents in respective gene segments due to reassortment are highlighted. (DOCX) [file pone.0154376.s011.docx]

**S7 Table: Trends in GC compositions in all gene segments of H7N7 viruses over the period of time (1956-1977).** The sudden changes in GC contents in respective gene segments due to reassortment are highlighted.

| **Year of isolation** | **HA** | **MP** | **NA** | **NP** | **NS** | **PA** | **PB1** | **PB2** |
| --- | --- | --- | --- | --- | --- | --- | --- | --- |
| 1956 | 37.9 | 45.2 | 38.6 | 42 | 38.4 | 38.4 | 38.2 | 39.3 |
| 1956 | 37.8 | 45.3 | 38.6 | 42 | 38.5 | 38.4 | 38.5 | 39.3 |
| 1956 | 37.9 | 45.4 | 38.6 | 42 | 38.3 | 42.6 | 38.4 | 39.3 |
| 1964 | 38.1 | 44.7 | 38.2 | 47.5 | 42.5 | 42.5 | 43.3 | 43 |
| 1966 | 38.1 | 45.7 | 38.4 | 41.5 | 38.8 | 37.2 | 38.4 | 39.3 |
| 1975 | 38 | 44.7 | 38.2 | 47.5 | 42.5 | 42.7 | 44.3 | 44.2 |
| 1976 | 38.1 | 44.6 | 38.2 | 47.4 | 42.8 | 42.4 | 44.1 | 44.3 |
| 1976 | 38.2 | 44.6 | 38.3 | 47.4 | 42.8 | 40.85 | 44.1 | 44.3 |
| 1977 | 38.2 | 44.5 | 38.2 | 47.4 | 42.8 | 42.6 | 44.1 | 44.3 |
